# Ad-Apoptin-hTERTp-E1a Regulates Autophagy Through the AMPK-mTOR-eIF4F Signaling Axis to Reduce Drug Resistance of MCF-7/ADR Cells

**DOI:** 10.3389/fmolb.2021.763500

**Published:** 2021-11-19

**Authors:** Yaru Li, Yilong Zhu, Jicheng Han, Jinbo Fang, Zhiru Xiu, Shanzhi Li, Wenjie Li, Xia Yang, Ningyi Jin, Lili Sun, Xiao Li, Yiquan Li

**Affiliations:** ^1^ Academician Workstation of Jilin Province, Changchun University of Chinese Medicine, Changchun, China; ^2^ Changchun Veterinary Research Institute, Chinese Academy of Agricultural Sciences, Changchun, China; ^3^ Medical College, Yanbian University, Yanji, China; ^4^ Institute of Virology, Wenzhou University, Wenzhou, China; ^5^ Jiangsu Co-innovation Center for Prevention and Control of Important Animal Infectious Diseases and Zoonoses, Yangzhou, China; ^6^ Department of Head and Neck Surgery, Tumor Hospital of Jilin Province, Changchun, China

**Keywords:** adriamycin resistance, oncolytic adenovirus, breast cancer, toxicity, autophagy

## Abstract

Ad-VT (Ad-Apoptin-hTERTp-E1a) is a type of oncolytic adenovirus with dual specific tumor cell death ability. It can effectively induce cell death of breast cancer cells and has better effect when used in combination with chemotherapy drugs. However, it has not been reported whether Ad-VT reduces the resistance of breast cancer cells to chemotherapy drugs. The purpose of this study is to investigate the effect of Ad-VT on drug resistance of Adriamycin-resistant breast cancer cells. For this, the effects of different doses of Ad-VT on the resistance of breast cancer cells to Adriamycin were analyzed using qualitative and quantitative experiments *in vitro* and *in vivo*. The Ad-VT can reduce the resistance of MCF-7/ADR to adriamycin, which is caused by the reduction of MRP1 protein level in MCF-7/ADR cells after treatment with Ad-VT, and MRP1 can be interfered with by autophagy inhibitors. Subsequently, the upstream signal of autophagy was analyzed and it was found that Ad-VT reduced the resistance of cells to doxorubicin by reducing the level of mTOR, and then the analysis of the upstream and downstream proteins of mTOR found that Ad-VT increased the sensitivity of MCF-7/ADR cells to adriamycin by activating AMPK-mTOR-eIF4F signaling axis. Ad-VT can not only significantly induce cell death in MCF-7/ADR cells, but also improved their sensitivity to Adriamycin. Therefore, the combination of Ad-VT and chemotherapy drugs may become a new strategy for the treatment of breast cancer in overcoming Adriamycin resistance.

## Introduction

Cancer is one of the main causes of human death. In 2020, there will be 19.3 million cancer patients diagnosed worldwide, and 10 million people will die from cancer (Sung et al., 2021). The number of new cases of breast cancer (BC) is 2.26 million, surpassing lung cancer highest incidence rate. The mortality rate of breast cancer also ranks first among women. At present, the main treatment for breast cancer is a combination of surgery, radiotherapy, and chemotherapy. However, these mainstream therapies have serious side effects, are ineffective for metastatic patients, and have a post-therapeutic risk of recurrence. One of the most important reasons of recurrence is the development of resistance to chemotherapy drugs. Therefore, identifying the molecular mechanisms of drug resistance will have a great research significance and clinical value for the treatment of recurrent breast cancer.

At present, several studies showed that the overexpression of some ABC transporters is one of the important causes of tumor multidrug resistance (MDR) that represents the main obstacle for successful chemotherapies (Pluchino et al., 2012; Finlay et al., 2015). MDR does not only refer to drug resistance associated with one chemotherapeutic drug, but also implies drug resistance to other chemotherapeutic drugs with different functions, physical and chemical properties, and action targets. The multi-drug resistance that is mediated by the ABC family of transporters is the most important and crucial pathway that is used by tumor cells to resist chemotherapy. The representative MDR members of the ABC family include multidrug resistance gene 1 (MDR1), multidrug resistance associated protein (MRP1), and breast cancer resistance protein (BCRP) that is also known as ABCG2 (ABC transporter subfamily G) (Srinivasan et al., 2009). The common feature of these proteins is their capacity to provide energy through ATP hydrolysis, which helps tumor cells pump out a variety of anticancer drugs by reverse concentration gradient, leading to the decrease in the concentration of intracellular chemotherapeutic and the development of tumor cells’ drug resistance (Gottesman et al., 2002). Many studies have shown the presence of high expression levels of MDR 1, MPR 1, and BCRP in breast and lung cancers and leukemia, that were associated with poor chemotherapeutic responses (Coley, 2008; Corich et al., 2009; Li et al., 2010a). A clinical follow-up study showed that high expression levels of MDR1, MPR1, and BCRP were closely related to the prognosis of cancer patients (Li et al., 2009).

With the advances in molecular and cellular biology, and virology, gene therapy has become a new approach in cancer treatment. Oncolytic virus therapy shows great advantages and is expected to be a reliable method for breast cancer treatment. In a previous study, our team constructed a bispecific oncolytic adenovirus Ad-VT (Ad-Apoptin-hTERTp-E1a), which could specifically replicate and express the Apoptin gene in tumor cells (Li et al., 2010b). Apoptin is a type of apoptosis-inducing protein that is derived from chicken anemia virus (CAV). The oncolytic adenovirus Ad-VT has no cytotoxicity in normal cells, with the advantage of specifically inducing tumor cell apoptosis (Liu et al., 2012; Zhang et al., 2013; Qi et al., 2014; Yang et al., 2015).

The length and activity of human telomerase reverse transcriptase (hTERT) are related to cell senescence and immortalization. Most normal human cells lack telomerase activity due to the rate limitation of telomerase reverse transcriptase (hTERT) gene and the tight transcriptional inhibition of catalytic components. However, the expression of hTERT and the activation of telomerase are observed in up to 90% of human malignant tumors, resulting in unlimited proliferation (Shay and Bacchetti, 1997). Therefore, Ad-VT can only replicate and specifically kill a variety of tumor cells. Ad-VT showed excellent killing effects in a variety of tumor cells, including breast cancer cells (Chen et al., 2019; Wang et al., 2020). Meanwhile, when used in combination with different chemotherapy drugs, the anti-tumoral effect is improved. Therefore, we speculate that the combination of Ad-VT and chemotherapy drugs can result in synergistic effects, indicating that the oncolytic virus can reduce tumor cells’ drug resistance.

In this study, we analyzed the changes in different drug-resistant proteins in Adriamycin (ADR)-resistant MCF-7/ADR cells that were infected by Ad-VT to explore the effect of Ad-VT on Adriamycin-mediated resistance in breast cancer cells and the cellular pathways involved. The results of the study provide a new theoretical basis for the treatment of breast cancer using a treatment combination of oncolytic adenovirus and chemotherapy.

## Materials and Methods

### Reagent

All antibodies were purchased from CST and all inhibitors were acquired from MCE.

### Viruses, Cells, and Transfection

The BC cell line MCF-7 cell was purchased from the Chinese Academy of Sciences (cat. SCSP-669) and MCF-7/ADR cell was purchased from Beijing Beina Chuanglian Institute of Biotechnology (cat. BNCC340584). MCF-7 cells were cultured in DMEM medium, and MCF-7/ADR cells in RPMI 1640 medium with 500 ng/ml Adriamycin. The media were supplemented with 10% fetal bovine serum (FBS), 50 U/mL penicillin, and 50 U/mL streptomycin and cultured in an incubator at 37°C containing 5% carbon dioxide. The cell culture reagents were purchased from HyClone, GE Healthcare, and life sciences. The recombinant adenoviruses Ad-Apoptin-hTRETp-E1a (Ad-VT) and Ad-Mock were constructed and preserved in our laboratory (Li et al., 2010).

We purchased the control, AKT, eIF4E, and AMPK siRNAs from RIBOBIO (China). According to the efficacy of their knockdown effect, si-AKT, si-eIF4E, and si-AMPK were used in this study. The sequence of si-eIF4E, si-AKT and si-AMPK were 5′-AAG​CAA​ACC​TGC​GGC​TGA​TCT-3′, 5′-TTC​ATC​ATC​GAA​GTA​CCT-3′ and 5′-GAG​GAG​AGC​TAT​TTG​ATT​A-3′. The cells were transfected with 30 nM siRNA using Lipofectamine 3000 (Invitrogen) according to the manufacturer’s instructions.

The human MRP1, mTOR, and eIF4E cDNA were purchased form (You Biosciences, Hunan, China) and cloned into the pCDNA 3.1 plasmid. The mTOR, eIF4E plasmids and the corresponding empty vector were transfected into MCF-7/ADR cells using Lipofectamine 3000 reagent (Invitrogen) following the manufacturer’s protocol.

### Experimental Animals

Female BALB/c nude mice (4–5 weeks old) were purchased from Beijing vital River Laboratory Animal Technology Co., Ltd., and the animal experiment protocols were approved by the Institutional Animal Care and Use Committee (IACUC) of the Changchun University of Chinese Medicine. All surgeries were performed under anesthesia using sodium pentobarbital, and all efforts were made to minimize animal suffering. After the experiment, the remaining mice were euthanized. The applied method of euthanasia was an intraperitoneal injection of three times the anesthetic dose of sodium pentobarbital (150 mg/kg) and for 2–3 min. The euthanasia method was performed following the AVMA Guidelines for the Euthanasia of Animals.

### Colony Formation Assay

The cells were seeded in a 12-well plate and cultured for 24 h. Ad-VT and different reagents were added according to the different groups. After 48 h, the cell supernatants were discarded, and the cells washed 3 times with PBS. A total of 600 μL 0.4% crystal violet staining solution was added to each well. After 10 min incubation at room temperature, the staining solution was discarded, and the cells washed 3 times with PBS and placed in a dry environment prior taking photos for analysis the formation of cell colonies. (Jabir et al., 2020).

### Annexin V-FITC/PI Flow Detection Assay

The cells were seeded in a 12-well plate and cultured for 24 h. Ad-VT and different reagents were added according to the different groups. After 48 h, the cells were collected and resuspended in 500 ul staining solution (containing 5 μL FITC and 5 μL PI). The samples were stained in the dark for 20 min, at room temperature then transferred to the flow tube and properly labeled before flow cytometry (Ibrahim et al., 2021).

### CCK-8 Assay

The cells were seeded into 96-well plates and cultured for 24 h. Ad-VT and different reagents were added according to the different groups. After 48 h, each medium in the wells was replaced by a 100 μL culture solution containing the CCK-8 staining solution that was prepared at a ratio of 1:9 in a dark environment. The plate was then incubated at 37°C in a 5% CO_2_ incubator for 2 h. After incubation, a microplate reader was used to detect the OD value in each well at a wavelength of 450 nm (Hao et al., 2021). The detection of the inhibition rate of breast cancer cells was performed according to the following calculation formula:

Cell inhibition rate=(OD(average of the control group)−OD(average of the exp⁡erimental group))/OD(average of the control group)×100%

### Western Blot

The whole cell protein extract was prepared using RIPA cell lysate containing protease inhibitors. The same amount of protein samples was separated on a 10% SDS polyacrylamide gel and transferred to a PVDF membrane. The membranes were blocked with 5% skim milk for 1–2 h, then incubated overnight at 4°C with primary antibodies followed by incubations with 2 h incubations with secondary antibodies at room temperature. Finally, enzyme-linked chemiluminescence (ECL) was used for the detection. The results were quantitatively analyzed with chemiluminescence and fluorescence imaging systems. The detailed steps were performed as previously described (Chen et al., 2019).

### Immunofluorescence

The cells were seeded in a 12-well plate (with sterile cell slides) and cultures for 24 h. Ad-VT and different reagents were added according to the different groups. After 48 h, the wells were fixed with 4% paraformaldehyde for 15 min, permeabilized with 0.5% Triton X-100, blocked with 1% bovine serum albumin (BSA) for 2 h, incubated with the primary antibody overnight at 4°C, and wash 3 times with PBS. Secondary antibodies labeled with FITC or CY3 were incubated for 2 h, and the cell slides analyzed by Zeiss confocal microscope (Jabir et al., 2021).

### Animal Assay

The MCF-7/ADR cells (3×10^6^) were inoculated into the chest subcutaneously of 5-week-old BALB/c nude mice to establish a tumor model (*n* = 6). The tumors’ size was measured, and survival checked every week. After 28 days of treatment, the animals were euthanized, and each tumor was fixed with formalin and subjected to immunohistochemical staining. The growth curve was drawn, and the tumor volume calculated as follows:

$$Tumor\;volume\left(mm^{3}\right)=\left(long\;diameter\;of\;tumor\times short\;diameter\;of\;tumor^{2}\right)/2$$

The inhibition rate was calculated using the formula:

Tumor inhibition rate=(1−treatment group tumor volume/control tumor volume)×100%

### Immunochemistry

The tissue sections were deparaffinized, rehydrated, and incubated in 3% H_2_O_2_ methanol for 15 min to eliminate endogenous peroxidase activity. The slides were put in 0.01M sodium citrate buffer (pH 6.0) and incubated at 95°C for 20 min to perform antigen retrieval. Following this step, the slides were incubated with the primary antibody overnight at 4°C. After the incubation, another incubation was performed with the secondary antibody for 1 h at room temperature, followed by DAB staining, and the sections were counterstained with hematoxylin. The mean density analysis method was used for the evaluation of the immunostaining: The Image-Pro Plus 6.0 software was used to select the same yellow brown as a unified standard for judging positivity of the immunostaining in all photos, and analyze each photo to obtain the cumulative light of each photo, the density value (IOD) and the pixel area of the tissue (AREA). This step was followed by the calculation of the average optical density value IOD/AREA (Mean Density).

### Statistical Analysis

The data were presented as mean ± standard error of the mean (SEM). GraphPad Prism 6.0 software was used to perform the statistical analyses of unpaired two-tailed Student’s t-tests or analysis of variance (ANOVA). *p* < 0.05 was considered statistically significant. *p < 0.05, **p < 0.01, ***p < 0.001, ****p < 0.0001.

## Results

### Gemcitabine and Taxol Sensitivity

MCF-7 and MCF-7/ADR cell lines were treated with different concentrations of adriamycin, respectively. The results showed that the IC_50_ of MCF-7 and MCF-7/ADR cells to adriamycin was 347 ± 68.5 and 5644 ± 341.8 nM (Table 1). The resistance index of MCF-7/ADR cells to adriamycin was 16. It is suggested that MCF-7/ADR is not sensitive to adriamycin.

**TABLE 1:** The resistance index (RI) of adriamycin in MCF-7/ADR cells (mean ± SD, *n* = 3).

Compounds	IC50(nM)		RI
	MCF-7	MCF-7/ADR	
Adriamycin	347 ± 68.5	5644 ± 341.8	16

### Ad-VT Induces Cell Death in MCF-7 Cells and MCF-7/ADR Cells

**FIGURE 1:**
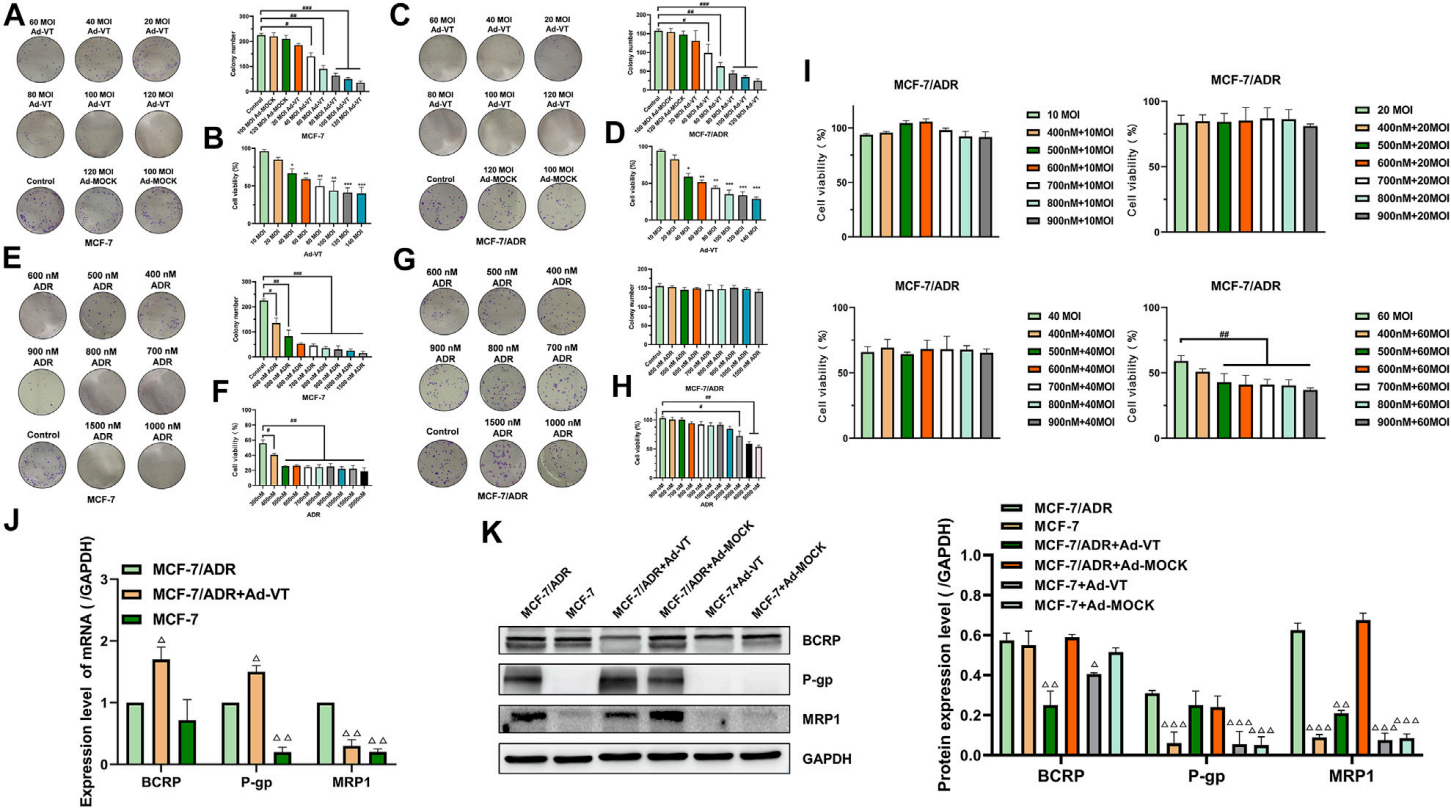
Synergistic effect of Ad-VT and ADR. The colony formation and CCK-8 assay analyzes the cell viability of MCF-7 **(A,B)** and MCF-7/ADR **(C,D)** cells after infection with Ad-VT. The colony formation and CCK-8 assay analyzes the cell viability of MCF-7 **(E,F)** and MCF-7/ADR **(G,H)** cells after adding with adriamycin (ADR). **(I–K)** CCK-8 and western blot assay analyzes the effect of Ad-VT on ADR resistance in MCF-7/ADR cells. Data were representative of three independent experiments (*n* = 3). The unpaired Student’s test-test was used. ^*#*^ *p* < 0.05, ^*##*^ *p* < 0.01, ^*###*^ *p* < 0.001. (*p <0.05, **p <0.01, ***p <0.001) when compared with 10 MOI Ad-VT. (^△^ *p* < 0.05, ^△△^ *p* < 0.01, ^△△△^ *p* < 0.001) when compared with MCF-7/ADR.

Ad-VT can specifically replicate and express apoptin protein in tumor cells. It can induce specific apoptosis of tumor cells. In this experiment, colony formation assay, and the CCK-8 assay showed that Ad-VT significantly indues cell death in MCF-7 and MCF-7/ADR cells, and this effect was higher in MCF-7/ADR cells compared to that in MCF-7 cells (Figures 1A–D). Ad-VT had also a stronger effect with a MOI of 40, reaching approximatively 40% in MCF-7/ADR cells and 30% in MCF-7 cells. These results demonstrate that Ad-VT induces a significant cell death in MCF-7 cells, which is not associated with MCF-7 cells’ resistance to Adriamycin.

### Ad-VT Reduces the Resistance of MCF-7/ADR Cells to Adriamycin

Our results indicated that MCF-7/ADR cells do not respond to Adriamycin when used at concentrations of 500–2000 nM and in combination with Ad-VT (Figures 1E–H). To further investigate a potential relationship between Ad-VT and Adriamycin resistance, the ad-VT treatment dose was increased, and we found that after increasing the dose to 60 MOI, the treatment concentration of 500 nM Adriamycin could induce cell death in MCF-7/ADR cells (Figure 1I). Therefore, it is suggested that 60 MOI Ad-VT can reduce the resistance of MCF-7/ADR cells to Adriamycin.

We also analyzed the expression of multidrug resistance-related proteins in MCF-7 and MCF-7/ADR cells and found that MRP1 and P-gp were not expressed in MCF-7 but were highly expressed in MCF-7/ADR cells. After adding Ad-VT, we found that Ad-VT significantly reduces the expression of MRP1 in MCF-7/ADR cells. A decrease in the level of P-gp protein was also observed; however, the difference was not significant (Figures 1J,K). Similar results were obtained at the transcriptional level, where the addition of Ad-VT significantly reduced the MRP1 gene expression level. These results indicate that Ad-VT can reduce the drug resistance of MCF7/ADR cells to Adriamycin, which may be caused by the reduction of MRP 1 protein.

### Ad-VT Induced Apoptosis and Autophagy in MCF-7/ADR and MCF7 Cells

**FIGURE 2:**
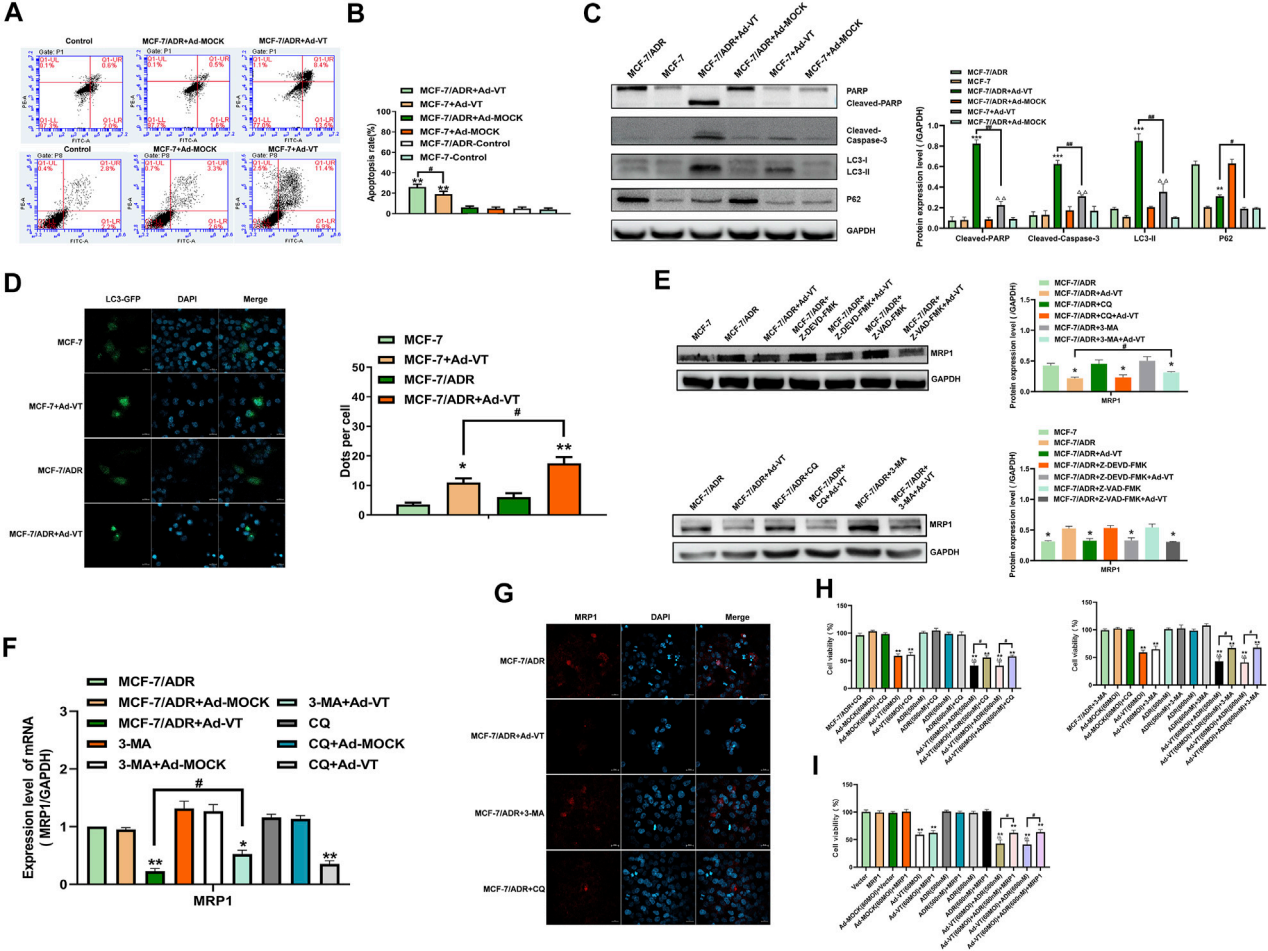
Detection of the levels of Ad-VT-induced autophagy and apoptosis in MCF-7/ADR cells. **(A,B)** The apoptotic levels of MCF-7 and MCF-7/ADR cells were detected by flow cytometry after Annexin V-FITC/PI staining. **(C)** Western blot analyzes of the protein expression levels of autophagy and apoptosis-related proteins. **(D)** Analyze of changes in autophagic flux using a LC3-GFP plasmid. **(E,F)** Analyze of MRP expression level using inhibitors of apoptosis and autophagy. **(G)** Analysis of autophagy effects on the resistance of Ad-VT-treated MCF-7/ADR cells to ADR by immunofluorescence staining. **(H)** Using autophagy inhibitors to analyze whether autophagy affects the resistance of Ad-VT-treated MCF-7/ADR cells to ADR. **(I)** Analysis of the change in the resistance of Ad-VT-treated MCF-7/ADR cells to ADR after MRP1 overexpression in MCF-7/ADR cells. The scale bar equals 20 µm. Data were representative of three independent experiments (*n* = 3). The unpaired Student’s test-test was used. ^*#*^ *p* < 0.05, ^*##*^ *p* < 0.01. (*p <0.05, **p <0.01, ***p <0.001) when compared with MCF-7/ADR.

In our previous studies, we found that Ad-VT induces apoptosis of tumor cells and also causes autophagy (Chen et al., 2019). In this study, we performed an Annexin V experiment and show that treatment with 60 MOI Ad-VT induces more apoptosis in MCF-7/ADR cells compared to that in MCF-7 cells (Figures 2A,B). After detection of the protein expression levels of PARP and caspase-3, we found that the cleavage levels of the two proteins in MCF-7/ADR cells were higher than that in MCF-7 cells after treatment with Ad-VT (Figure 2C).

Then we analyzed the expression levels of autophagy-related proteins and found that the expression of LC3-II in MCF-7/ADR cells that were infected with 60 MOI Ad-VT was significantly higher than that in MCF-7 cells (Figure 2C). We also carried out an GFP-LC3 transfection test and found that the number of MCF-7/ADR cells with an LC3 green fluorescence was significantly higher than that in MCF-7 cells (Figure 2D). These results suggest that Ad-VT can induce strong apoptosis and autophagy in MCF-7/ADR cells, which may be related to an Ad-VT-mediated reduction of drug resistance in MCF-7/ADR cells.

### Ad-VT Causes Changes in the Resistance of MCF-7/ADR Cells to Adriamycin Through Changes in Autophagy

As Ad-VT induces autophagy and apoptosis, we analyzed whether these two types of programmed cell death are related to drug resistance. Therefore, we analyzed the expression of drug resistant proteins in MCF-7/ADR cells that were treated with 60 MOI Ad-VT in the presence of different inhibitors of autophagy and apoptosis. The results showed that the inhibition of autophagy in these cells increases MRP1 level of expression and that the inhibitory effect of 3-MA was higher than that of CQ. However, the inhibition of apoptosis had no effect on MRP1 level of expression (Figures 2E–G). Next, a CCK-8 test was performed on MCF-7/ADR cells that were treated with 60 MOI Ad-VT, transfected with MRP1 expression plasmid, and treated with autophagy inhibitors. The results showed that the killing effect of Ad-VT-induced adriamycin on MCF-7/ADR cells was inhibited after inhibiting autophagy and MRP1 overexpression, and the effect of 3-MA was higher than that of CQ (Figures 2H,I). These results indicate that Ad-VT influences the resistance of MCF-7/ADR cells to Adriamycin by inducing autophagy.

### Ad-VT Causes Changes in the Resistance of MCF-7/ADR Cells to Adriamycin Through Changes of mTOR

**FIGURE 3:**
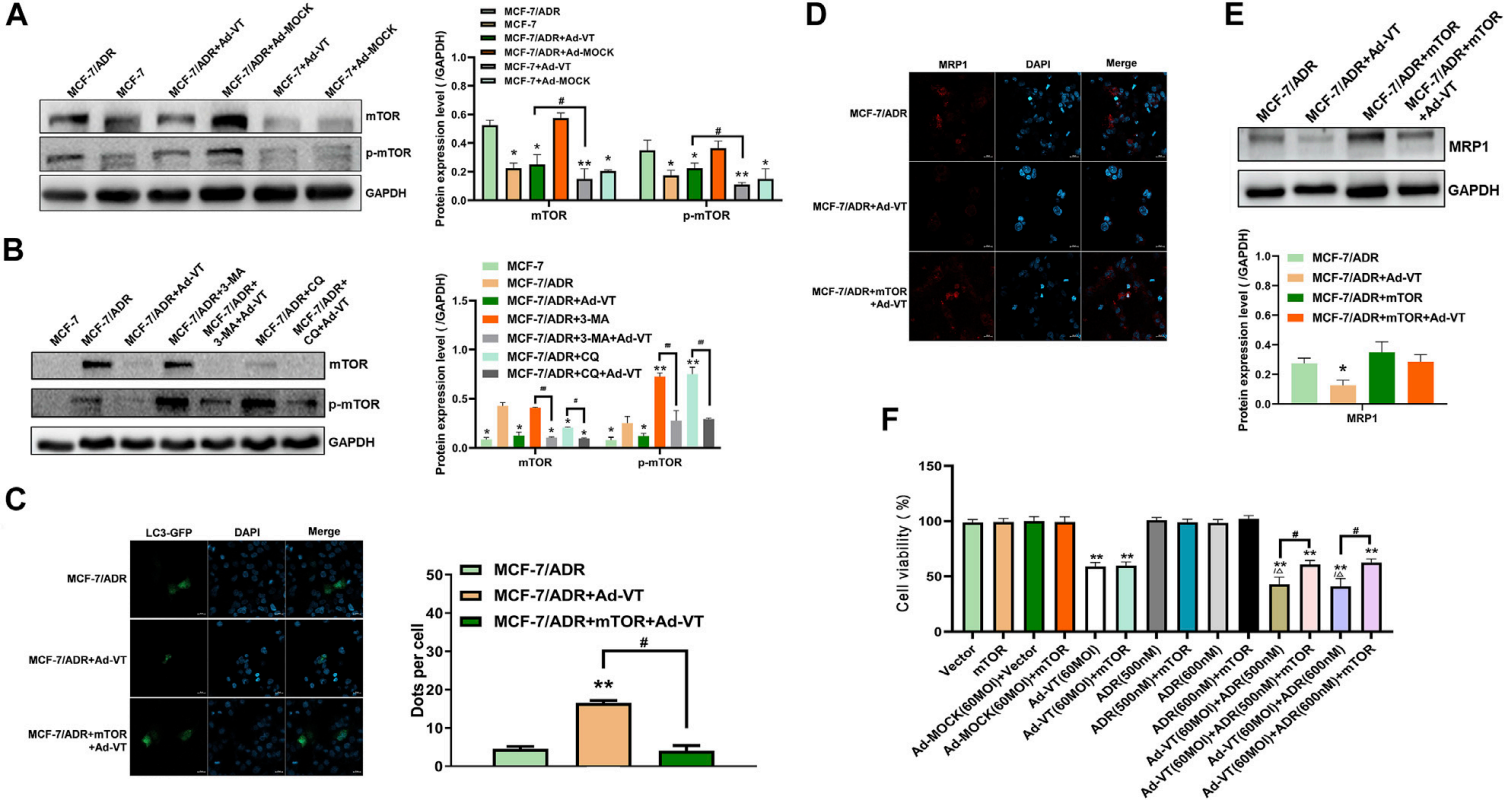
Detection of mTOR role in ADR resistance. **(A)** Western blot analyzes of the changes of mTOR protein level in MCF-7 and MCF-7/ADR cells after infection with Ad-VT. **(B)** Western blot analysis of the changes in mTOR protein level in MCF-7/ADR cells after inhibiting autophagy. **(C)** Analysis of the changes in autophagic flux with LC3-GFP plasmid after mTOR overexpression. **(D,E)** Western blot and immunofluorescence staining analyses of the changes in MRP1 protein level after mTOR overexpression. **(F)** Analysis of the change in the resistance of Ad-VT-treated MCF-7/ADR cells to ADR after mTOR overexpression in MCF-7/ADR cells. The scale bar equals 20 µm. Data were representative of three independent experiments (*n* = 3). The unpaired Student’s test-test was used. ^*#*^ *p* < 0.05. (*p <0.05, **p <0.01) when compared with MCF-7/ADR. (^△^ *p* < 0.05) when compared with MCF-7/ADR + Ad-VT.

mTOR activation is frequently reported in many human cancers, including lung, pancreatic, gastric, and breast cancers. In addition, mTOR seems to play an important role in cancer occurrence, development, and chemotherapy. Therefore, we studied whether the activation of mTOR was related to the change in Ad-VT induction of MCF-7/ADR cells’ resistance to Adriamycin. The results of western blot showed that mTOR is activated in MCF-7/ADR cells, but not in MCF-7 cells, and that the activity of mTOR in MCF-7/ADR cells is also inhibited after adding 60 MOI Ad-VT (Figure 3A). It is suggested that Ad-VT may induce a change in the resistance of MCF-7/ADR cells to Adriamycin through inhibiting the activation of mTOR. We also analyzed mTOR protein expression after adding an autophagy inhibitor and found that the inhibition of autophagy significantly increases the activation of mTOR, and that treatment of these cells with 60 MOI Ad-VT treatment reverses the effects caused by the autophagy inhibitors (Figure 3B). Then we overexpressed mTOR in these cells and found mTOR increased the expression of MRP1 and also decreased the level of the autophagy (Figures 3C–E). The results of CCK-8 also showed that killing effect of Adriamycin on MCF-7/ADR cells is inhibited after mTOR overexpression (Figure 3F). The combination of the above results with the results of autophagy inhibition shows that Ad-VT causes a change in MCF-7/ADR cells’ resistance to Adriamycin through mTOR.

### Ad-VT Changes the Resistance of MCF-7/ADR Cells to Adriamycin *via* AMPK Pathway

**FIGURE 4:**
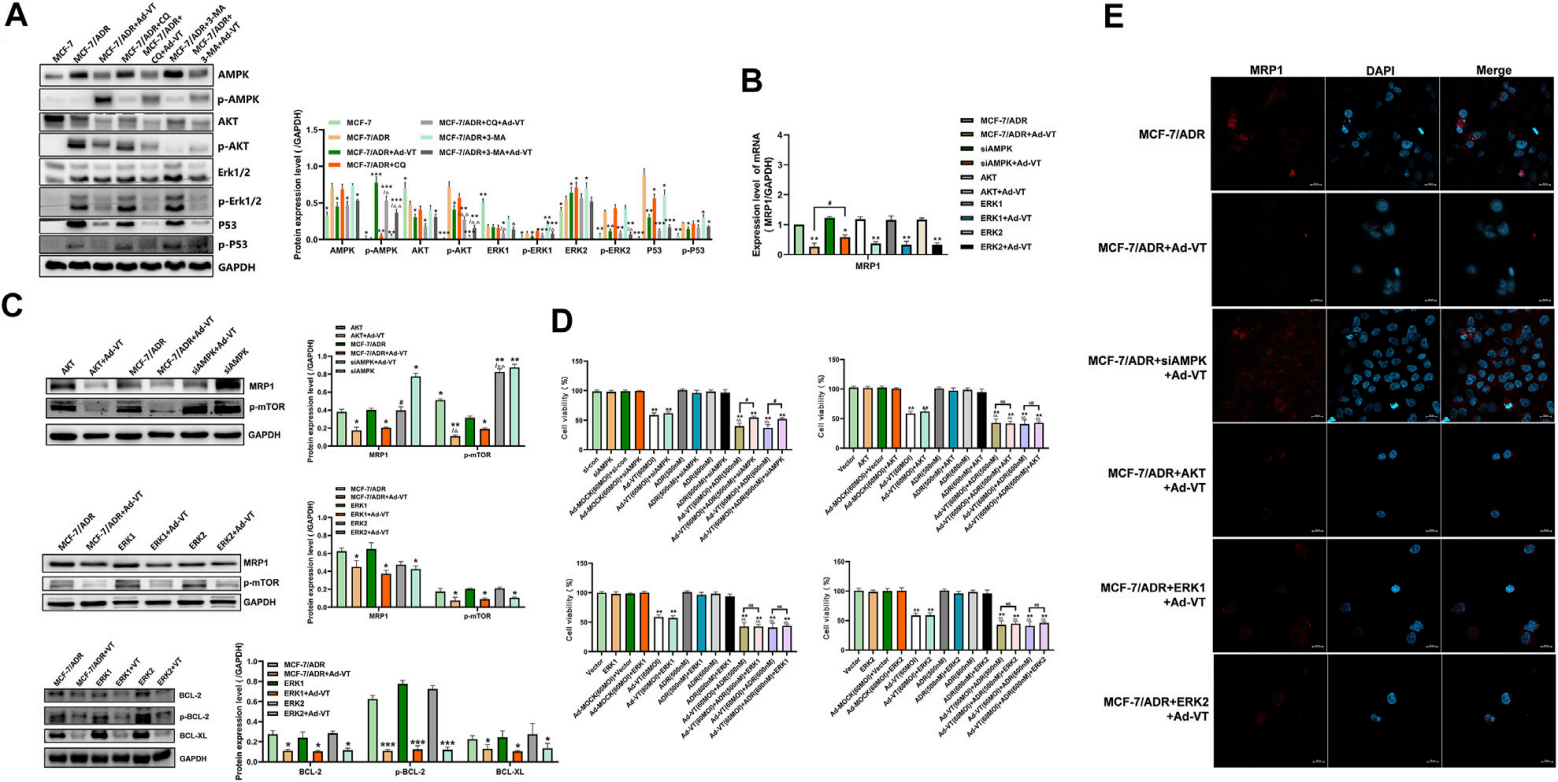
Detection of the role of proteins acting upstream of mTOR in ADR resistance. **(A)** Western blot analysis of the changes in expression levels of proteins acting upstream of mTOR in MCF-7 and MCF-7/ADR cells after adding Ad-VT. **(B,C)** Western blot and qPCR analyses of the changes in MRP1 protein levels after AKT/ERK1/2 overexpression and AMPK silencing. **(D)** Analyze of the change in the resistance of Ad-VT-treated MCF-7/ADR cells to ADR after AKT/ERK1/2 overexpression and AMPK silencing in MCF-7/ADR cells. **(E)** Immunofluorescence staining analysis of the changes in MRP1 protein level after AKT/ERK1/2 overexpression and AMPK silencing. The scale bar equals 20 µm. Data were representative of three independent experiments (*n* = 3). The unpaired Student’s test-test was used. ^*#*^ *p* < 0.05. (*p <0.05, **p <0.01, ***p <0.001) when compared with MCF-7/ADR. (^△^ *p* < 0.05, ^△△^ *p* < 0.01) when compared with MCF-7/ADR + Ad-VT.

After analyzing the role of mTOR in drug resistance, we analyzed the upstream protein of mTOR. Through the detection of protein level, it was found that Ad-VT activated AMPK and inhibited AKT, ERK1/2 and p53 in MCF-7/ADR cells. Studies showed that the activation of AMPK and the inhibition of AKT and ERK 1/2 could inhibit the activation of mTOR (Figure 4A). So we silenced and overexpressed these three proteins. The results showed that the expression of MRP1 after silencing AMPK with the addition of 60 MOI Ad-VT was higher than that after overexpression of AKT and ERK1/2, which may indicate that AMPK is the key protein that causes Ad-VT to reduce adriamycin resistance (Figures 4B,C,E). This result was also confirmed in the subsequent analysis of transcription level. We also conducted the CCK-8 test, and found that the killing effect of Ad-VT-induced adriamycin on MCF-7/ADR cells was significantly inhibited after AMPK was silenced, but there was no significant change after overexpression of AKT and ERK1/2 (Figure 4D). Subsequently, we also analyzed the reticulum-related proteins Bcl-2 and Bcl-XL downstream of ERK1/2. It was found that overexpression of ERK1/2 did not significantly affect the downstream BCL-XL and BCL-2 expression levels (Figure 4C). The above results suggest that the change of Ad-VT mainly leads to in mTOR expression through a change in AMPK expression, which leads to a change in MCF-7/ADR cells’ Adriamycin resistance.

### Ad-VT Causes Changes in MCF-7/ADR Cells’ Resistance to Adriamycin Through the AMPK-mTOR-eIF4F Signaling Axis

**FIGURE 5:**
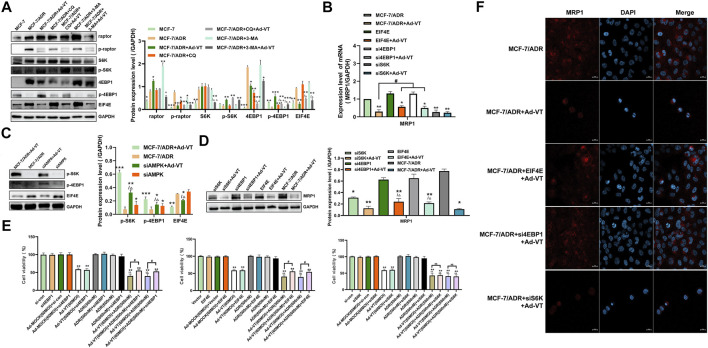
Detection of the role of proteins acting downstream of mTOR in ADR resistance. **(A)** Western blot assay analysis of the changes in the expression of proteins acting downstream of mTOR in MCF-7 and MCF-7/ADR cells after infection with Ad-VT. **(B,D)** Western blot and qPCR analyses of the changes in MRP1 protein level after eIF4E overexpression and S6K/4EBP1 silencing. **(C)** Western blot analysis of the changes of proteins acting downstream of mTOR in MCF-7/ADR cells after AMPK silencing. **(E)** Analysis of the change in the resistance of Ad-VT-treated MCF-7/ADR cells to ADR after eIF4E overexpression and S6K/4EBP1 silencing in MCF-7/ADR cells. **(F)** Immunofluorescence staining analysis of the changes in MRP1 protein level after eIF4E overexpression and S6K/4EBP1 silencing. The scale bar equals 20 µm. Data were representative of three independent experiments (*n* = 3). The unpaired Student’s t-test was used. ^*#*^ *p* < 0.05. (*p <0.05, **p <0.01, ***p <0.001) when compared with MCF-7/ADR. (^△^ *p* < 0.05, ^△△^ *p* < 0.01) when compared with MCF-7/ADR + Ad-VT.

Following the analysis of the upstream signaling pathway of mTOR, we also investigated downstream mTOR proteins. Among the reported proteins involved in mTOR-induced drug resistance, S6K and eIF4E play the most important roles (Tee and Blenis, 2005). The results showed that in MCF-7 and MCF-7/ADR cells, S6K was not activated, but it was activated after adding 60 MOI Ad-VT (Figure 5A). However, previous reports showed that the activation of S6K leads to an increase in drug resistance; thus, we silenced the S6K in MCF-7/ADR cells found that its silencing reduces MRP1 expression level and Adriamycin resistance in MCF-7/ADR cells. However, we also found that treatment of the cells with 60 MOI Ad-VT activates S6K. These results were contradictory and suggest that Ad-VT does not cause a change in Adriamycin resistance in MCF-7/ADR cells through in S6K expression (Figures 5B,F). Then, we overexpressed eIF4E in MCF-7/ADR cells that were also treated with 60 MOI Ad-VT and found that MRP1 level increased significantly in these cells, indicating that eIF4E is a key protein involved in Ad-VT-mediated reduction in Adriamycin resistance (Figures 5B,F). This result was also confirmed in the subsequent transcriptional analysis. The CCK-8 test showed that the apoptotic effect of Ad-VT on eIF4E expressing cells was decreased, and that the apoptotic effect of Adriamycin on MCF-7/ADR cells was suppressed. We also analyzed eIF4E protein expression following AMPK silencing and found that its level was significantly decreased; however, after treatment of cells with 60 MOI Ad-VT, its level significantly increased (Figures 5C,D). It was reported that mTOR regulates the eIF4E signal axis downstream of 4EBP1, leading to a change in eIF4E expression (Sarbassov et al., 2005; Hsieh et al., 2012). Therefore, we also analyzed 4EBP1 protein expression and found that its expression level was opposite to that of eIF4E protein expression. After silencing 4EBP1 in MCF-7/ADR cells, the expression of eIF4E and MRP1 increased, and the resistance of MCF-7/ADR cells to Adriamycin after Ad-VT infection increased (Figures 5E,F). The above results suggest that Ad-VT can change the resistance of MCF-7/ADR cells to Adriamycin through the AMPK-mTOR-eIF4F signal axis.

### Ad-VT Induces the Changes of Adriamycin Resistance in MCF-7/ADR Cells *In-vivo*

**FIGURE 6:**
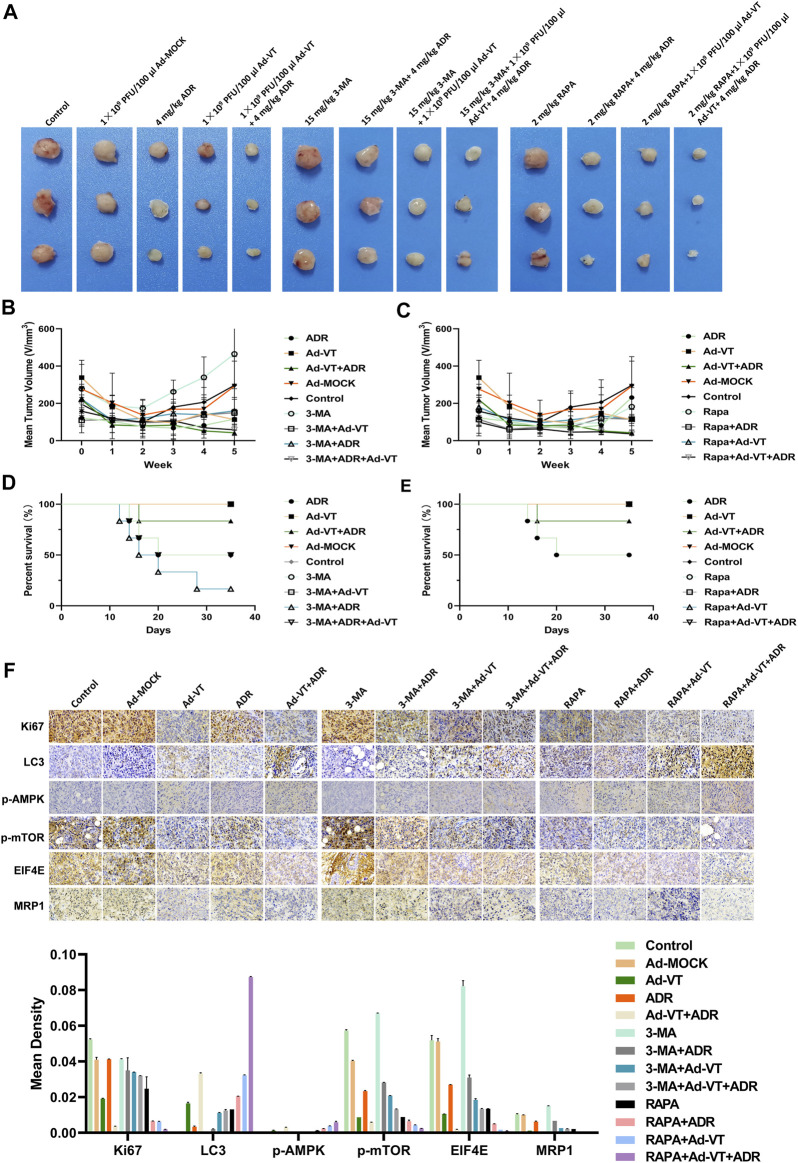
Detection of the effect of Ad-VT on Adriamycin resistance *in vivo*. **(A,B,C)** Length and width of xenograft tumors that were weekly measured for 5 weeks using Vernier calipers. The average tumor inhibition was calculated using the formula: (1—treatment group tumor volume/control tumor volume) × 100%. **(D,E)** The survival rate of tumor-bearing mice was daily recorded for 5 weeks. **(F)** Expression of LC3, p-AMPK, p-mTOR, eIF4E, MRP1, and Ki67 in the xenograft tumors tissues were detected by IHC. The scale bar equals 50 µm. Data were representative of three independent experiments (*n* = 3).

We constructed a subcutaneous tumor-bearing model of MCF-7/ADR cells in nude mice. The results showed no obvious change in the tumor volume in ADR group compared with that in the control group, but the tumors’ volume significantly decreased after adding Ad-VT, and there was a significant difference compared with that in the Ad-VT group. After adding an autophagy inhibitor, the tumors’ volume reduction in the ADR + Ad-VT group was significantly reduced (Figures 6A–C). On the contrary, after the addition of the autophagy promoter, rapamycin (RAPA), the reduction of the tumors’ volume in the ADR + Ad-VT group was the most significant (Figures 6A–C). We also found that the addition of an autophagy inhibitor increases the toxicity of Adriamycin, resulting in the death of mice, but no death was found after the addition of autophagy enhancers (Figures 6D,E). The immunohistochemistry results showed that adding Ad-VT can inhibit the expression of MRP1, eIF4E and p-mTOR, and increase the expression of p-AMPK (Figure 6F). Opposite results were obtained by adding an autophagy inhibitor. These results are consistent with the results obtained *in vitro*. These results indicate that Ad-VT can also cause changes in the resistance of MCF-7/ADR cells to adriamycin *in vivo*, and that the regulation of autophagy can significantly affect the effect of Ad-VT.

## Discussion

The ATP binding cassette family of transporters (ATP-binding cassette transporter, ABC) is a large group of ATP driven pumps, which consists of two transmembrane domains and two cytoplasmic ATP binding domains. According to the homology of amino acid sequence and domain sequence of the conserved region, it was found that there are 49 members of human ABC superfamily belonging to 7 subfamilies of ABCA-ABCG (Higgins, 1992; Sarbassov et al., 2005). Under physiological conditions, ABC transporters are widely distributed in various tissues and organs of the human body, where they transport ions, amino acids, nucleic acid, polysaccharides, polydermis, proteins, drugs, and other substances, and participate in the absorption, distribution, and excretion of these substances in the human body. Therefore, they play an important role in stabilizing the internal environment. Recent studies have found that some ABC transporters are abnormally expressed in human malignant tumors, which correlate with the occurrence and development of tumors, the emergence of chemotherapy multidrug resistance, and poor prognosis in cancer patients (Szakacs et al., 2006).

To investigate whether Ad-VT induces drug resistance to Adriamycin in the breast cancer cell line, MCF-7/ADR cells, we performed an apoptotic test following treatment of the cells with a combination of Ad-VT and Adriamycin at different concentrations. The results showed that increasing the dose of Ad-VT to 60 MOI can induce Adriamycin-mediated apoptosis (500 nM) of MCF-7/ADR cells, through reducing their drug resistance to Adriamycin.

In breast cancer cells, there are many types of drug-resistant proteins such as BCRP, P-gp, and MRP1. MDR1 is one of the earliest and most widely researched multidrug resistance gene. It was first identified in Chinese hamster ovary cells and is a member of the ABC transporter superfamily, also known as ABCB1. The membrane glycoprotein that is encoded by ABCB1 is named P-glycoprotein (P-gp). Its structure is mainly composed of two transmembrane regions and two nucleotide binding regions (Locher, 2009; Iakusheva et al., 2014). It has been found that P-gp can utilize the energy obtained from ATP hydrolysis to discharge toxic metabolites or exogenous substances out of the cells appositively to concentration gradient; thus, protecting the body cells from toxic substances (Iakusheva et al., 2014). MRP1 is a drug-resistant protein that was identified in the Adriamycin-resistant small cell lung cancer cell line, H69/AR, in 1992. It also belongs to the superfamily of ABC transporters and is called ABCC1. MRP1 is mainly located on the plasma membrane of cytoplasmic organelles, such as endoplasmic reticulum, Golgi apparatus and vesicles that are involved in cytoplasmic transport. It is also expressed on the cell membrane of normal cells and on the cell membranes and in the cytoplasm of tumor cells. Under physiological conditions, MRP 1 is expressed at a low level in most human tissues, and is involved in oxidative stress, detoxification and defense against exogenous poisons (Cole et al., 1992; Cole, 2014). According to previous reports, the MRP1-mediated drug resistance in tumor cells is mainly due to the isolation of chemotherapy drugs in cytoplasm vesicles, which makes chemotherapy drugs unable to reach nuclear targets, resulting in drug resistance. Apart from its inability to transport paclitaxel, the drug resistance spectrum of MRP1 is similar to that of MDR1 (Cole, 2014). BCRP (ABCG 2) is also a member of the ABC transporter superfamily and the first Adriamycin-resistant protein that was identified in the breast cancer resistant cell line, MCF-7/AdrVP, and therefore, was named as breast cancer resistant protein (Oostendorp et al., 2009; Drozdzik et al., 2014). Like MDR 1 and MRP 1, it can transport substances out of cells using the energy obtained from ATP hydrolysis, but the monomer ABCG 2 has no transport function, and requires the formation of a homodimer or homopolymer to complete substrates’ transport.

We detected these 3 types of drug-resistant proteins and found that the addition of Ad-VT significantly reduces the expression of MRP1, but there was no decrease in the expression of BCRP and P-gp. At the transcription level, we also found that the addition of Ad-VT only reduces the copy number of MRP1 but had no effect on BCRP and P-gp. This suggests that the Ad-VT-mediated decrease in drug resistance to Adriamycin was caused by the decrease in MRP1 protein expression.

It was reported that autophagy is closely related to drug resistance and that it regulates the survival and death of cancer cells (Hanahan and Weinberg, 2011). Traditionally, the relationship between autophagy and drug resistance has been divided into two distinct mechanisms and their related effects: One is associated with its protective mechanism against tumor drug resistance, and the other is related to autophagy-induced cell death, which increases tumor sensitivity to apoptosis (Li et al., 2012; Schwartz-Roberts et al., 2013; Li et al., 2017).

Ad-VT has been shown to promote the apoptosis and autophagy of MCF-7 cells (Chen et al., 2019), and therefore, we first detected the changes in apoptosis and autophagy in MCF-7 and MCF-7/ADR cells by investigating the expression of related key proteins. We found that autophagy and apoptosis in MCF-7/ADR cells were higher compared to those in MCF-7 cells following infection with Ad-VT. Therefore, we speculated that the aggravation of autophagy and apoptosis by the Ad-VT infection may have caused the change in cell death resistance. Then we analyzed different inhibitors of apoptosis and autophagy and found that the inhibition of apoptosis does not cause changes in the expression of drug-resistant proteins after adding Ad-VT, while the inhibition of autophagy increases their expression levels. These results indicate that our speculation may be correct and suggest that autophagy plays a key role in causing the change in drug resistance. They also indicate that autophagy my be mediating the Ad-VT induced cell death in MCF 7/ADR cells. The addition of an autophagy inhibitor and the CCK-8 test confirmed these observations.

When using autophagy inhibitors to analyze the changes of drug-resistant proteins, we found that the effect of 3-MA was higher than that of CQ, and thus, we anchored the research direction to the early stage of autophagy, in which the mTOR protein plays an important role in various cellular activities and in drug resistance. Then, we analyzed whether the activation of mTOR was related to the change in MCF-7/ADR cells’ resistance to Adriamycin that is induced by Ad-VT. The results showed that mTOR was activated in MCF-7/ADR cells, but not in MCF-7 cells, and that the activity of mTOR in MCF-7/ADR cells was inhibited infection with Ad-VT. The overexpression of MTOR also increased the expression of MRP 1, which inhibited the apoptotic effect of Adriamycin on MCF-7/ADR cells. These results indicate that Ad-VT causes a change MCF-7/ADR cells’ drug resistance to Adriamycin through mTOR.

After analyzing the role of mTOR in drug resistance, we analyzed the expression of mTOR upstream proteins. The results showed that after silencing AMPK and the addition of Ad-VT to the cells, the expression of MRP1was higher than that of cells with AKT and ERK1/2 overexpression, indicating that AMPK protein is the key protein that leads Ad-VT infected MCF-7/ADR cells to decrease their resistance to Adriamycin. The CCK-8 test also showed that the cell death effect of Ad-VT on AMPK knockdown MCF-7/ADR cells was lower than that of MCF-7/ADR cells overexpressing AKT and ERK 1/2.

After analyzing the expression of mTOR upstream proteins, we also analyzed the expression of mTOR downstream proteins. The activation of S6K and eIF4E can increase the drug resistance of tumor cells to chemotherapy drugs (Tee and Blenis, 2005). Therefore, we have analyzed their protein expression levels in this experimental setting. The results showed that S6K is not activated in MCF-7 and MCF-7/ADR cells; however, Ad-VT infection of the cells activated its expression. Subsequently, the silencing of S6K did reduce the expression level of MRP1 and Adriamycin resistance in MCF-7/ADR cells, but the infection with Ad-VT activated S6K. These results were contradictory and suggested that Ad-VT did not cause the change of Adriamycin resistance in MCF-7/ADR cells through S6K.

However, the expression of eIF4E was significantly decreased after infection with Ad-VT, and the level of MRP1 significantly increased after eIF4E overexpression and infection of the cells with Ad-VT. In the subsequent CCK-8 test, we found that in the cells overexpressing eIF4E, the cell death effect of Ad-VT decreases and the apoptotic effect of Adriamycin on MCF-7/ADR cells was inhibited. After addition of an autophagy inhibitor and the silencing AMPK, we found that the level of eIF4E protein, which was significantly decreased after adding Ad-VT, significantly increased. Studies have shown that mTOR regulates the eIF4E signaling axis downstream 4EBP1, which leads to a change in eIF4E expression (Sarbassov et al., 2005; Hsieh et al., 2012), and therefore, we also analyzed the expression of 4EBP1. Indeed, its expression level was opposite to that of the eIF4E protein, and the expression of eIF4E and MRP1 increased after 4EBP1 silencing. The resistance of MCF-7/ADR cells to adriamycin after Ad-VT infection was also increased.

Although extensive experiments were performed in this study to reveal the potential of Ad-VT in MCF-7/ADR cells in reducing the resistance of cells to adriamycin, there are still limitations here. In this study, we only analyzed the drug resistance of MCF-7 cells. In future studies, different types of breast cancer cells should be added, and other tumor cells should be added to perform a more in-depth study on the role of Ad-VT in reducing drug resistance.

In summary, Ad-VT can not only induce the cell death of MCF-7/ADR cells, but also reduce drug resistance to Adriamycin by increasing autophagy, which is caused by the AMPK-mTOR-4EBP1-eIF4F signaling axis. We suggest that the oncolytic adenovirus Ad-VT plays an important role in the combination of chemotherapy drugs and could be used as a drug to cell death in breast cancer cells.

## Data Availability

The original contributions presented in the study are included in the article/Supplementary Material, further inquiries can be directed to the corresponding author.
