# Supplementary figures and images for: Ad-Apoptin-hTERTp-E1a Regulates Autophagy Through the AMPK-mTOR-eIF4F Signaling Axis to Reduce Drug Resistance of MCF-7/ADR Cells
Source: Front Mol Biosci. 2021 Nov 19;8:763500. doi: 10.3389/fmolb.2021.763500 (PMC8640141; doi:10.3389/fmolb.2021.763500)

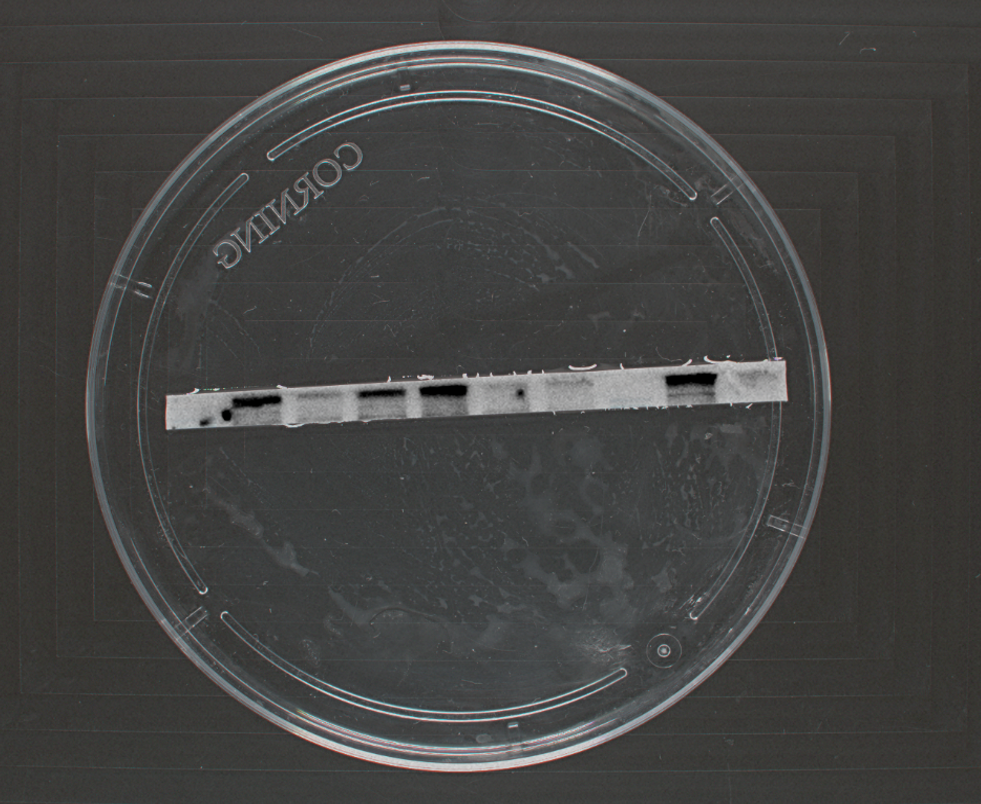

Supplement: Supplementary file 1 [file DataSheet3.ZIP › MRP1.tif]

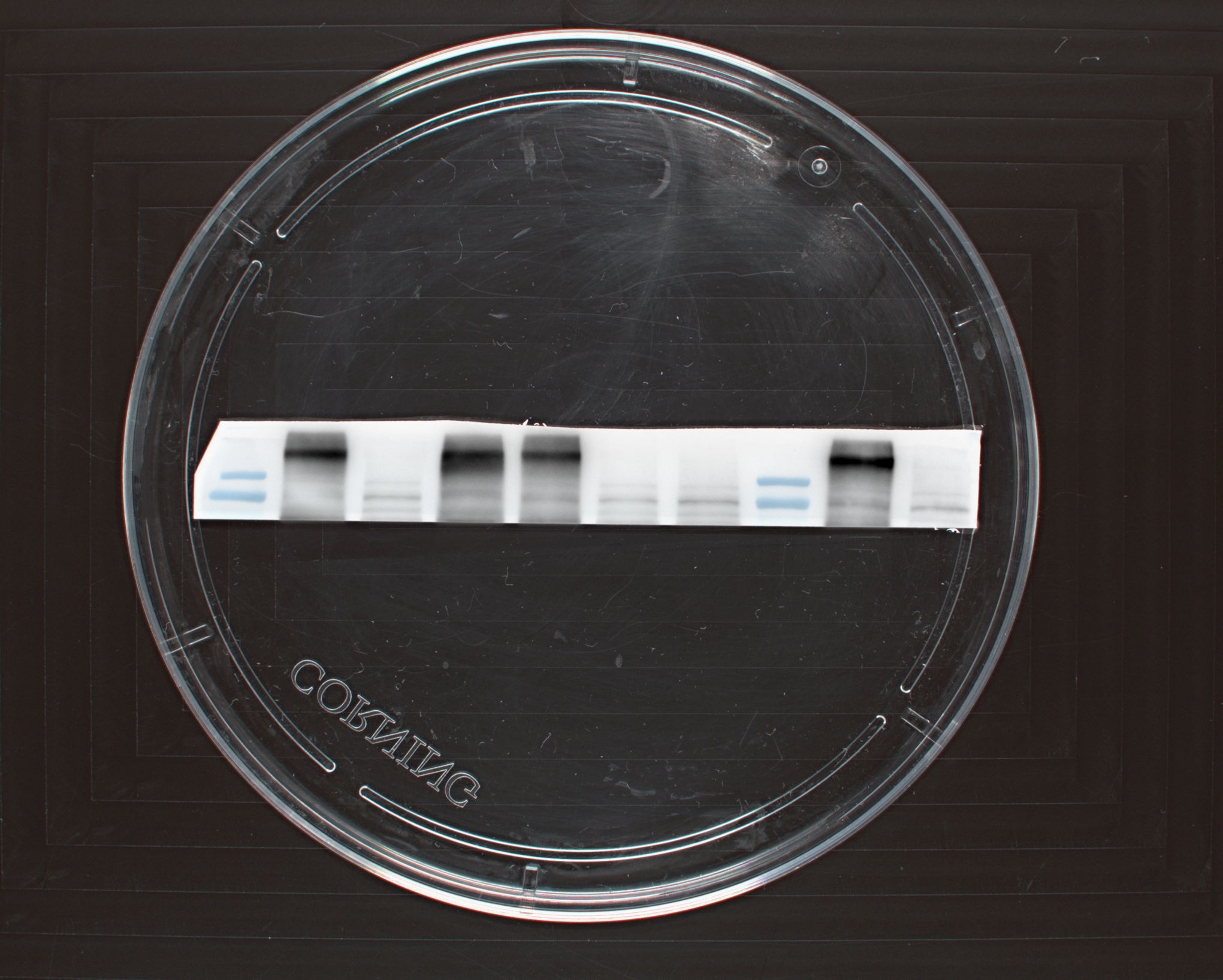

Supplement: Supplementary file 1 [file DataSheet3.ZIP › P-GP.tif]

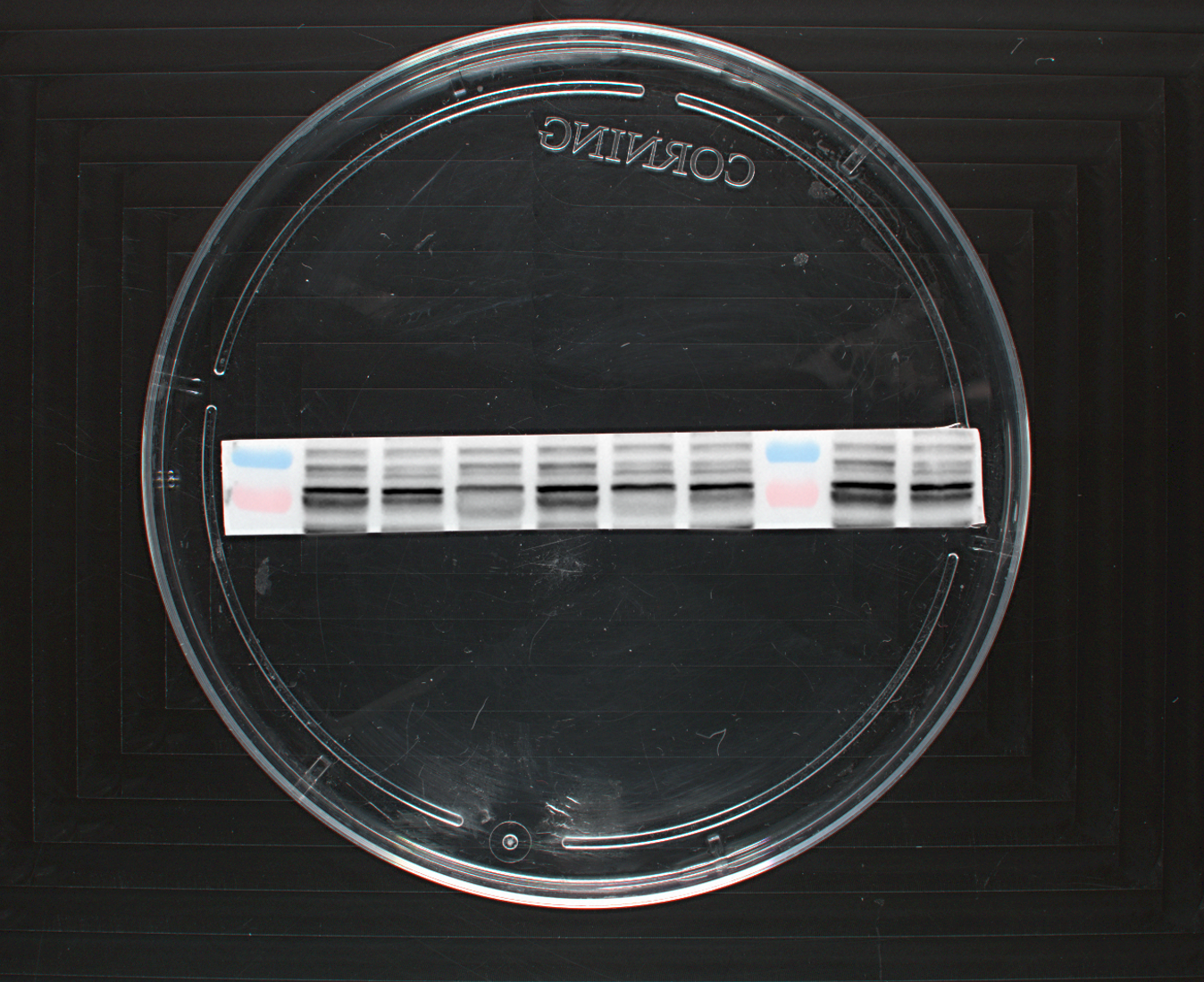

Supplement: Supplementary file 1 [file DataSheet3.ZIP › BCRP.tif]

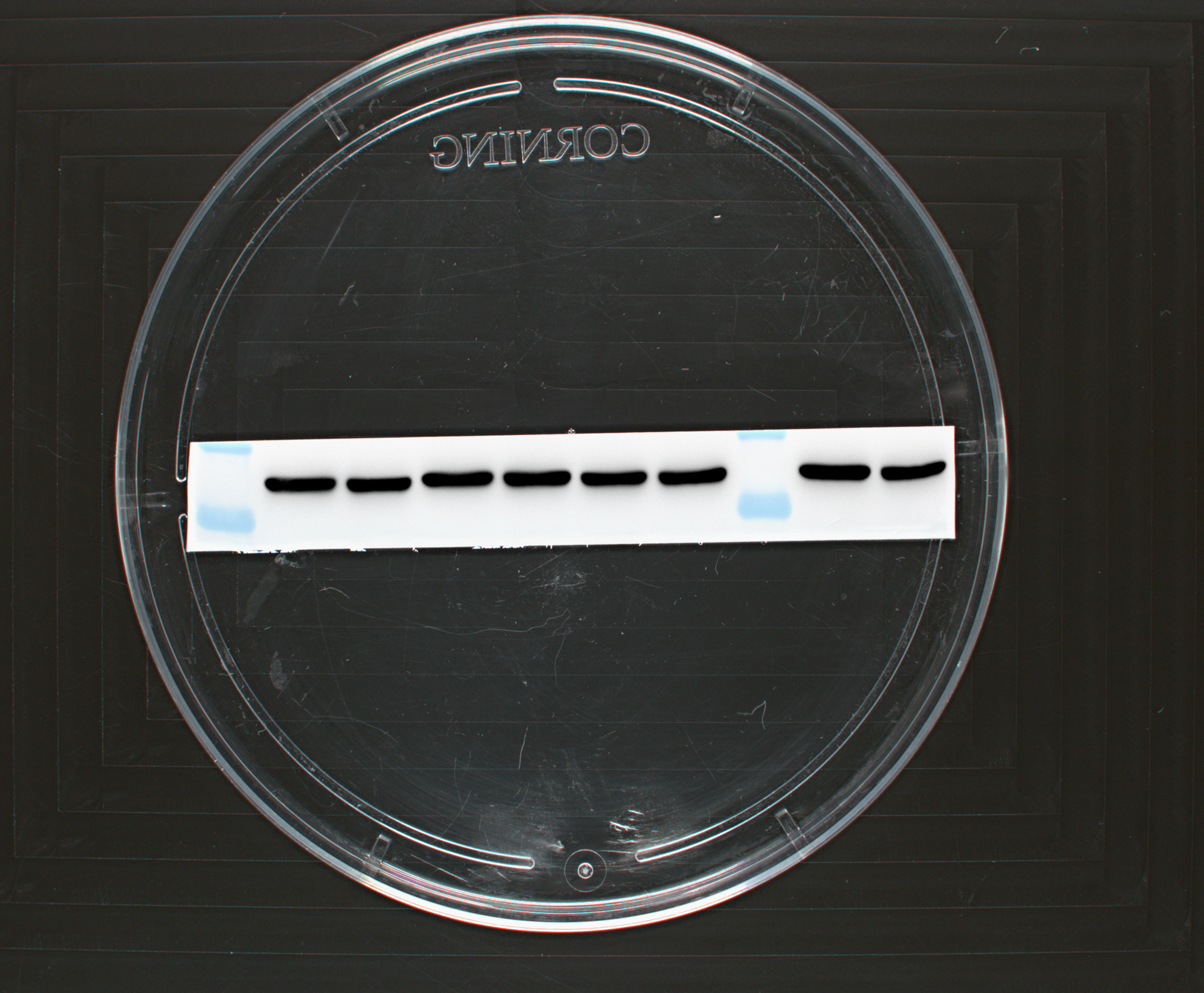

Supplement: Supplementary file 1 [file DataSheet3.ZIP › GAPDH.tif]

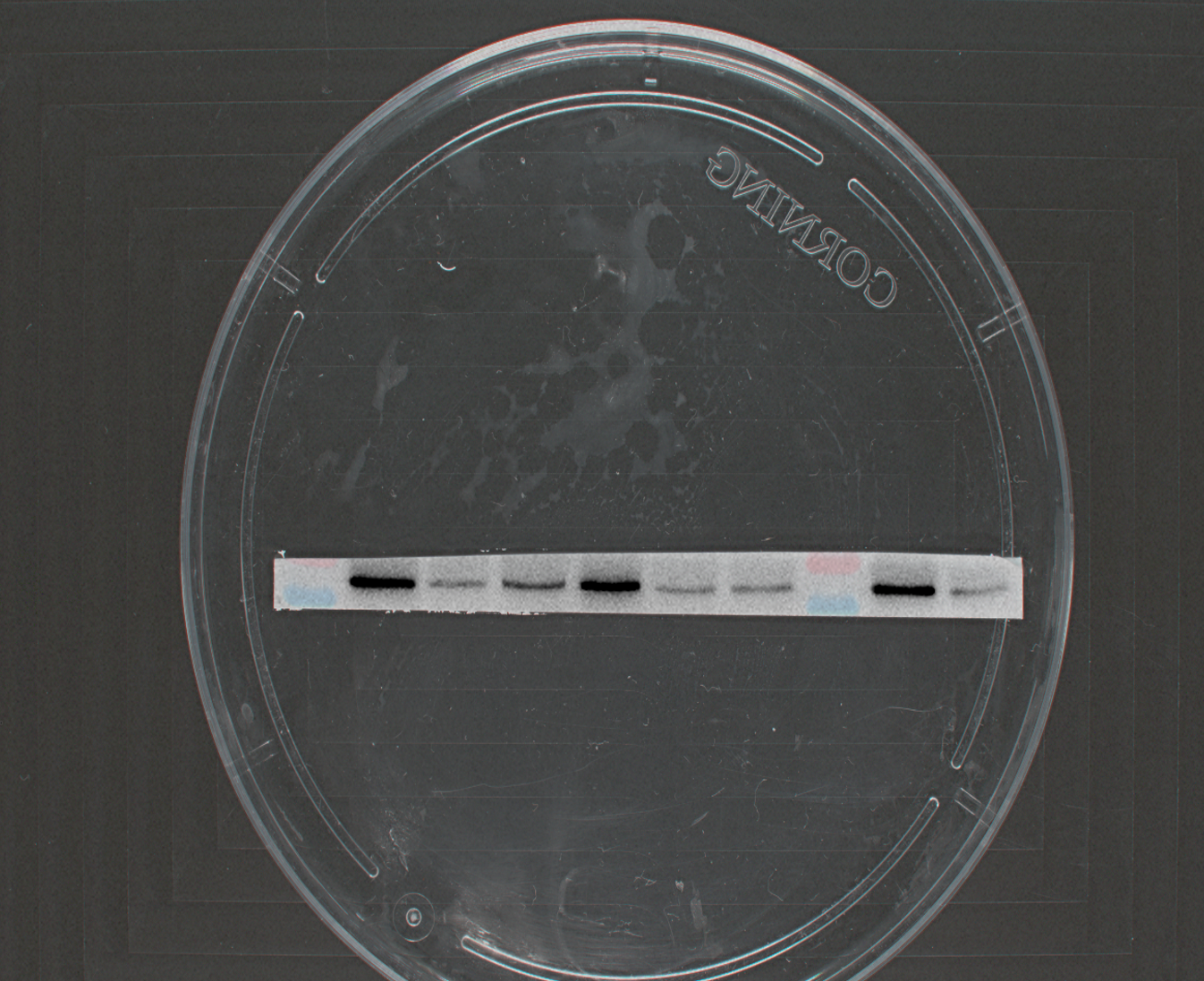

Supplement: Supplementary file 2 [file DataSheet4.ZIP › 2C-P62.tif]

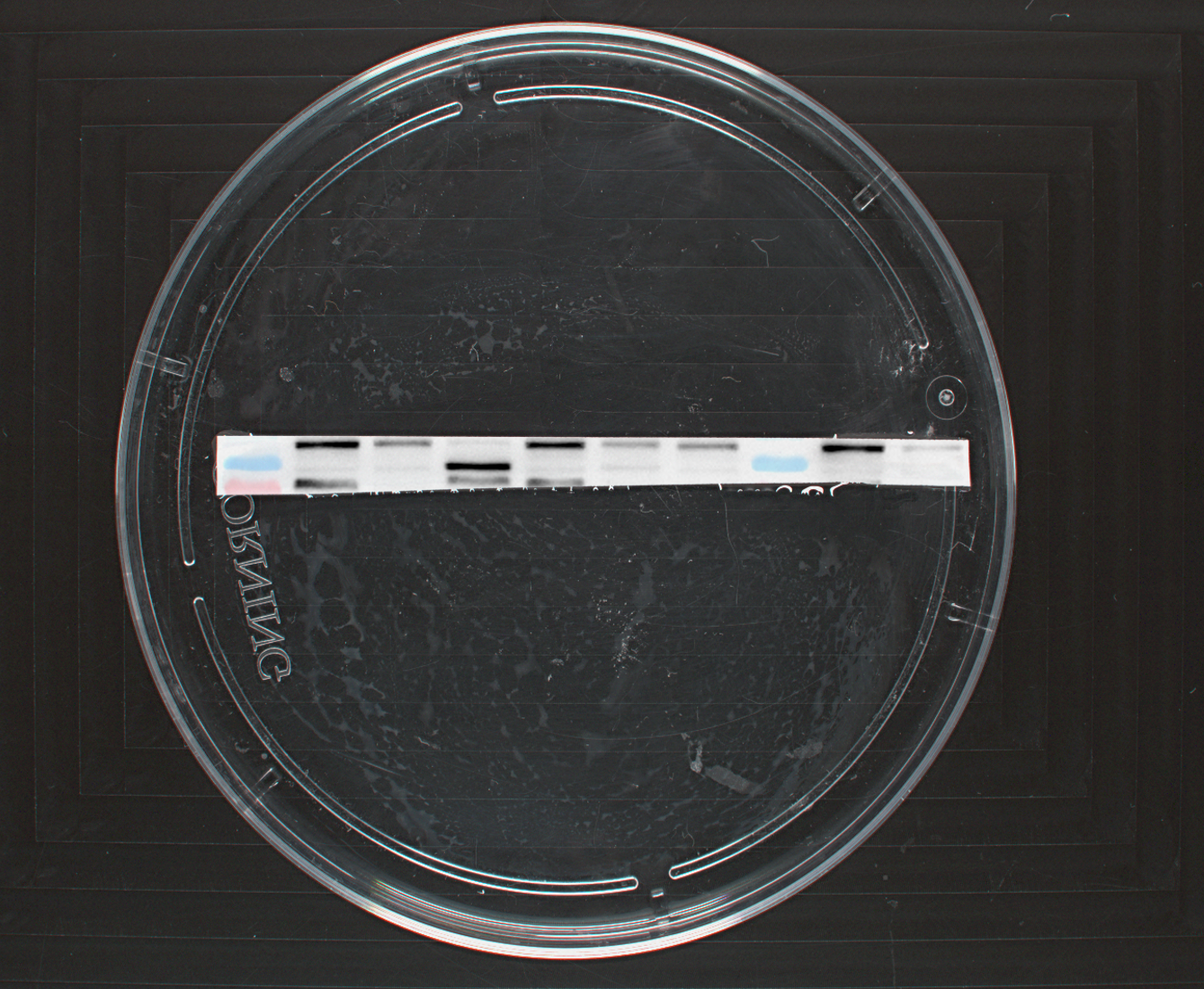

Supplement: Supplementary file 2 [file DataSheet4.ZIP › 2C-PARP.tif]

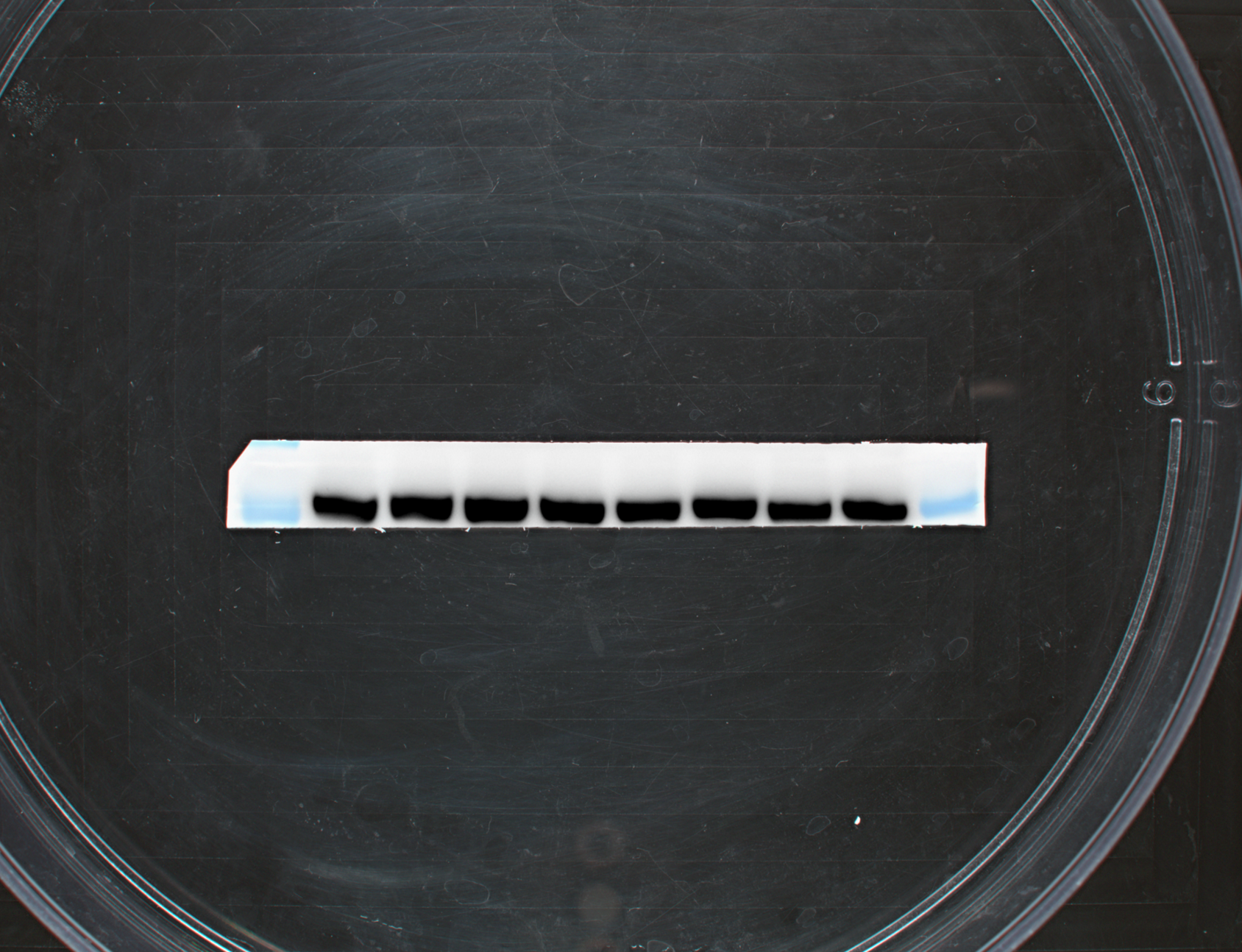

Supplement: Supplementary file 2 [file DataSheet4.ZIP › 2E-GAPDH-1.tif]

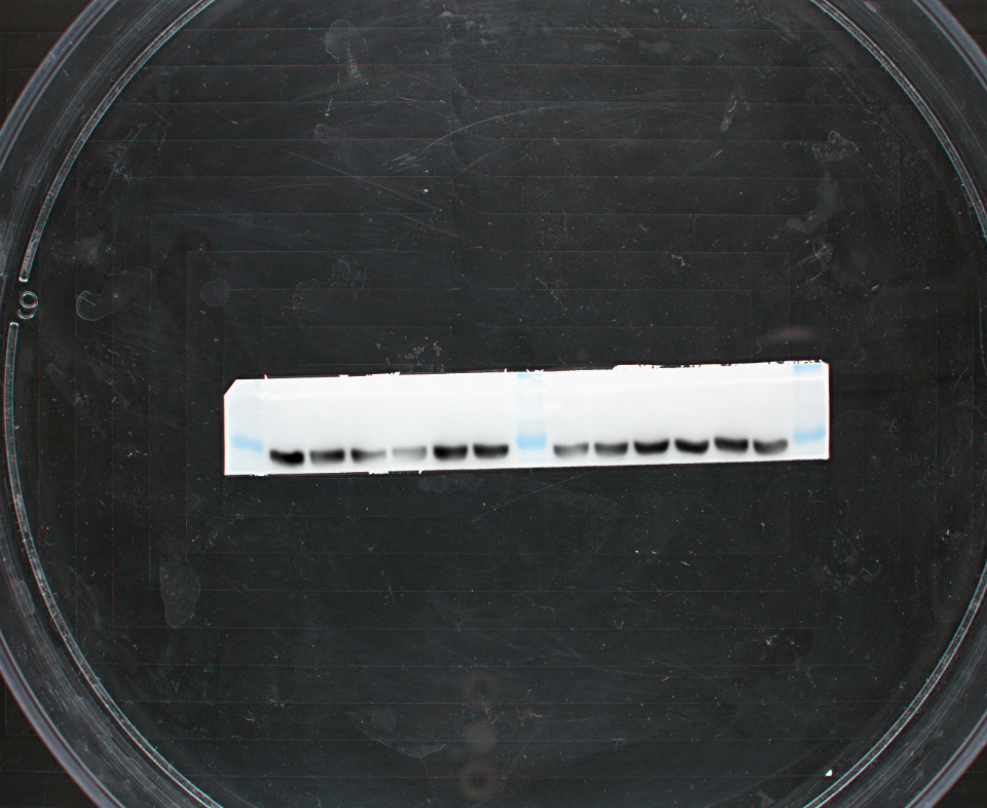

Supplement: Supplementary file 2 [file DataSheet4.ZIP › 2E-GAPDH-2.tif]

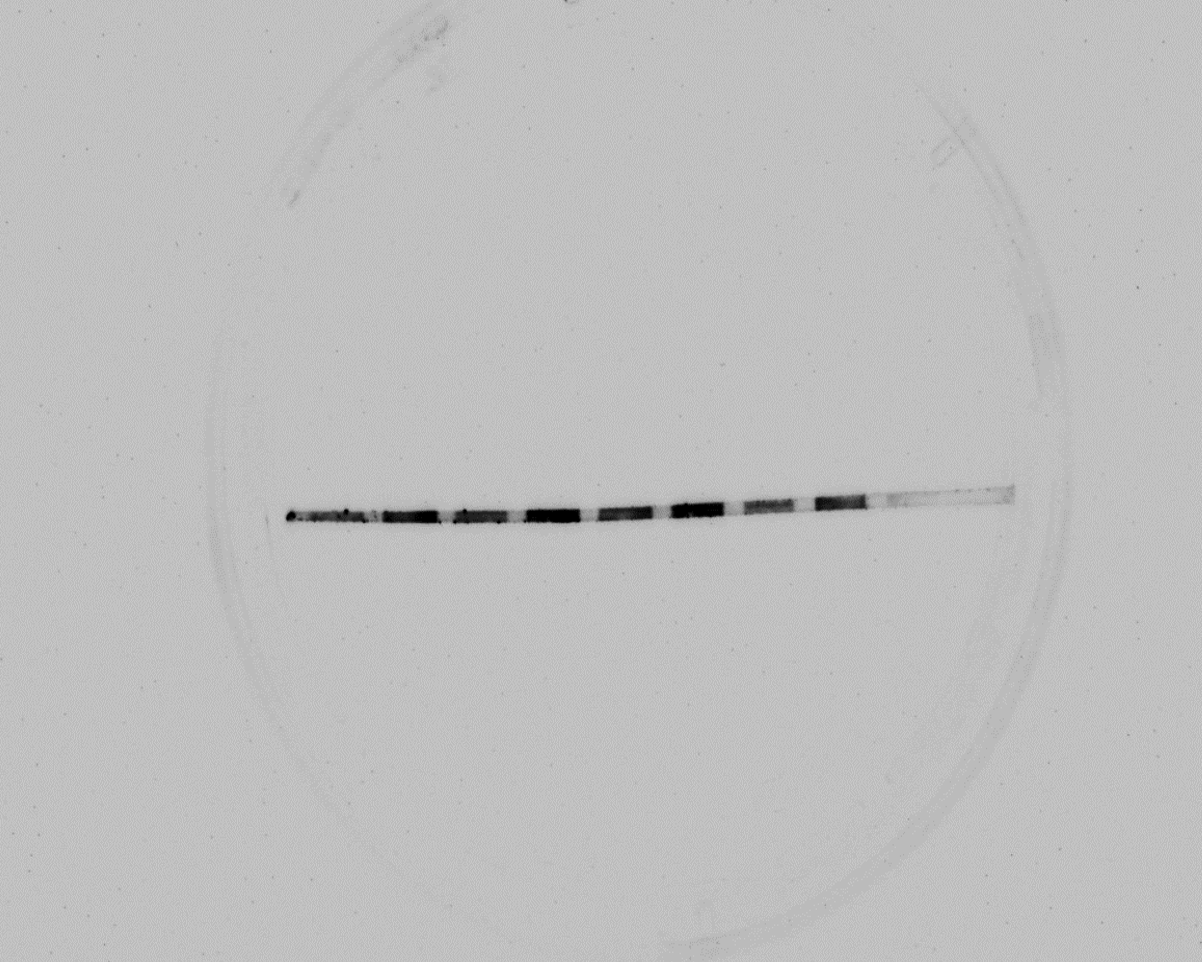

Supplement: Supplementary file 2 [file DataSheet4.ZIP › 2E-MRP1-1.tif]

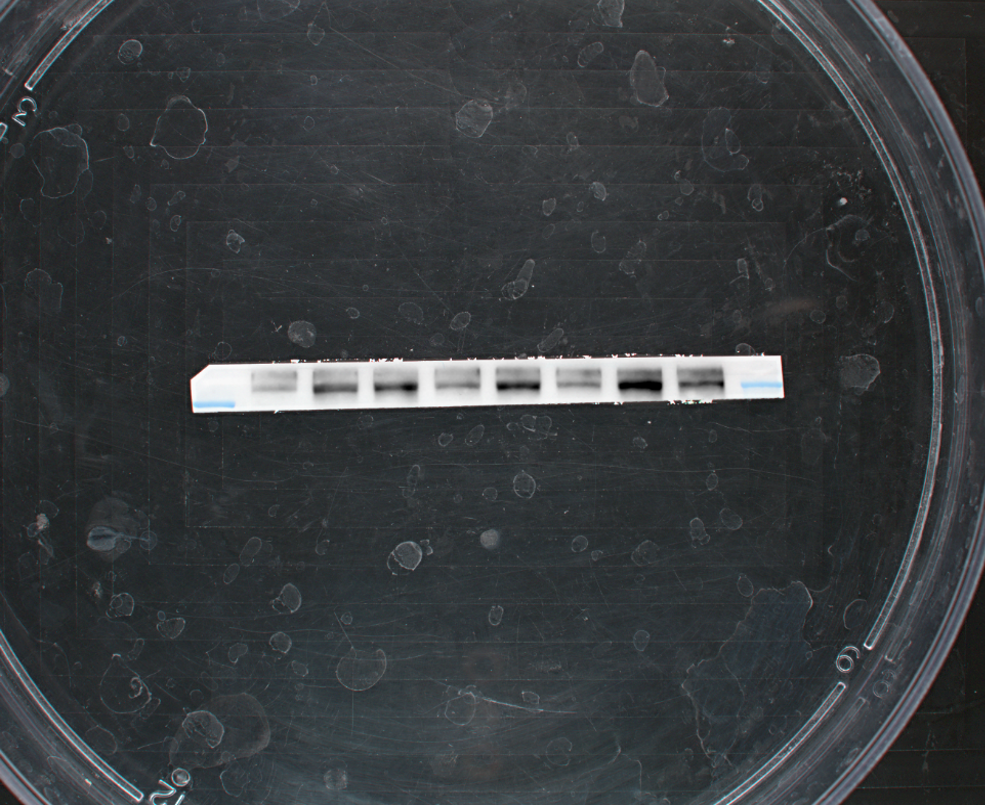

Supplement: Supplementary file 2 [file DataSheet4.ZIP › 2E-MRP1-2.tif]

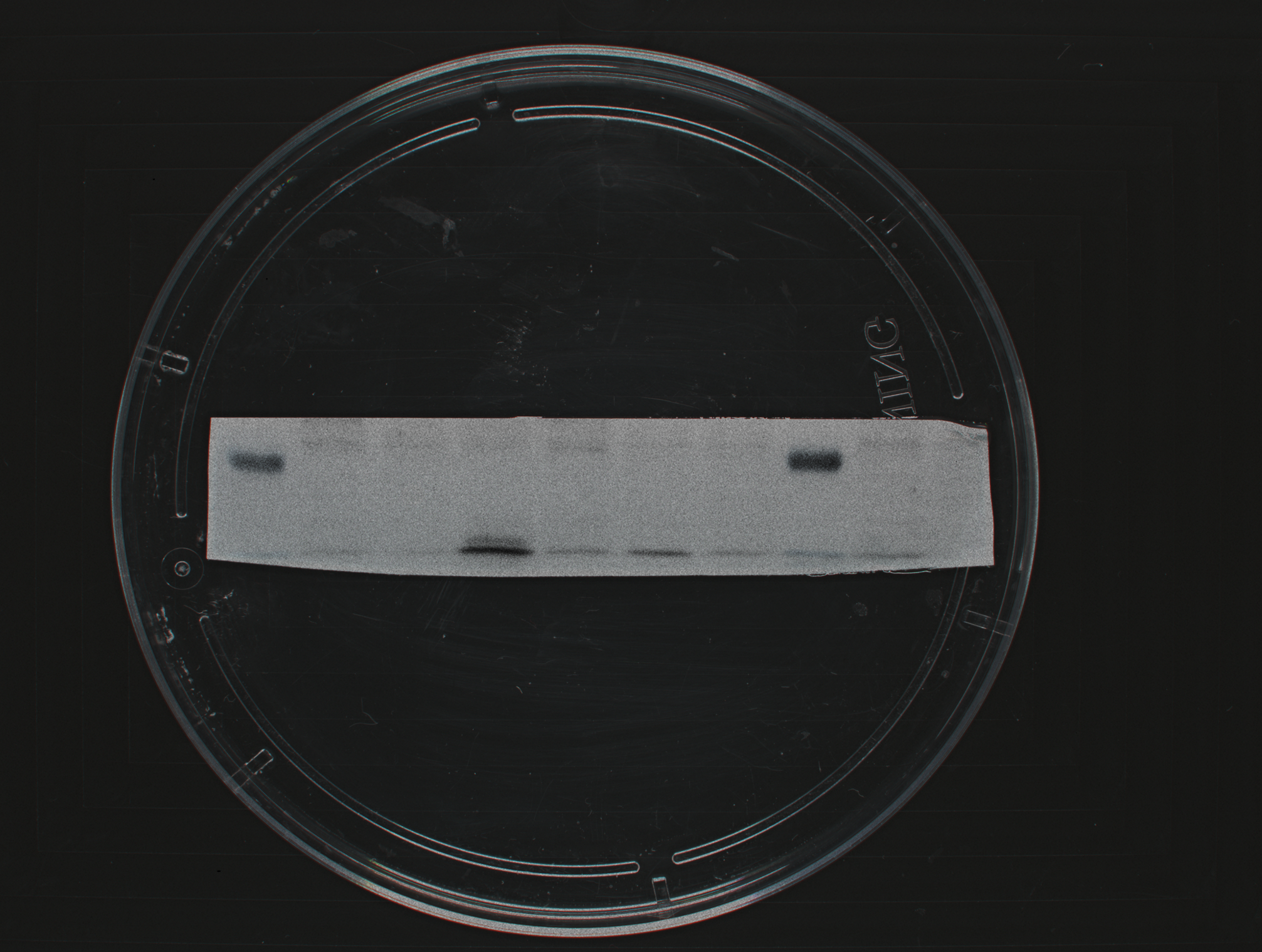

Supplement: Supplementary file 2 [file DataSheet4.ZIP › 2C-C-CAS3.tif]

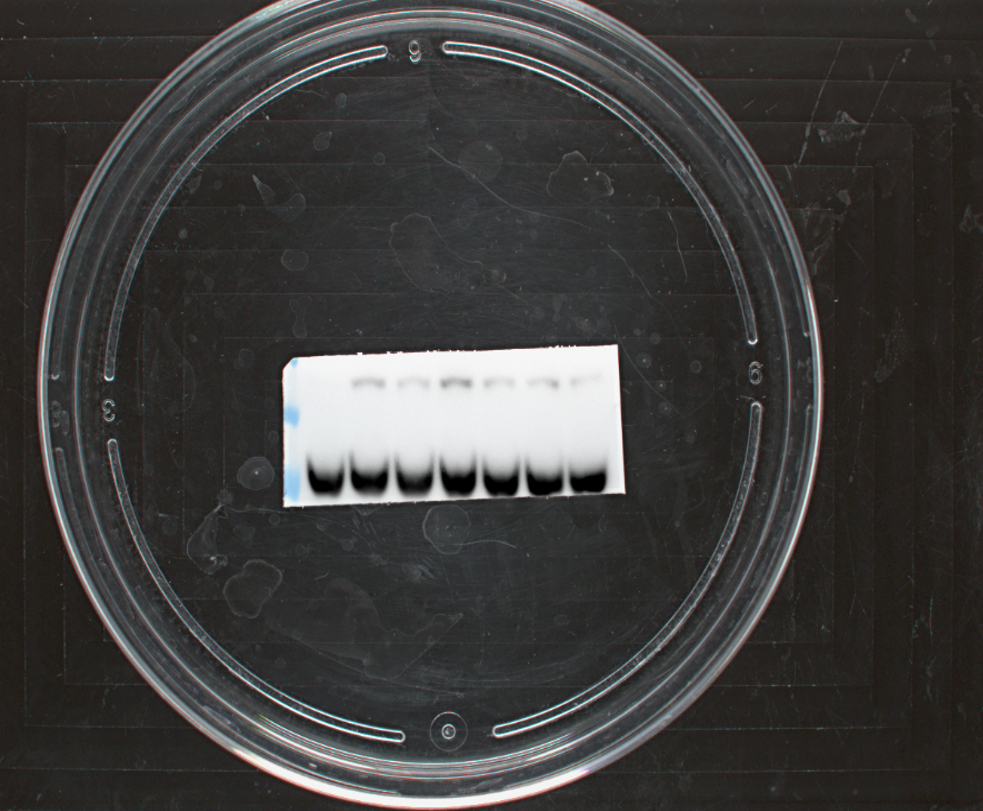

Supplement: Supplementary file 2 [file DataSheet4.ZIP › 2C-GAPDH.tif]

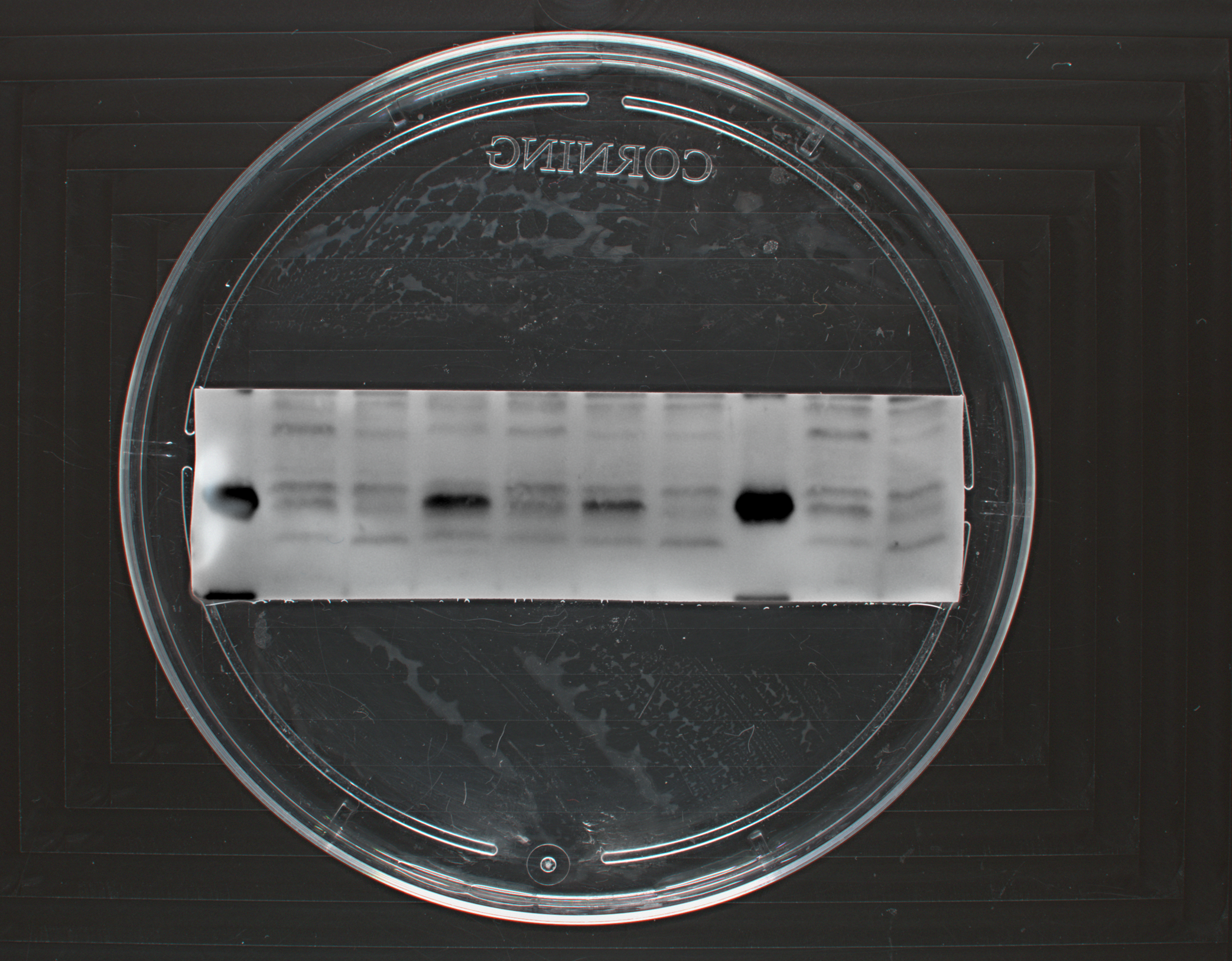

Supplement: Supplementary file 2 [file DataSheet4.ZIP › 2C-LC3.tif]

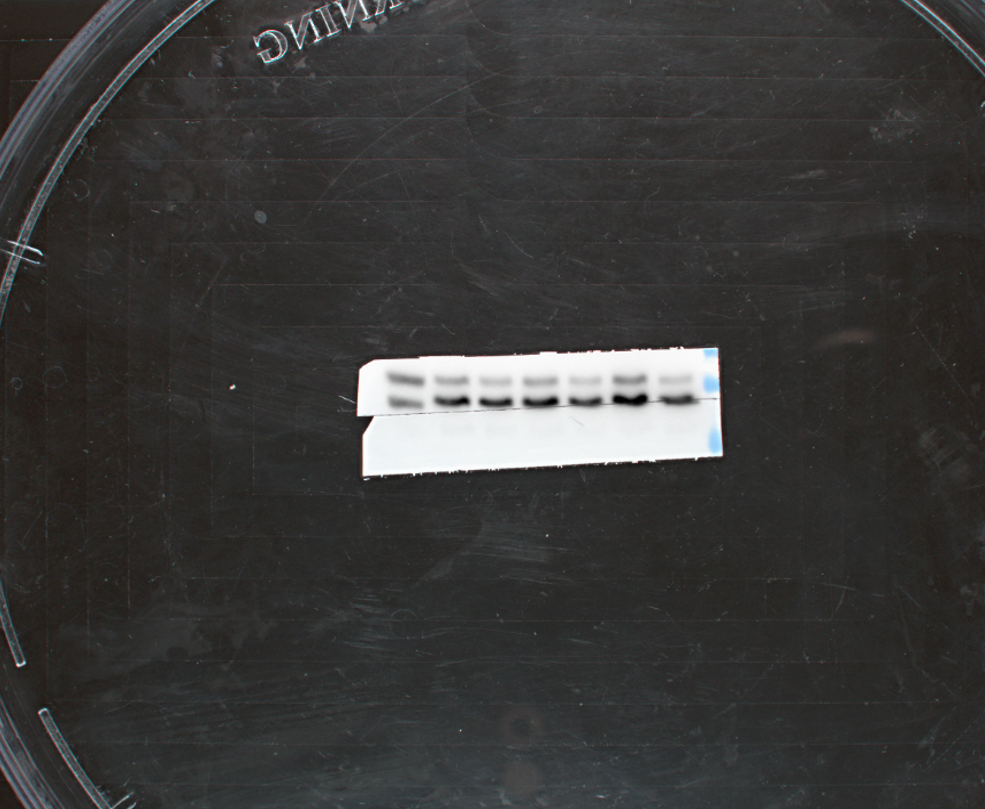

Supplement: Supplementary file 4 [file DataSheet6.ZIP › 4A-ERK12.tif]

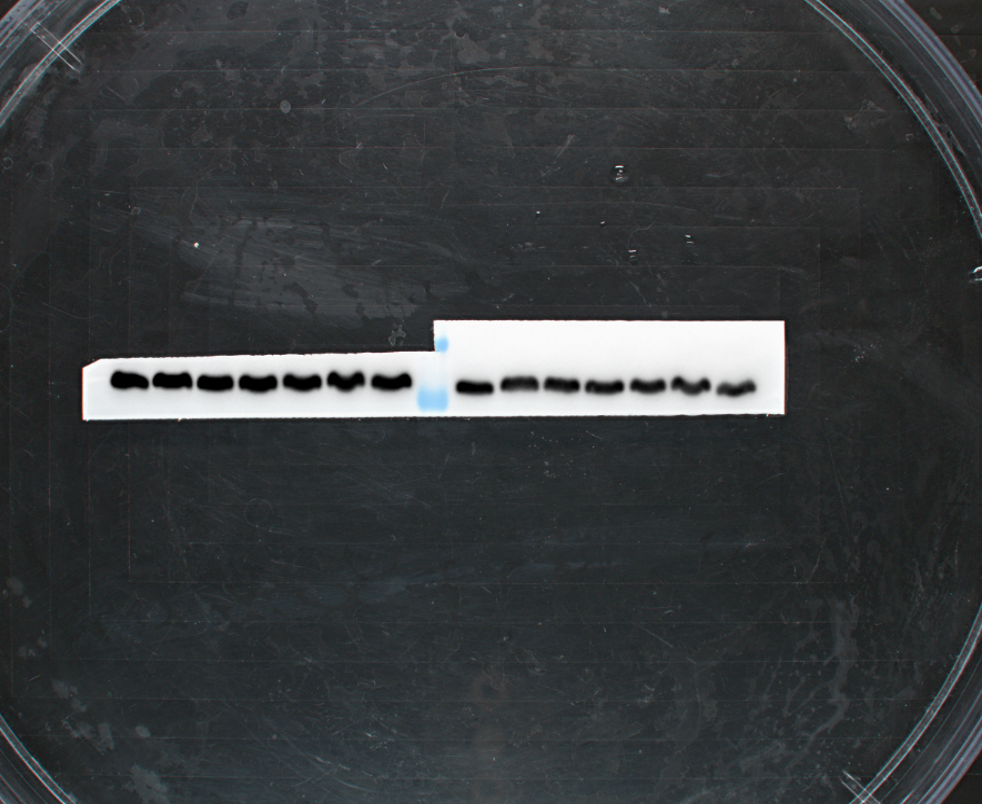

Supplement: Supplementary file 4 [file DataSheet6.ZIP › 4A-GAPDH.tif]

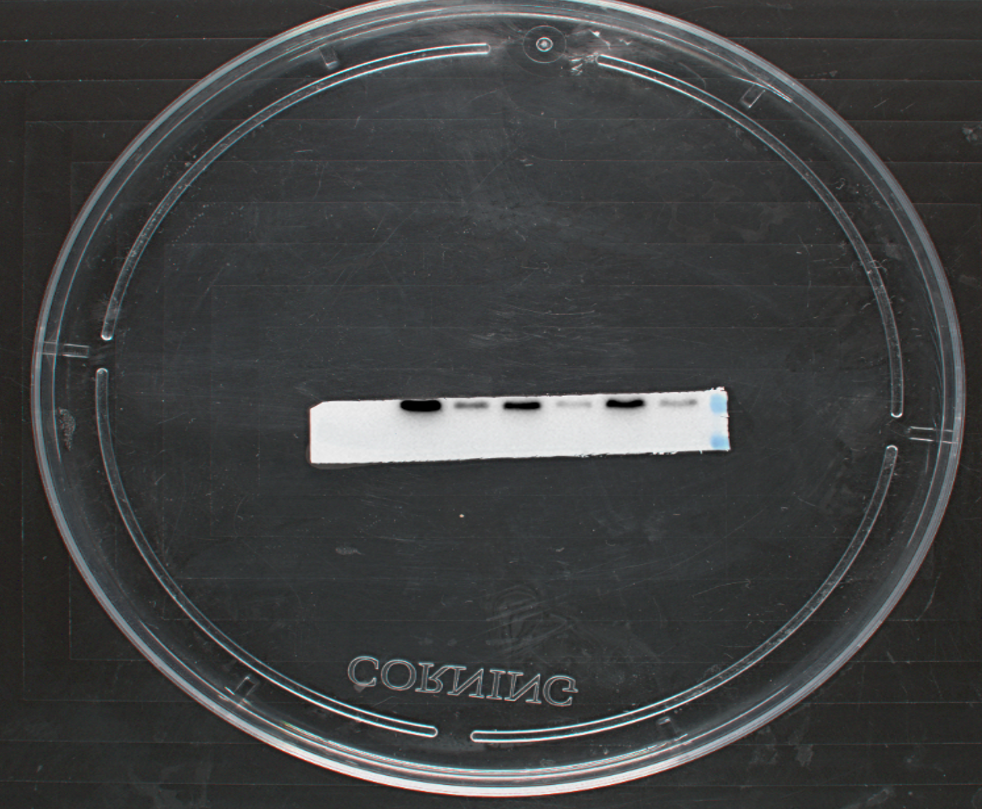

Supplement: Supplementary file 4 [file DataSheet6.ZIP › 4A-P53.tif]

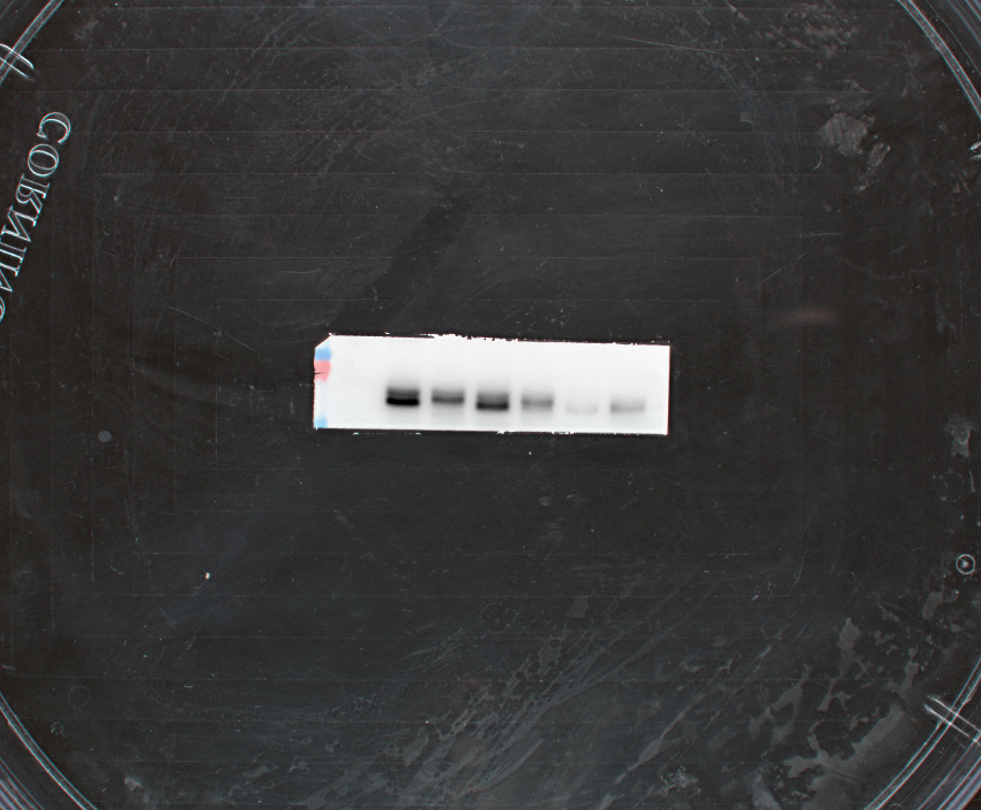

Supplement: Supplementary file 4 [file DataSheet6.ZIP › 4A-P-AKT.tif]

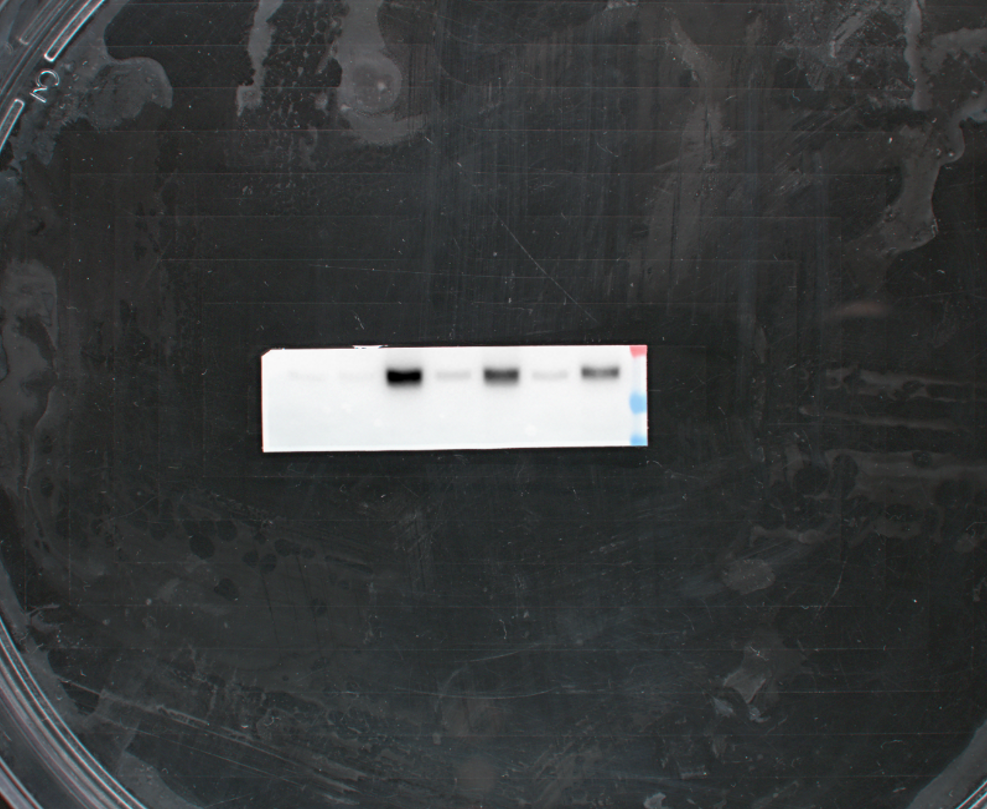

Supplement: Supplementary file 4 [file DataSheet6.ZIP › 4A-P-AMPK.tif]

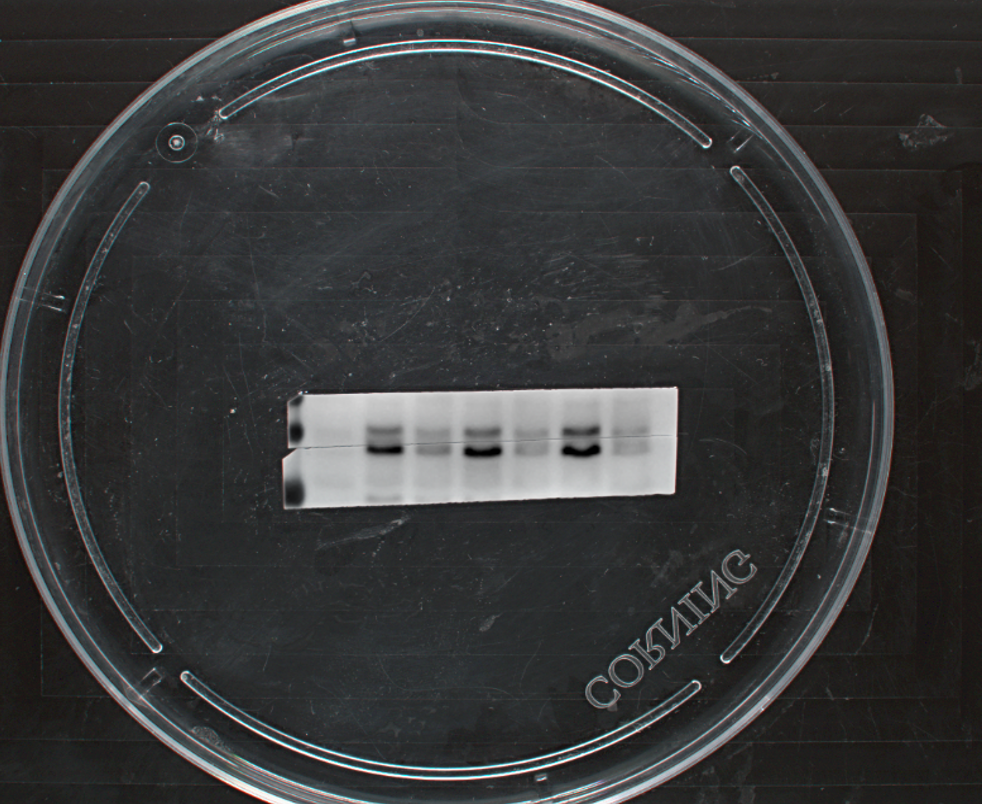

Supplement: Supplementary file 4 [file DataSheet6.ZIP › 4A-P-ERK12.tif]

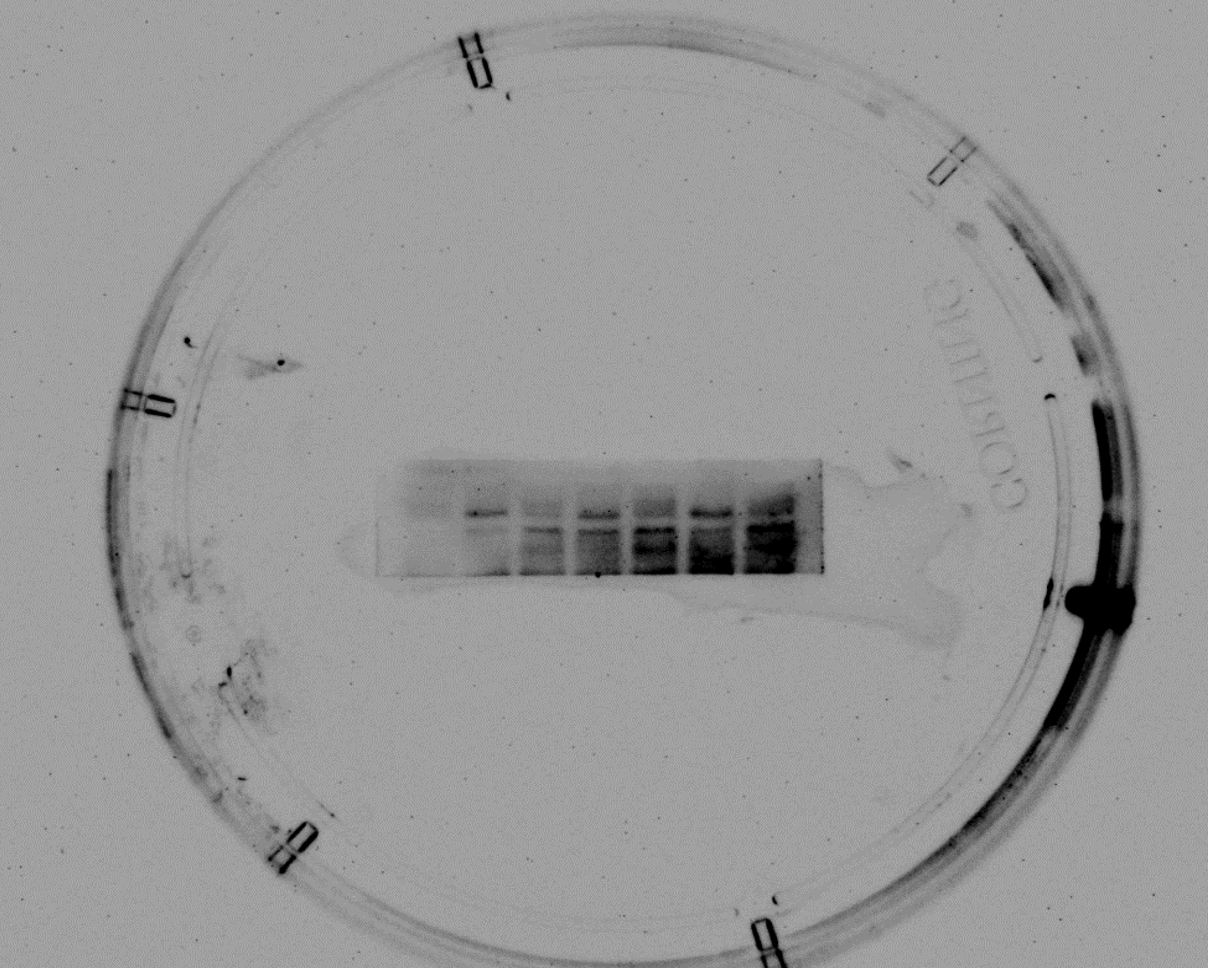

Supplement: Supplementary file 4 [file DataSheet6.ZIP › 4A-P-P53.tif]

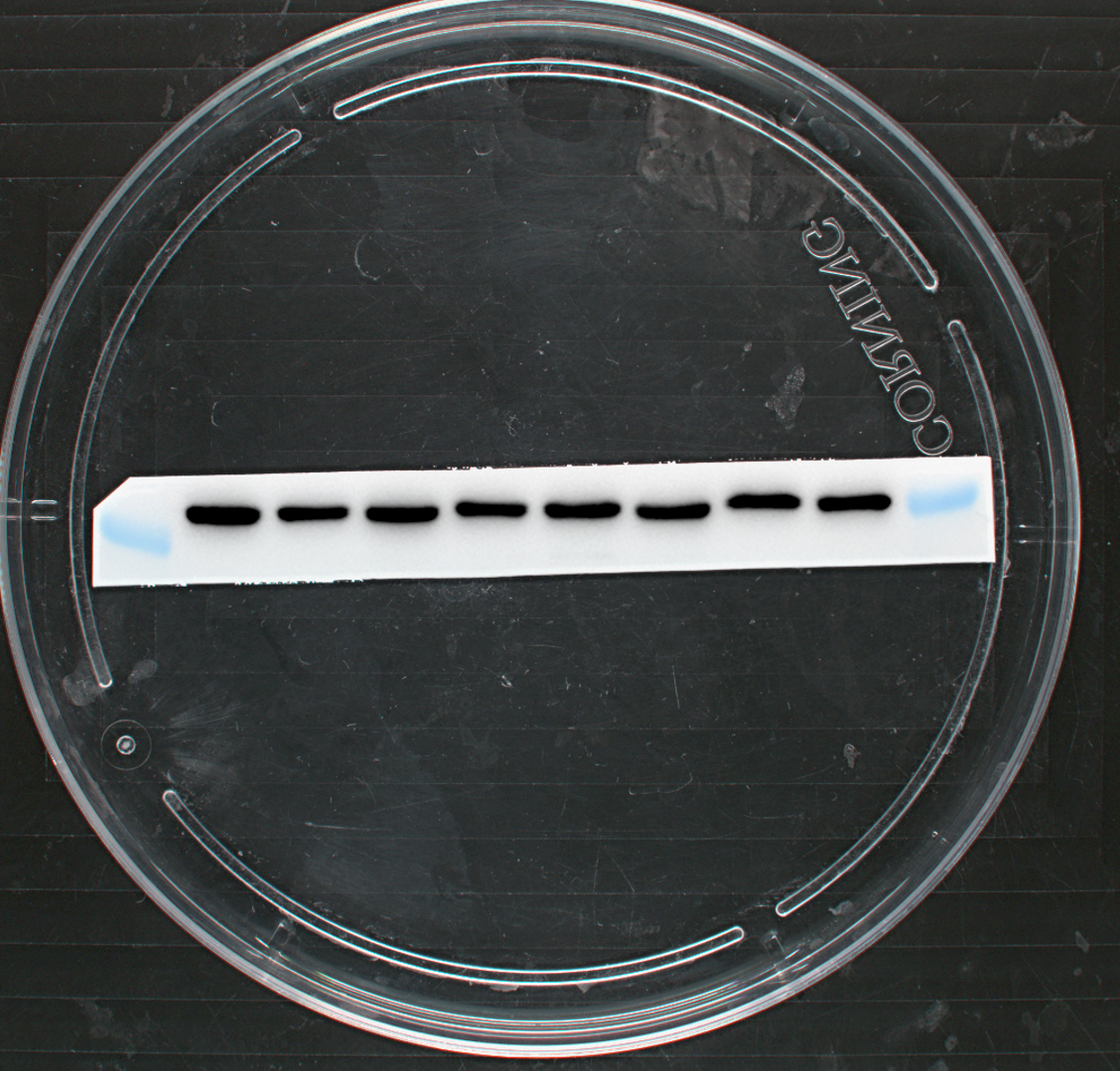

Supplement: Supplementary file 4 [file DataSheet6.ZIP › 4C-GAPDH-1.tif]

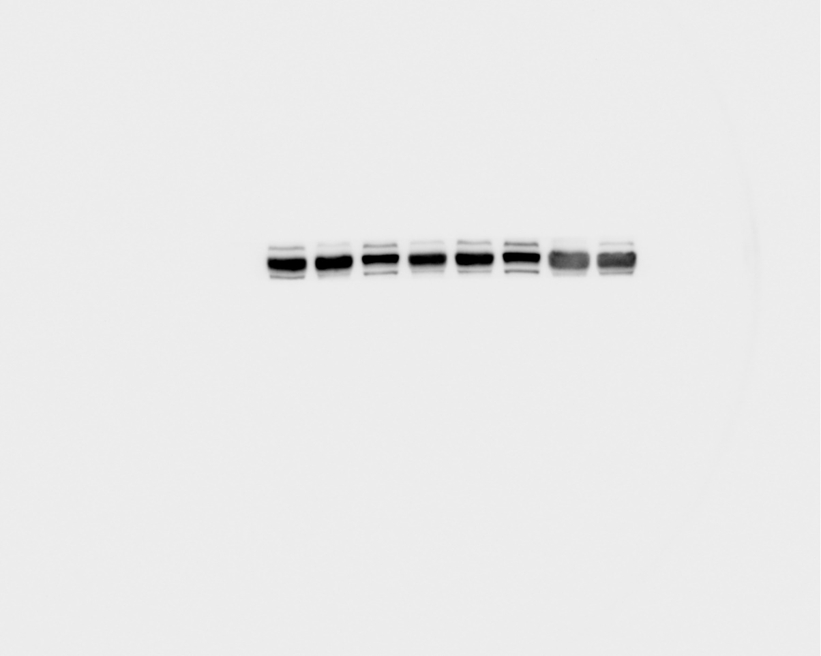

Supplement: Supplementary file 4 [file DataSheet6.ZIP › 4C-GAPDH-2.tif]

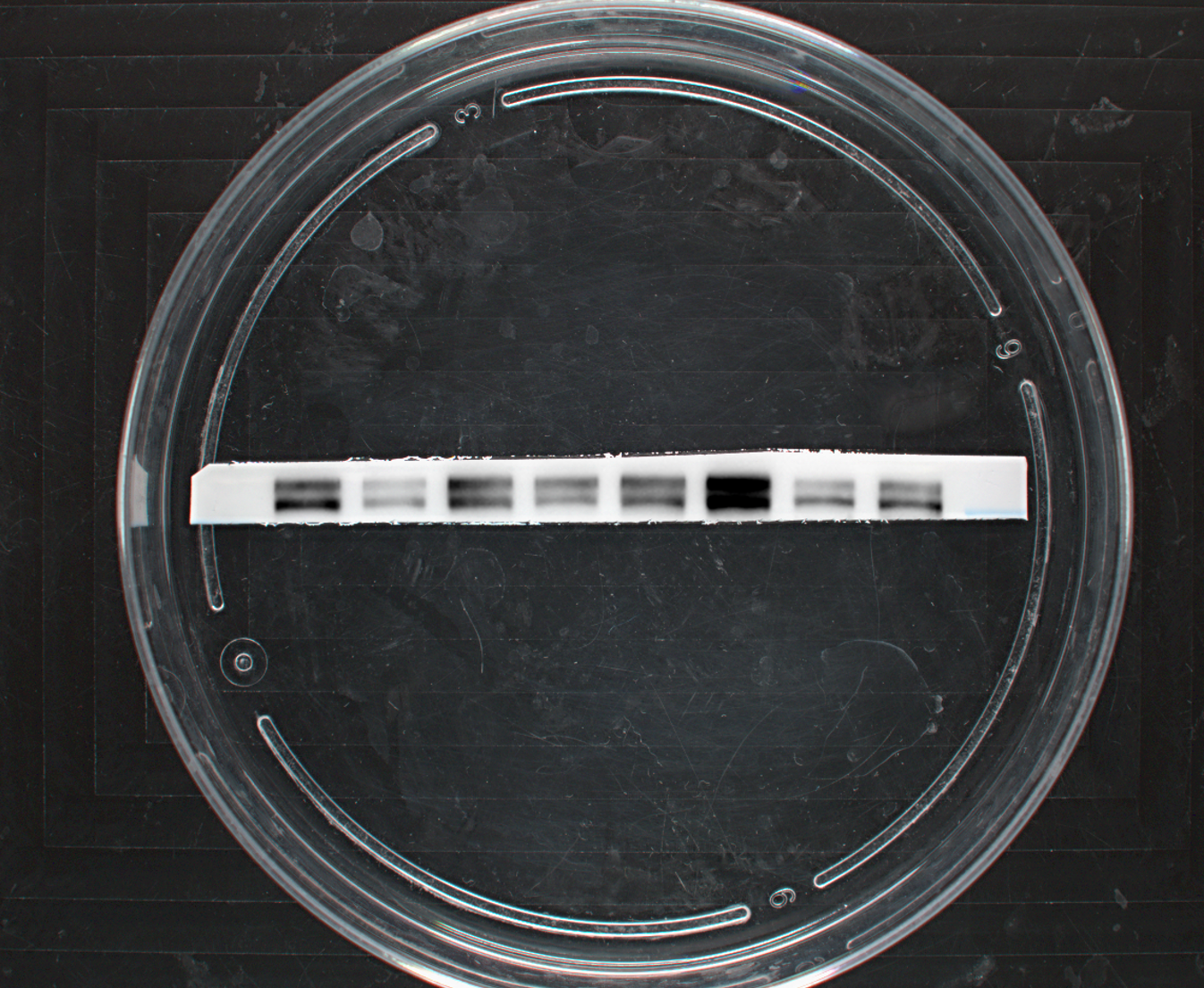

Supplement: Supplementary file 4 [file DataSheet6.ZIP › 4C-MRP1-1.tif]

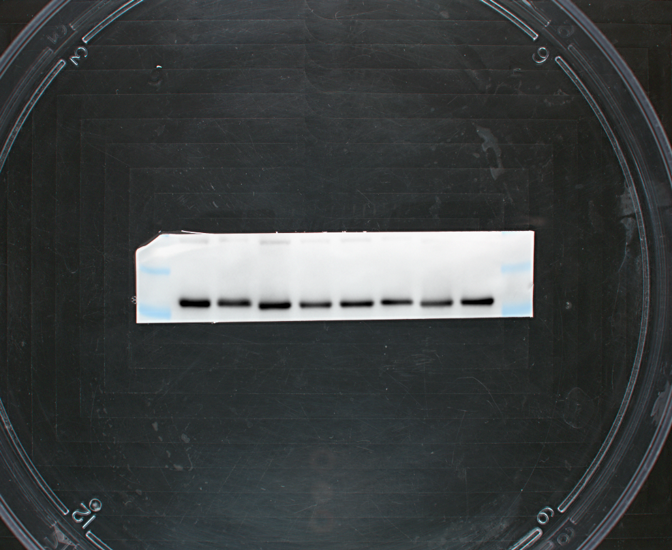

Supplement: Supplementary file 4 [file DataSheet6.ZIP › 4C-MRP1-2.tif]

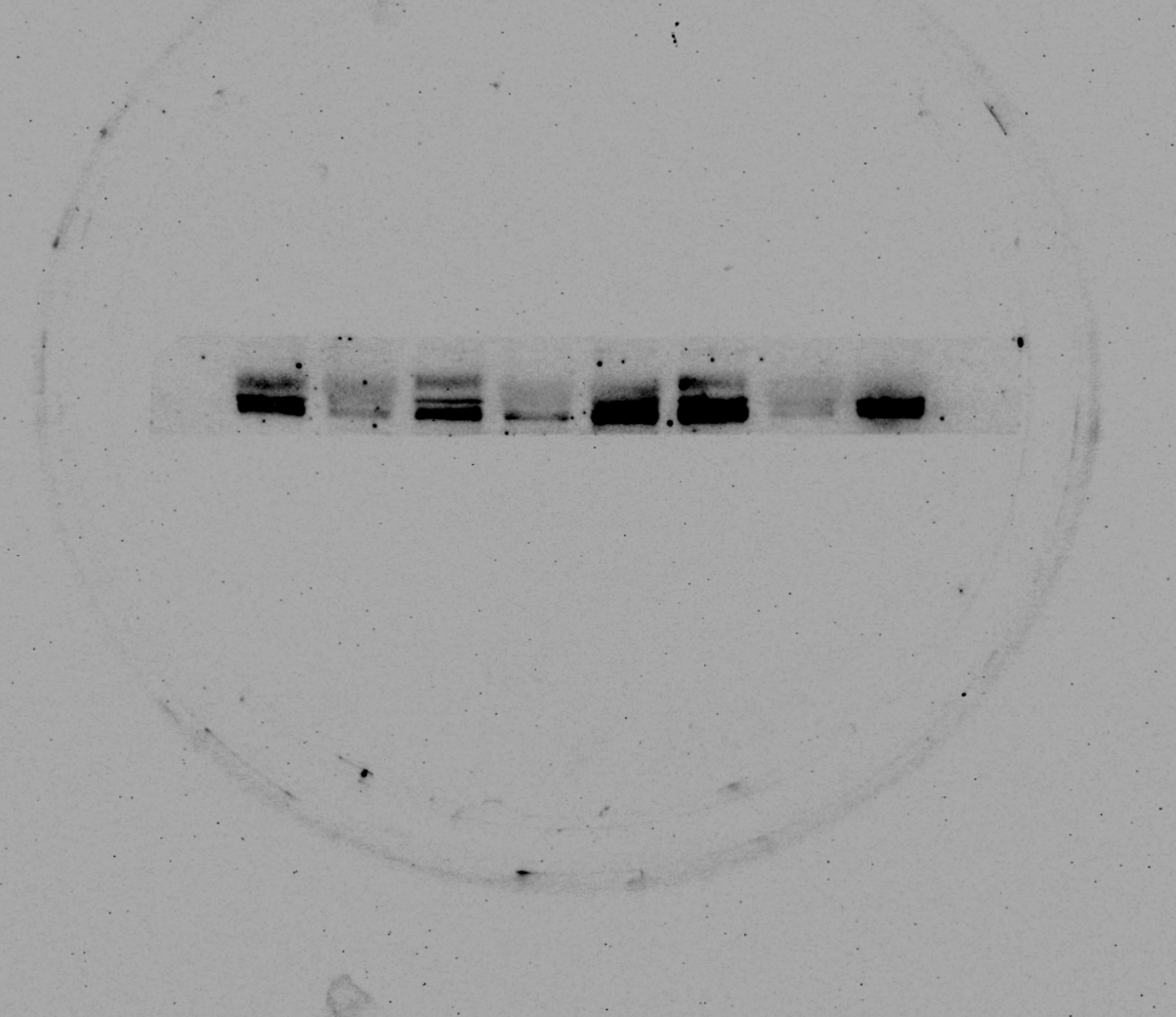

Supplement: Supplementary file 4 [file DataSheet6.ZIP › 4C-P-MTOR-1.tif]

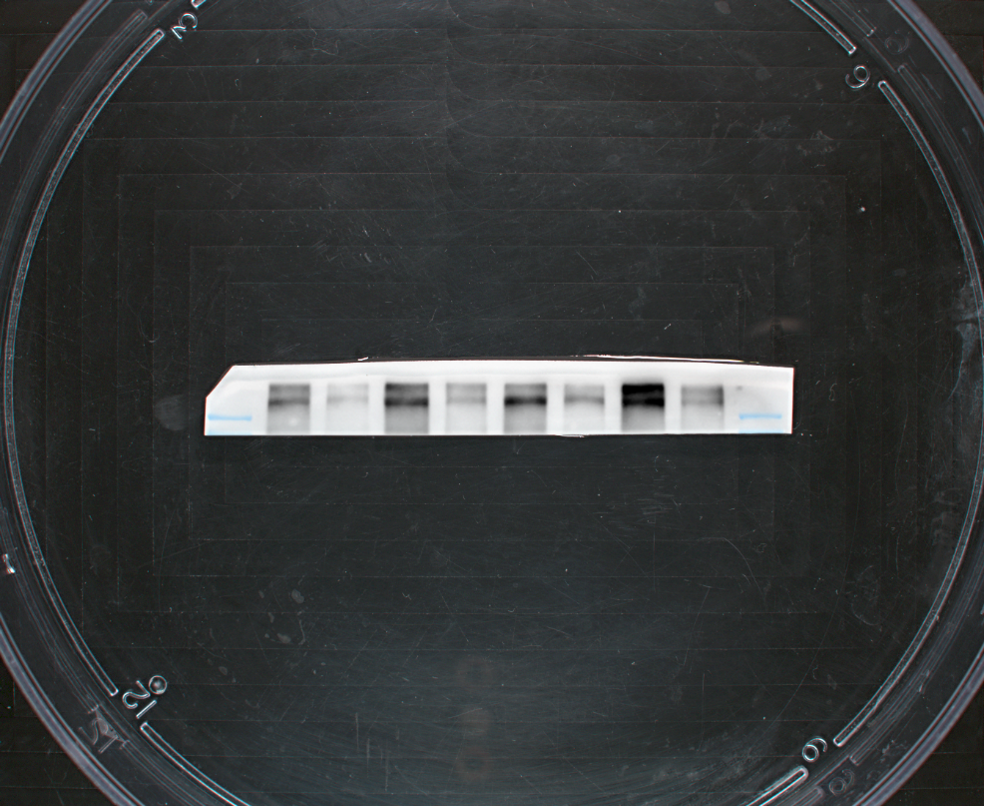

Supplement: Supplementary file 4 [file DataSheet6.ZIP › 4C-P-MTOR-2.tif]

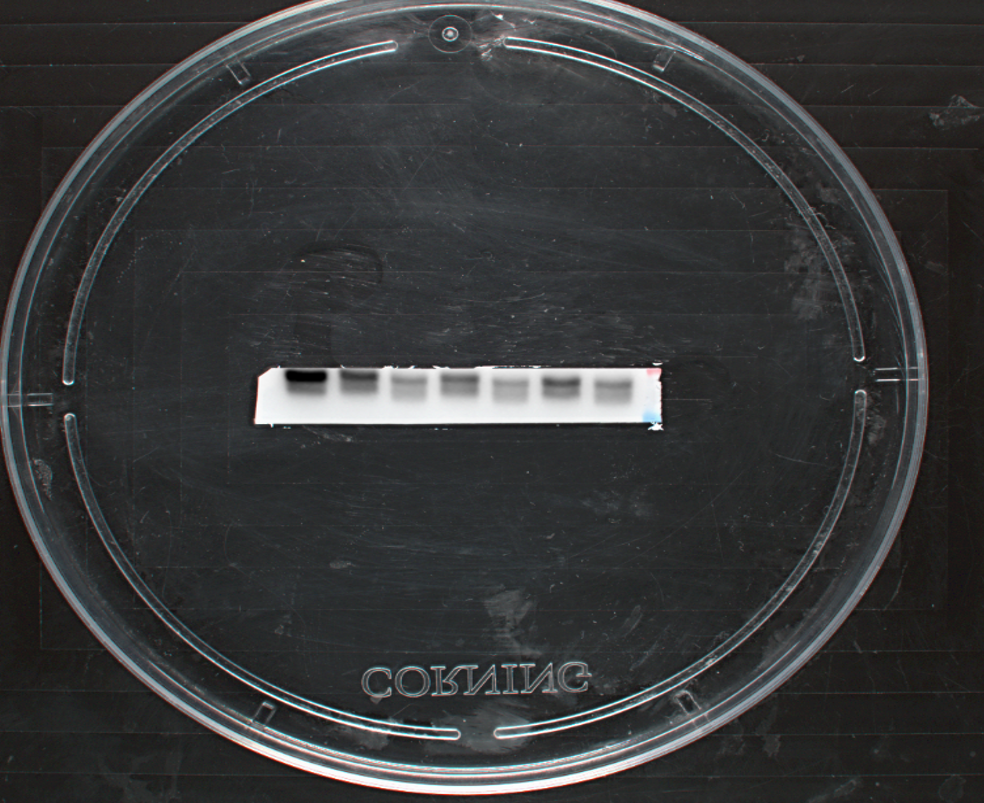

Supplement: Supplementary file 4 [file DataSheet6.ZIP › 4A-AKT.tif]

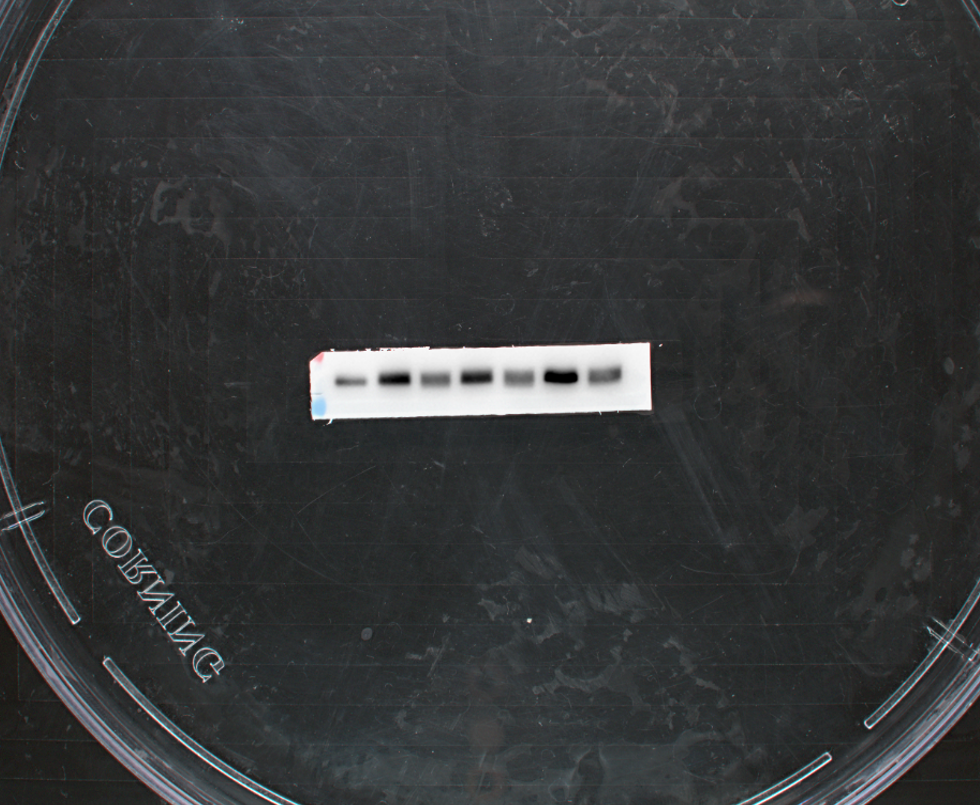

Supplement: Supplementary file 4 [file DataSheet6.ZIP › 4A-AMPK.tif]

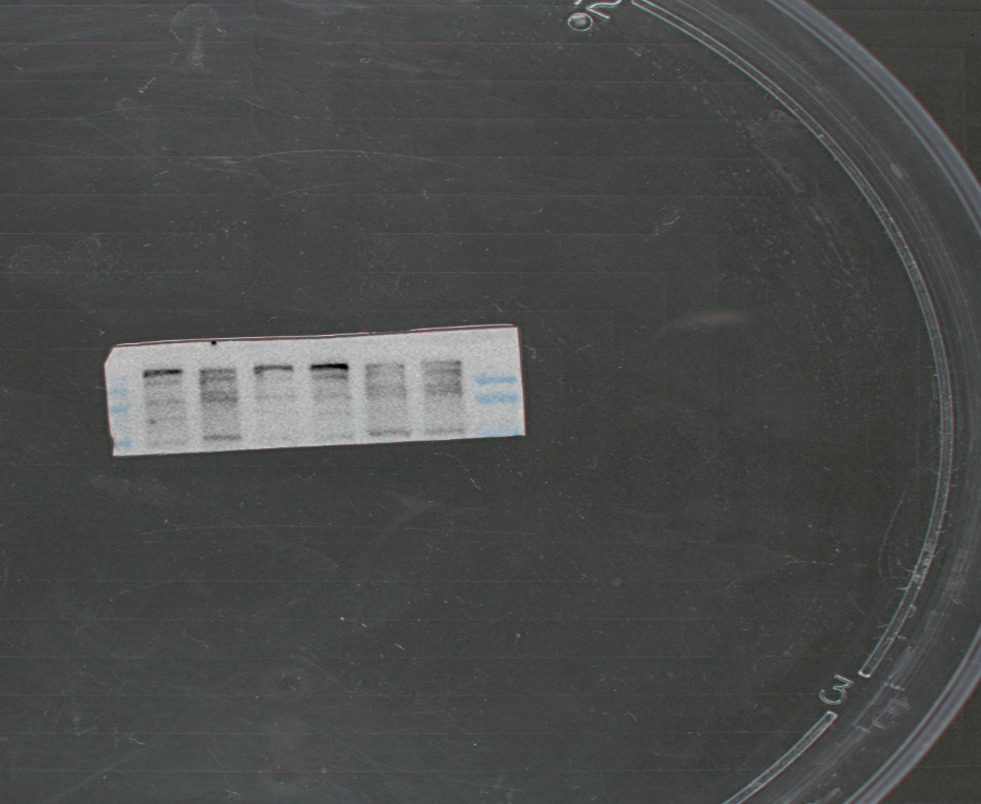

Supplement: Supplementary file 7 [file DataSheet5.ZIP › 3A-P-MTOR.tif]

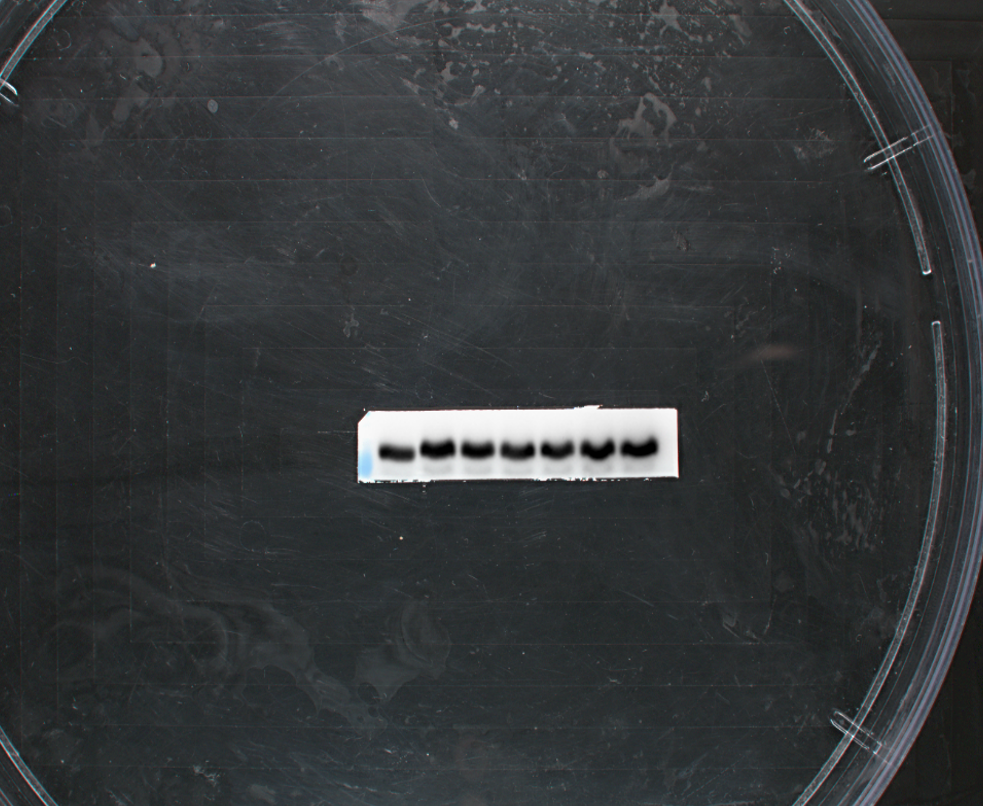

Supplement: Supplementary file 7 [file DataSheet5.ZIP › 3B-GAPDH.tif]

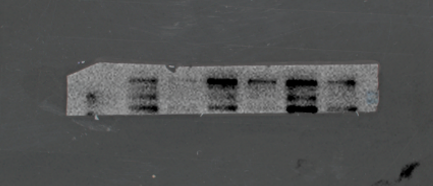

Supplement: Supplementary file 7 [file DataSheet5.ZIP › 3B-MTOR.tif]

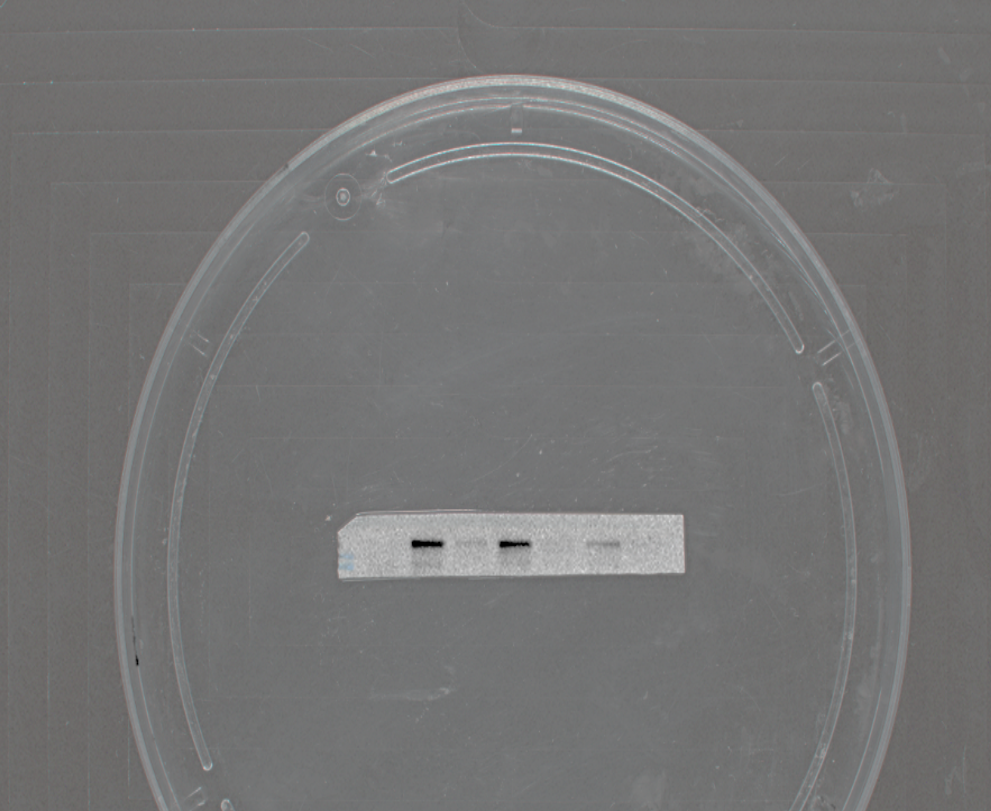

Supplement: Supplementary file 7 [file DataSheet5.ZIP › 3B-P-MTOR.tif]

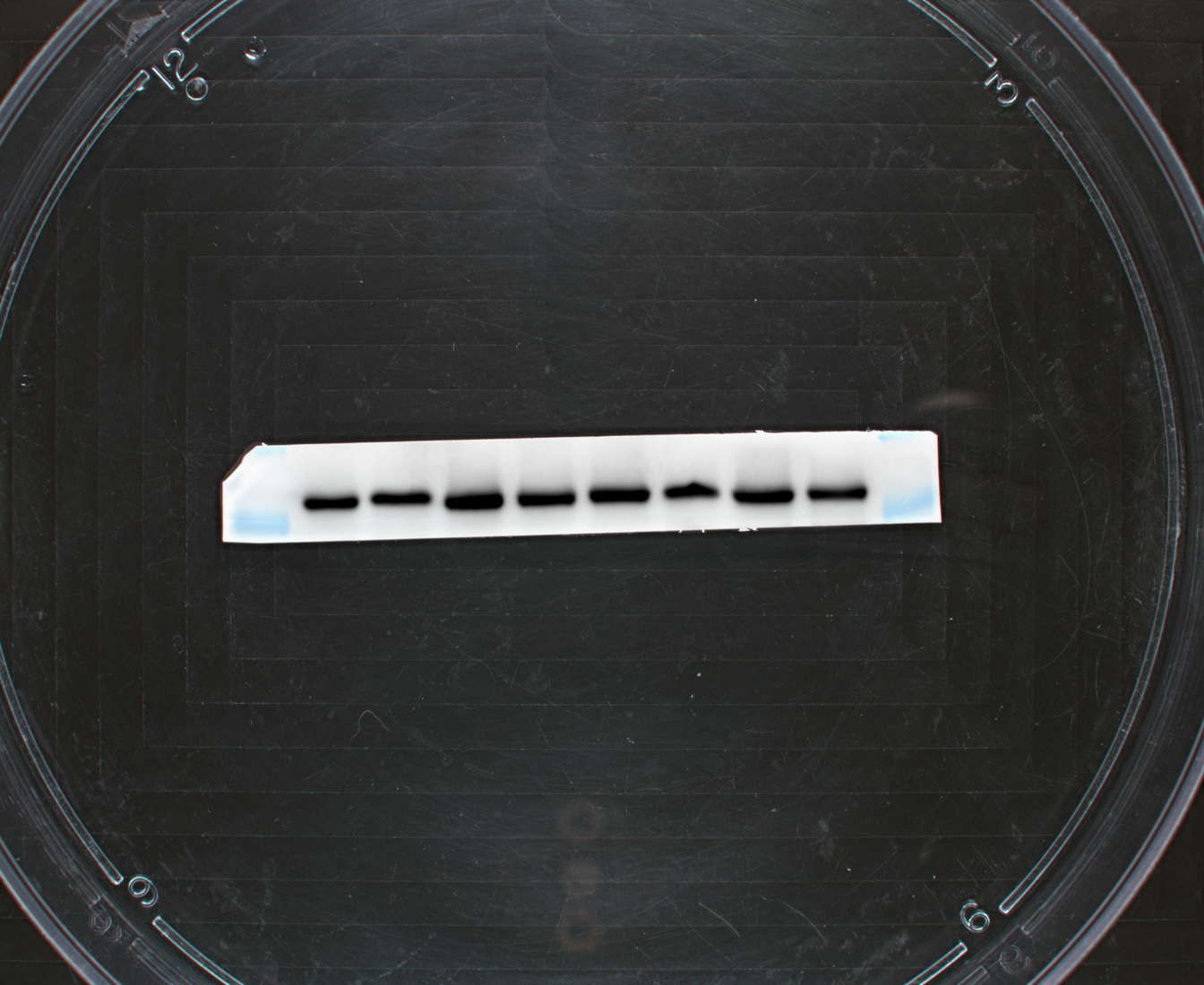

Supplement: Supplementary file 7 [file DataSheet5.ZIP › 3E-GAPDH.tif]

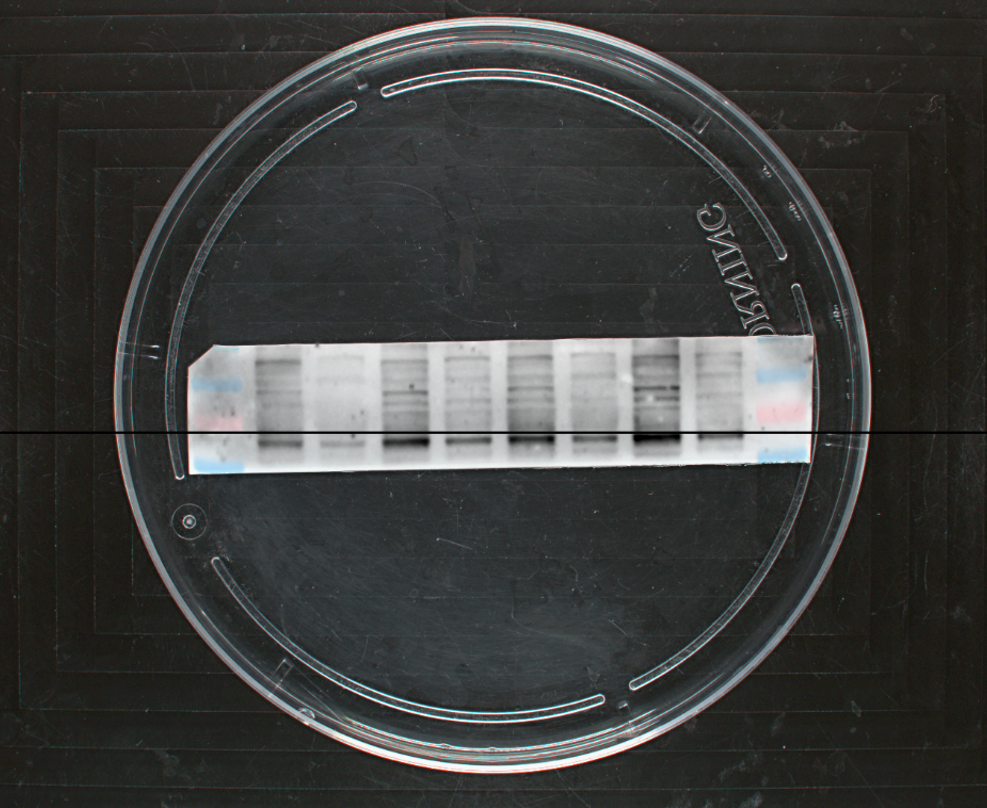

Supplement: Supplementary file 7 [file DataSheet5.ZIP › 3E-MRP1.tif]

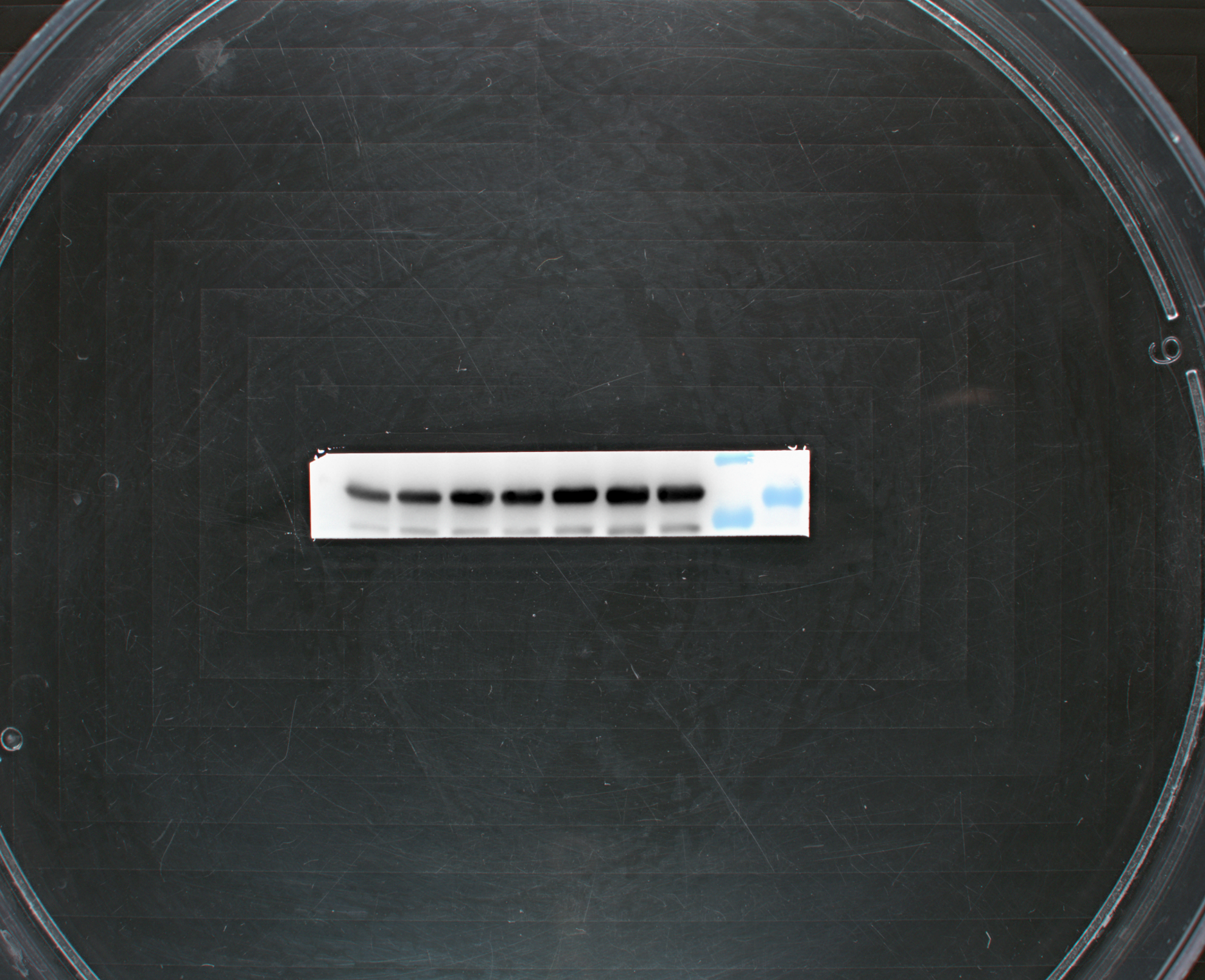

Supplement: Supplementary file 7 [file DataSheet5.ZIP › 3A-GAPDH.tif]

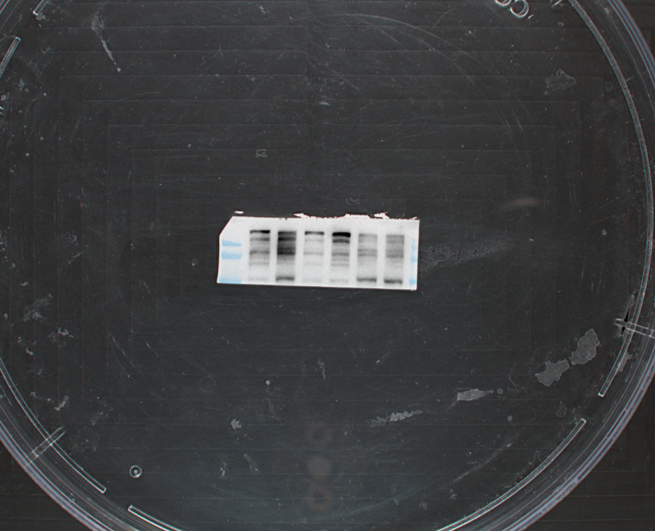

Supplement: Supplementary file 7 [file DataSheet5.ZIP › 3A-MTOR.tif]

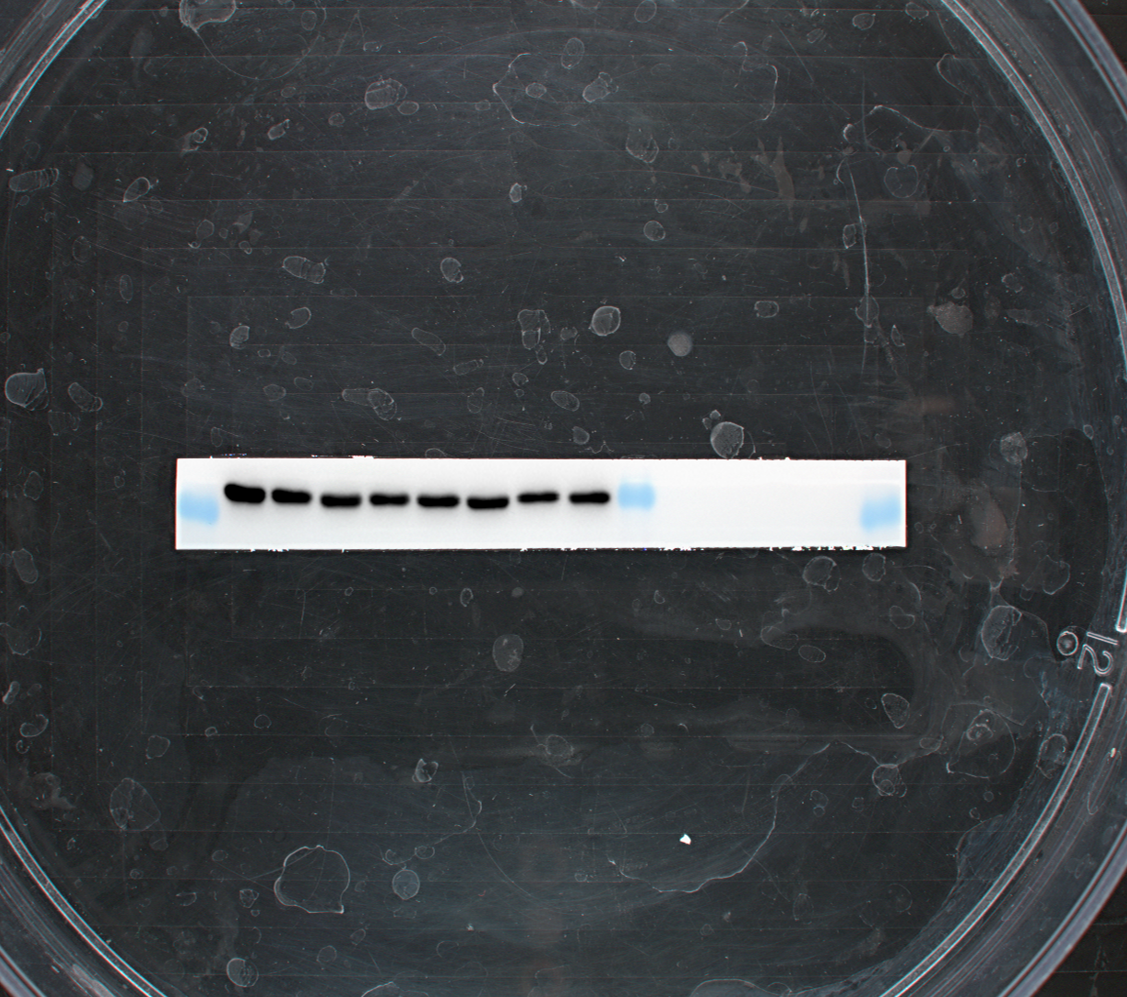

Supplement: Supplementary file 8 [file DataSheet7.ZIP › 5A-GAPDH.tif]

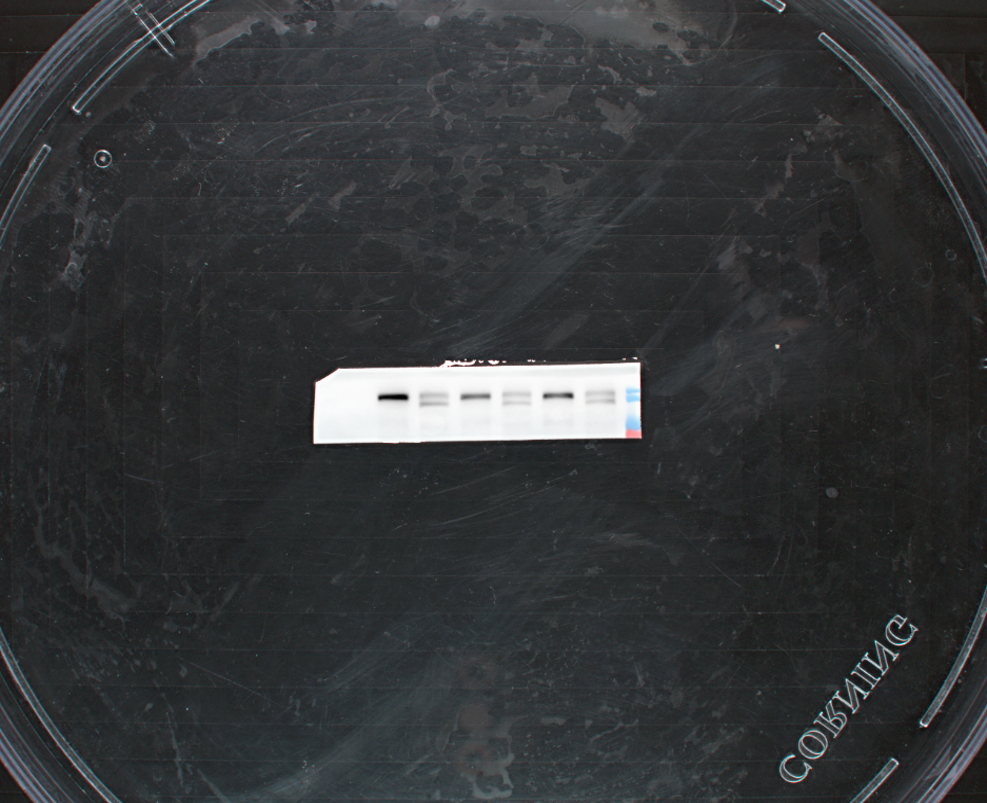

Supplement: Supplementary file 8 [file DataSheet7.ZIP › 5A-P-RAPTOR.tif]

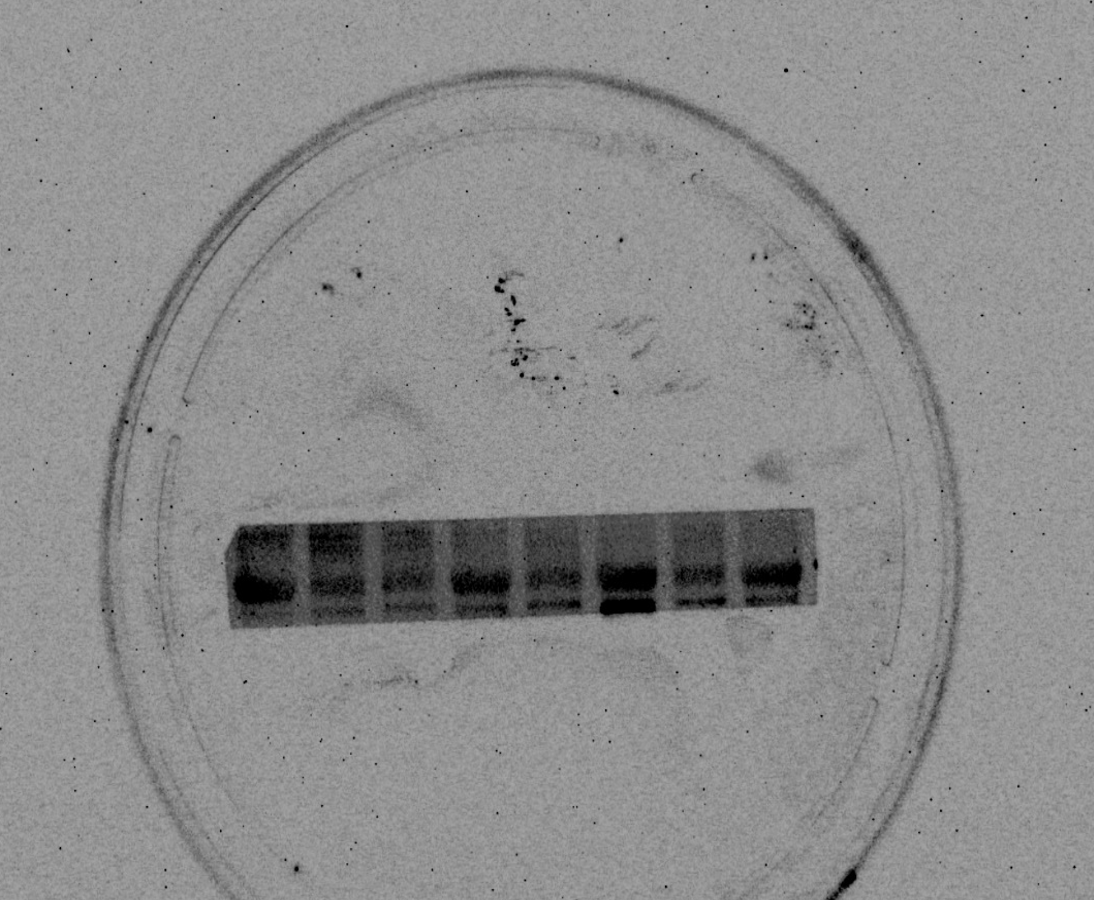

Supplement: Supplementary file 8 [file DataSheet7.ZIP › 5A-P-S6K.tif]

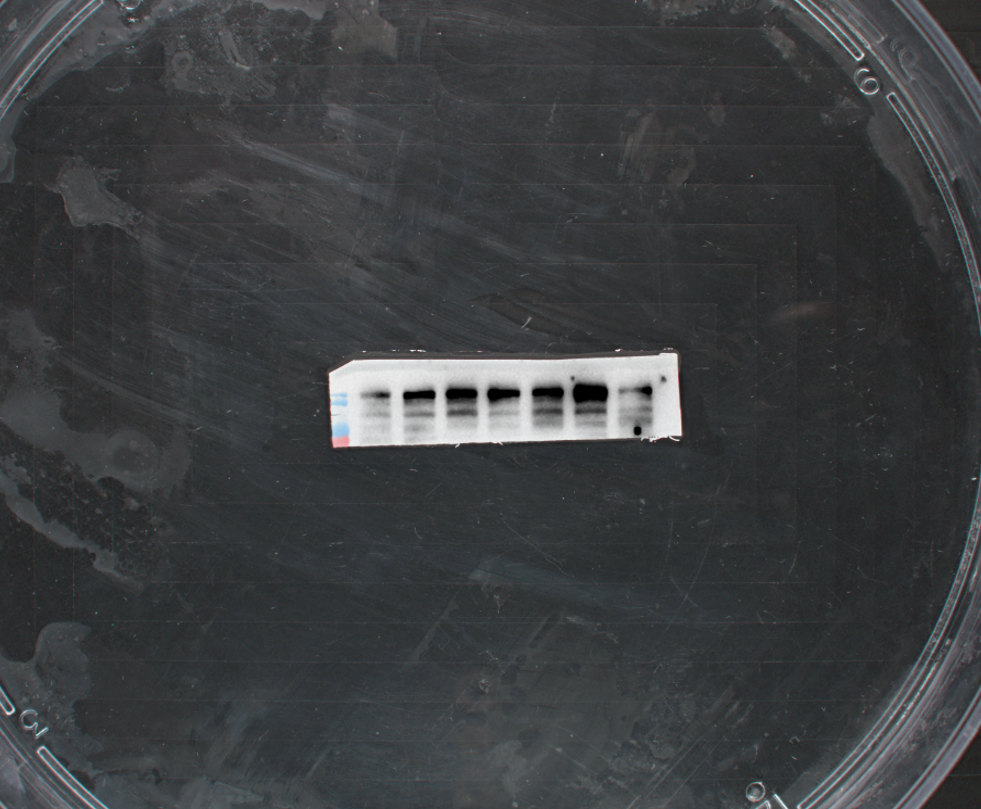

Supplement: Supplementary file 8 [file DataSheet7.ZIP › 5A-RAPTOR.tif]

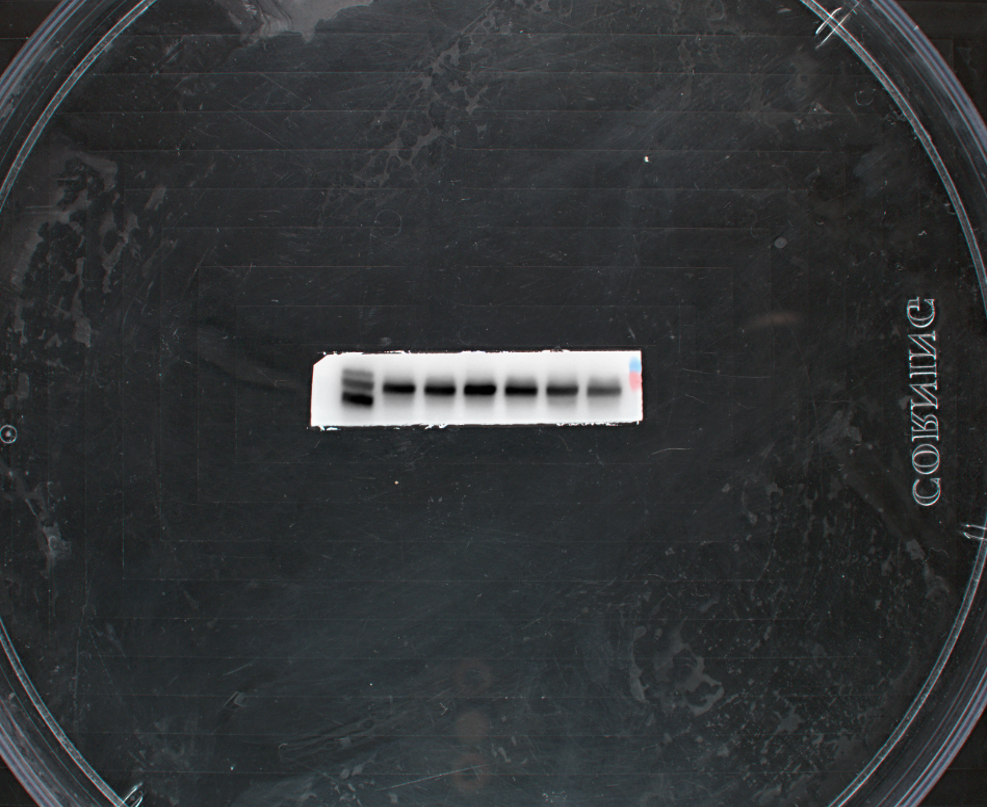

Supplement: Supplementary file 8 [file DataSheet7.ZIP › 5A-S6K.tif]

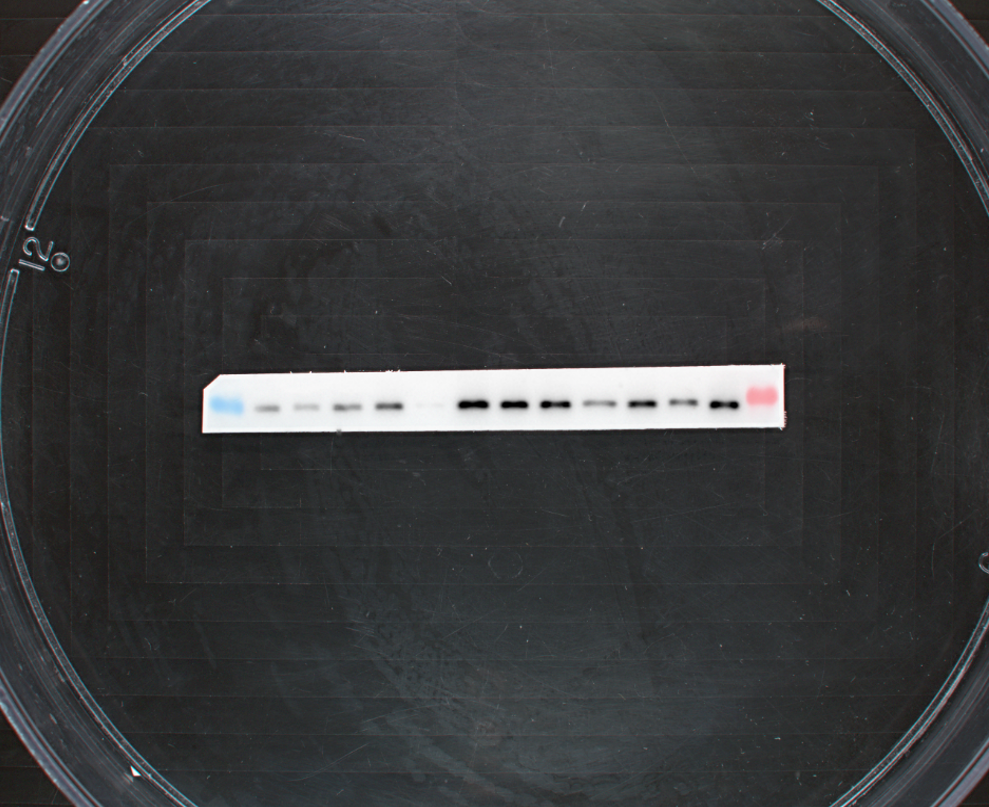

Supplement: Supplementary file 8 [file DataSheet7.ZIP › 5C-EIF4E.tif]

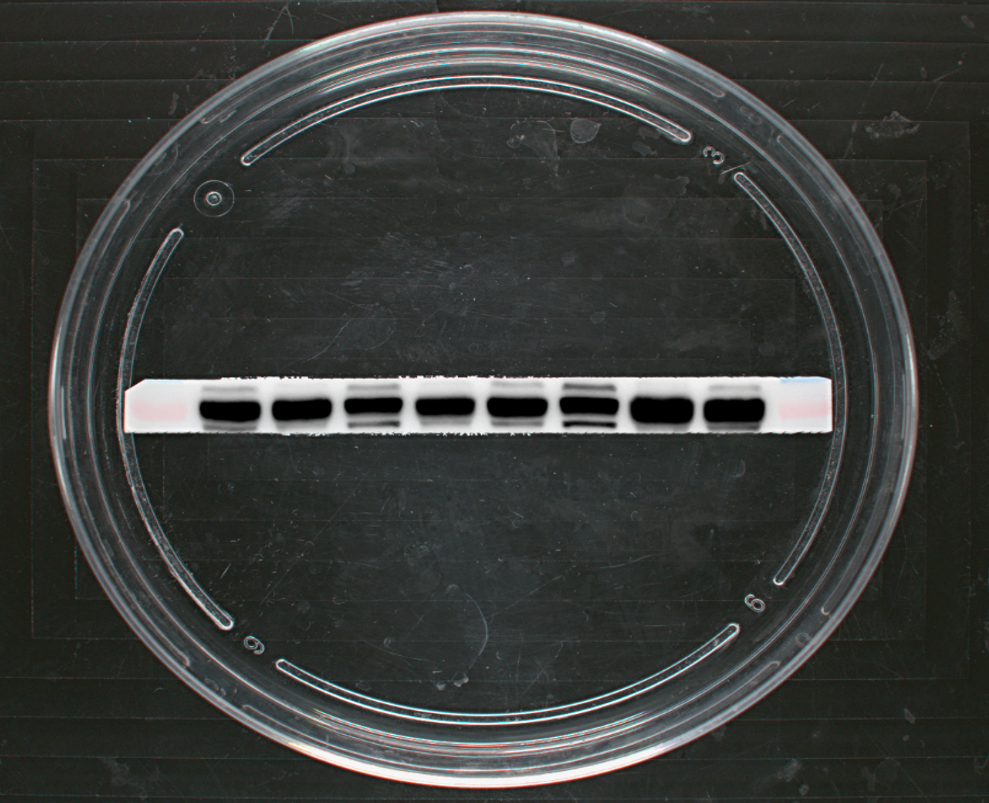

Supplement: Supplementary file 8 [file DataSheet7.ZIP › 5C-GAPDH.tif]

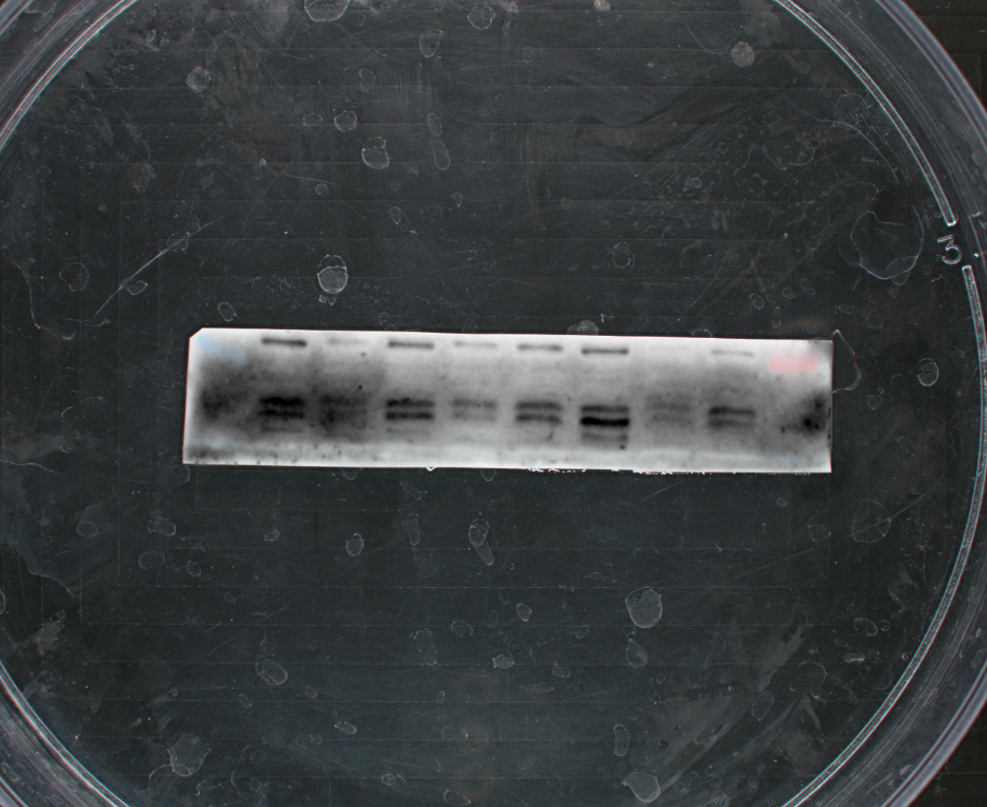

Supplement: Supplementary file 8 [file DataSheet7.ZIP › 5C-P-4EBP1.tif]

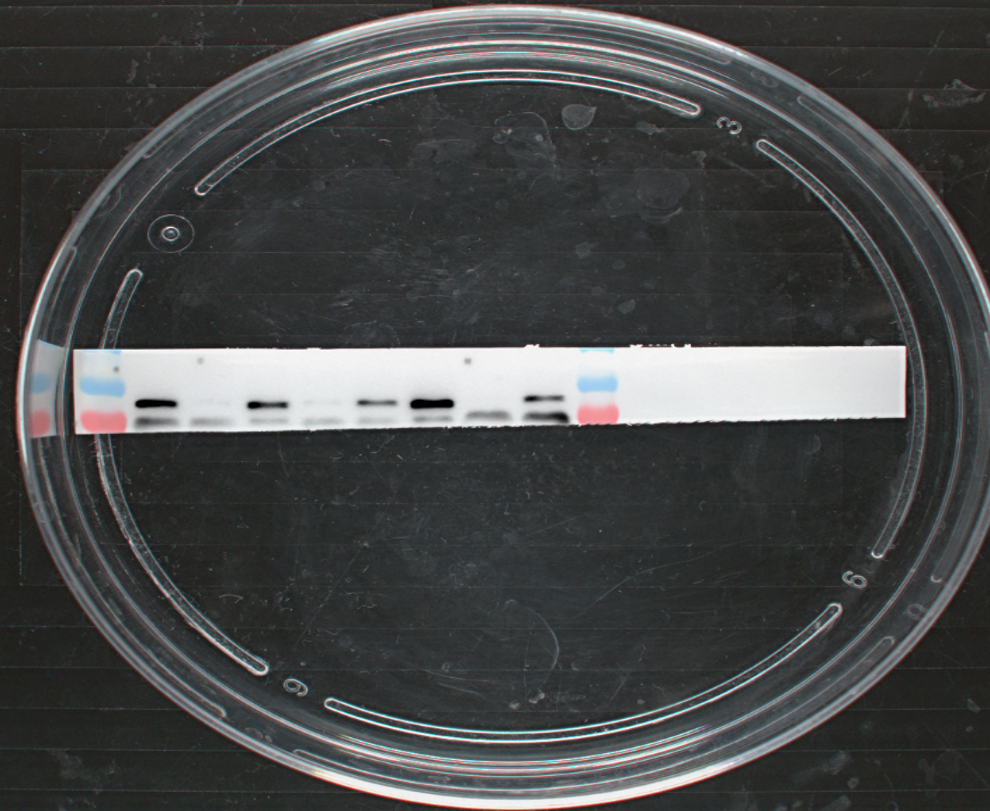

Supplement: Supplementary file 8 [file DataSheet7.ZIP › 5C-P-S6K.tif]

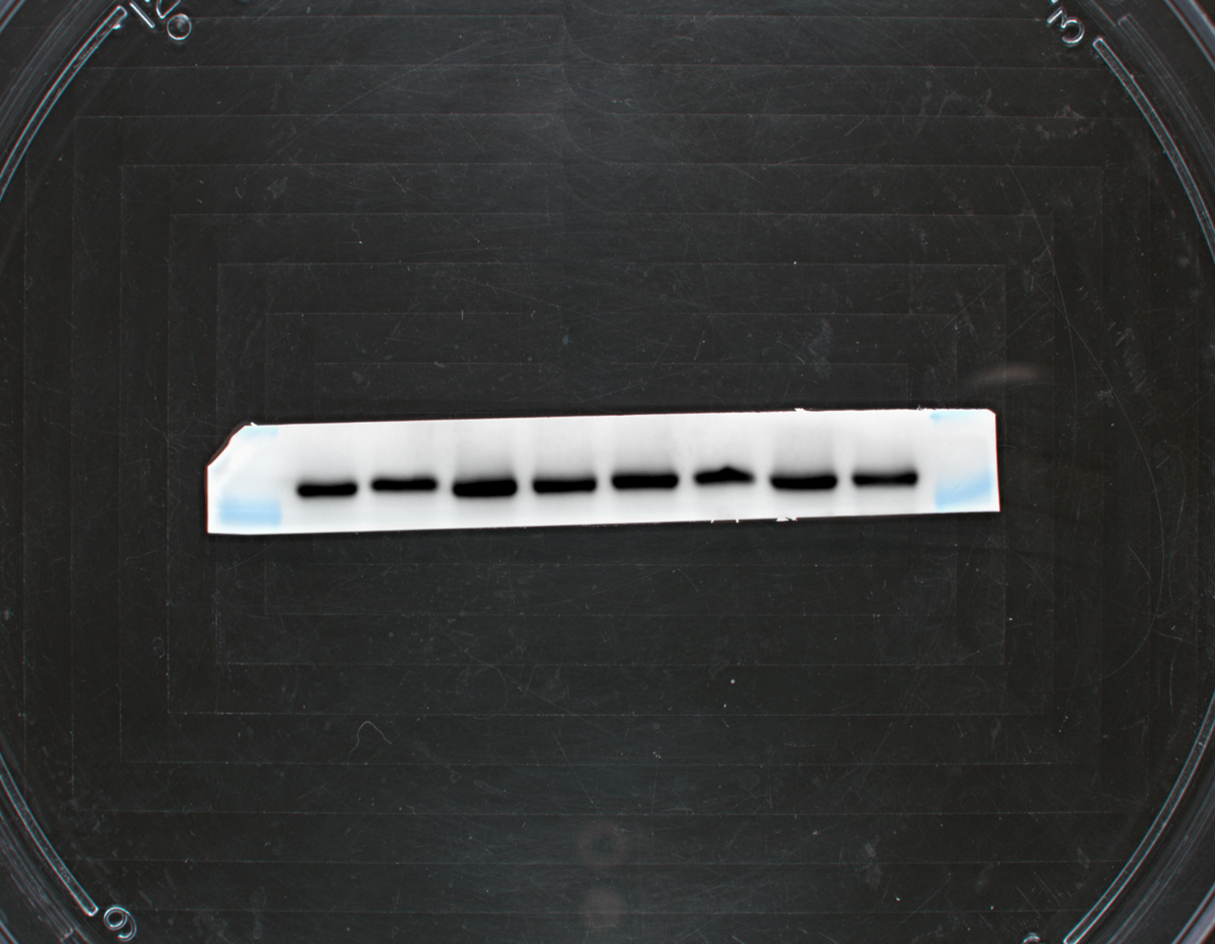

Supplement: Supplementary file 8 [file DataSheet7.ZIP › 5D-GAPDH.tif]

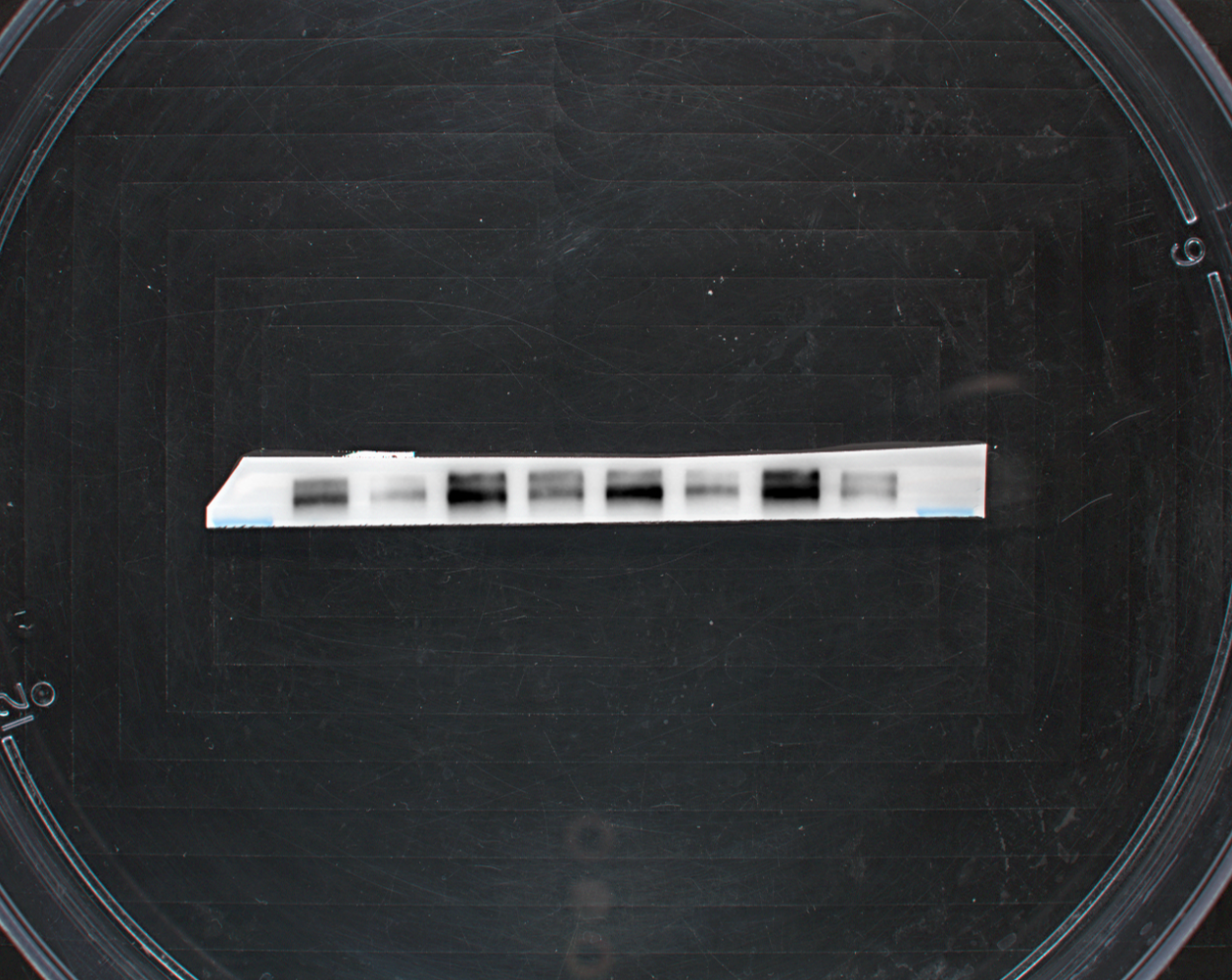

Supplement: Supplementary file 8 [file DataSheet7.ZIP › 5D-MRP1.tif]

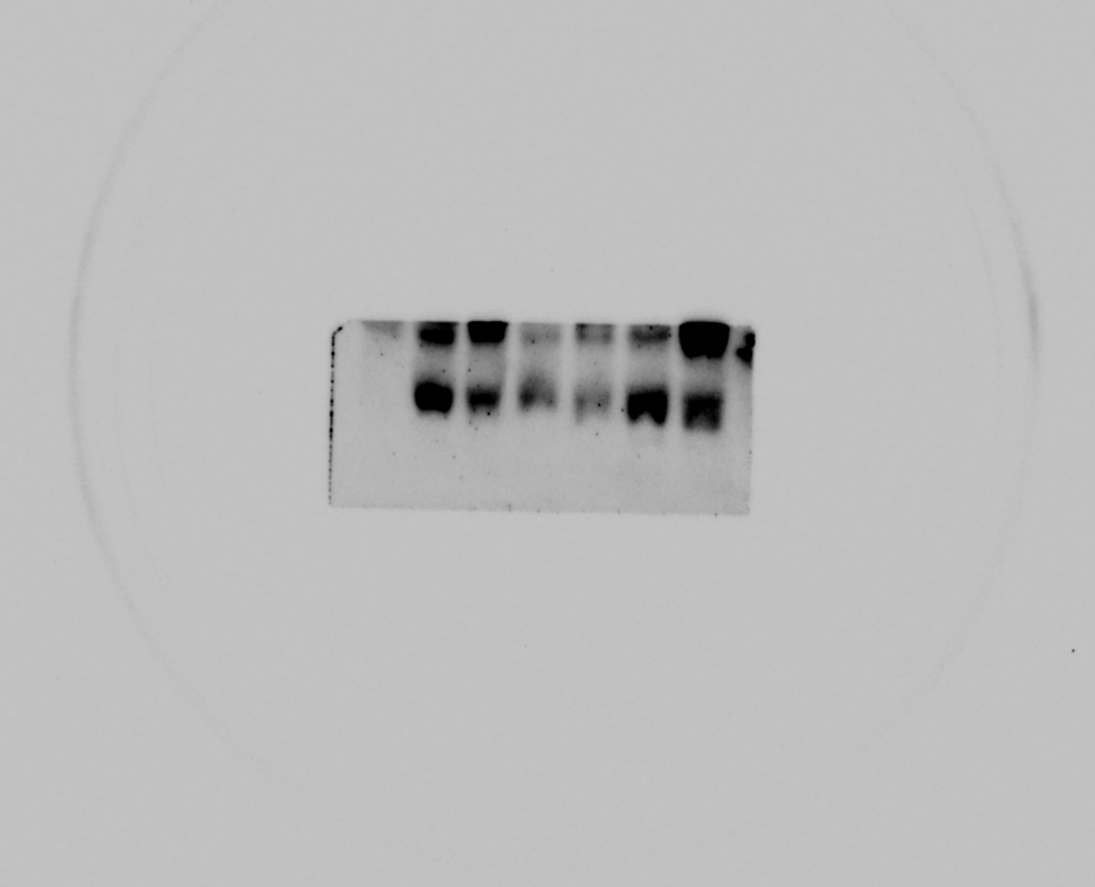

Supplement: Supplementary file 8 [file DataSheet7.ZIP › 5A-4EBP1.tif]

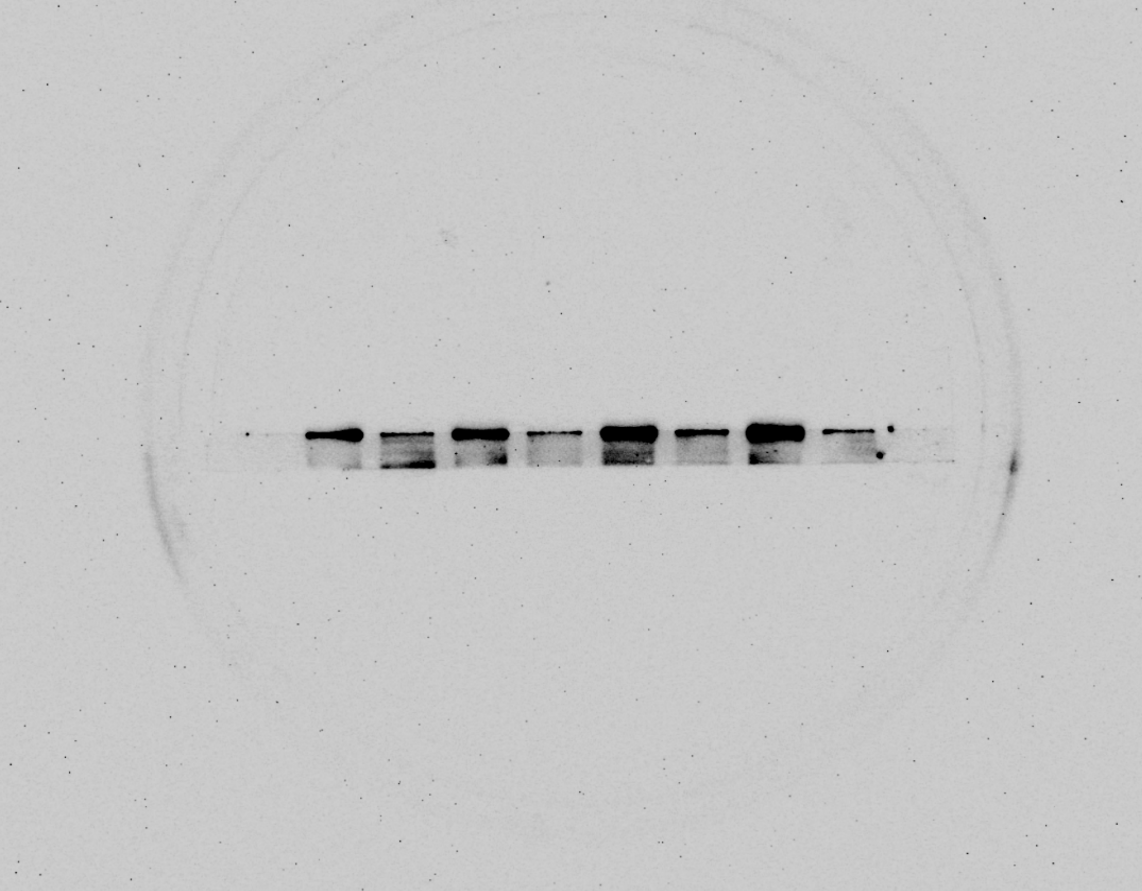

Supplement: Supplementary file 8 [file DataSheet7.ZIP › 5A-EIF4E.tif]
