# Supplementary material for: Ad-Apoptin-hTERTp-E1a Regulates Autophagy Through the AMPK-mTOR-eIF4F Signaling Axis to Reduce Drug Resistance of MCF-7/ADR Cells
Source: Front Mol Biosci. 2021 Nov 19;8:763500. doi: 10.3389/fmolb.2021.763500 (PMC8640141; doi:10.3389/fmolb.2021.763500)
Supplement: Supplementary file 5 [file DataSheet2.ZIP › fig3-microscopy images.pptx]

## Slide 1
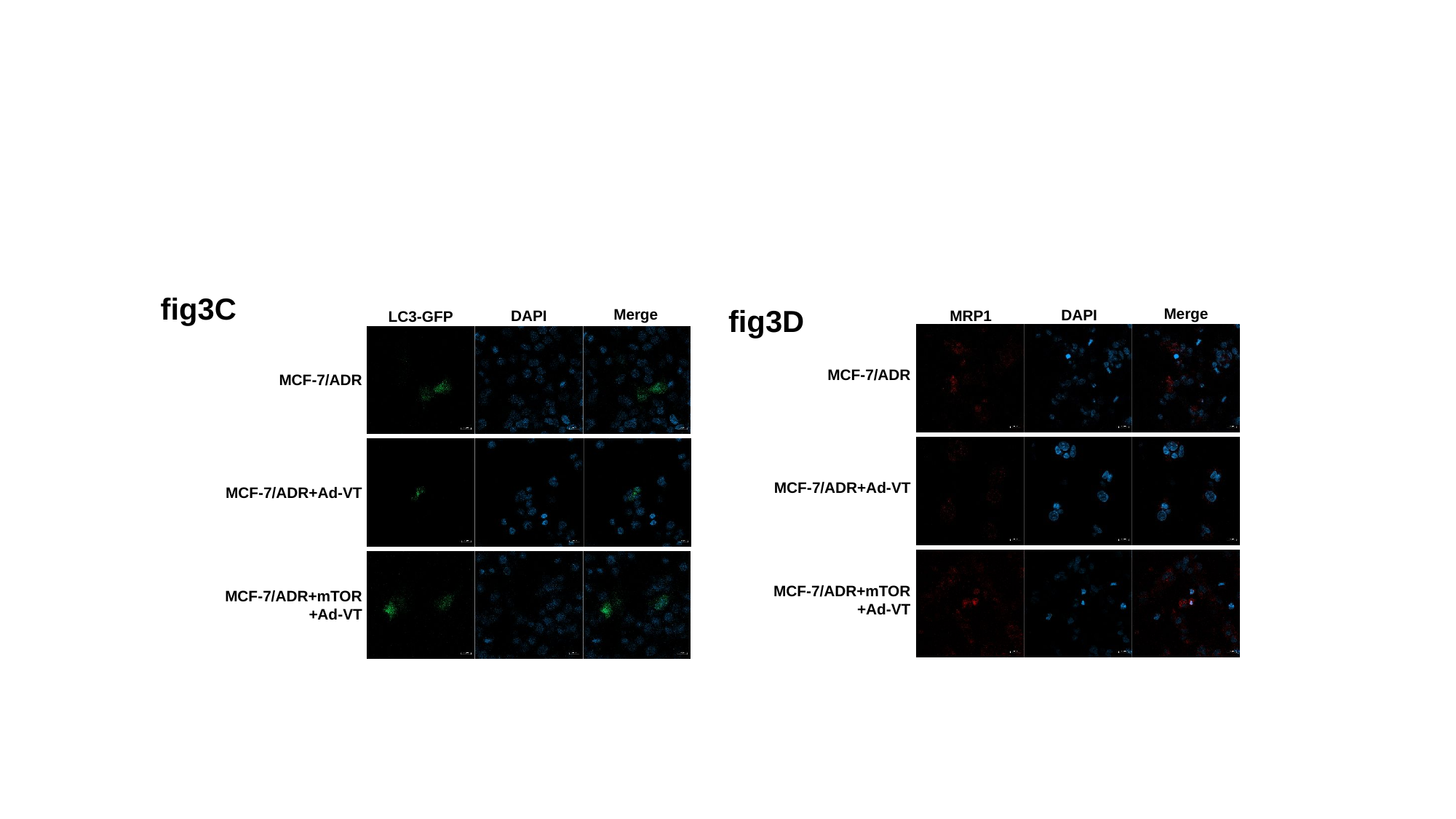

fig3C
fig3D
Merge
Merge
DAPI
MRP1
DAPI
LC3-GFP
MCF-7/ADR
MCF-7/ADR
MCF-7/ADR+Ad-VT
MCF-7/ADR+Ad-VT
MCF-7/ADR+mTOR
+Ad-VT
MCF-7/ADR+mTOR
+Ad-VT
