# Supplementary material for: Ad-Apoptin-hTERTp-E1a Regulates Autophagy Through the AMPK-mTOR-eIF4F Signaling Axis to Reduce Drug Resistance of MCF-7/ADR Cells
Source: Front Mol Biosci. 2021 Nov 19;8:763500. doi: 10.3389/fmolb.2021.763500 (PMC8640141; doi:10.3389/fmolb.2021.763500)
Supplement: Supplementary file 5 [file DataSheet2.ZIP › fig2-microscopy images.pptx]

## Slide 1
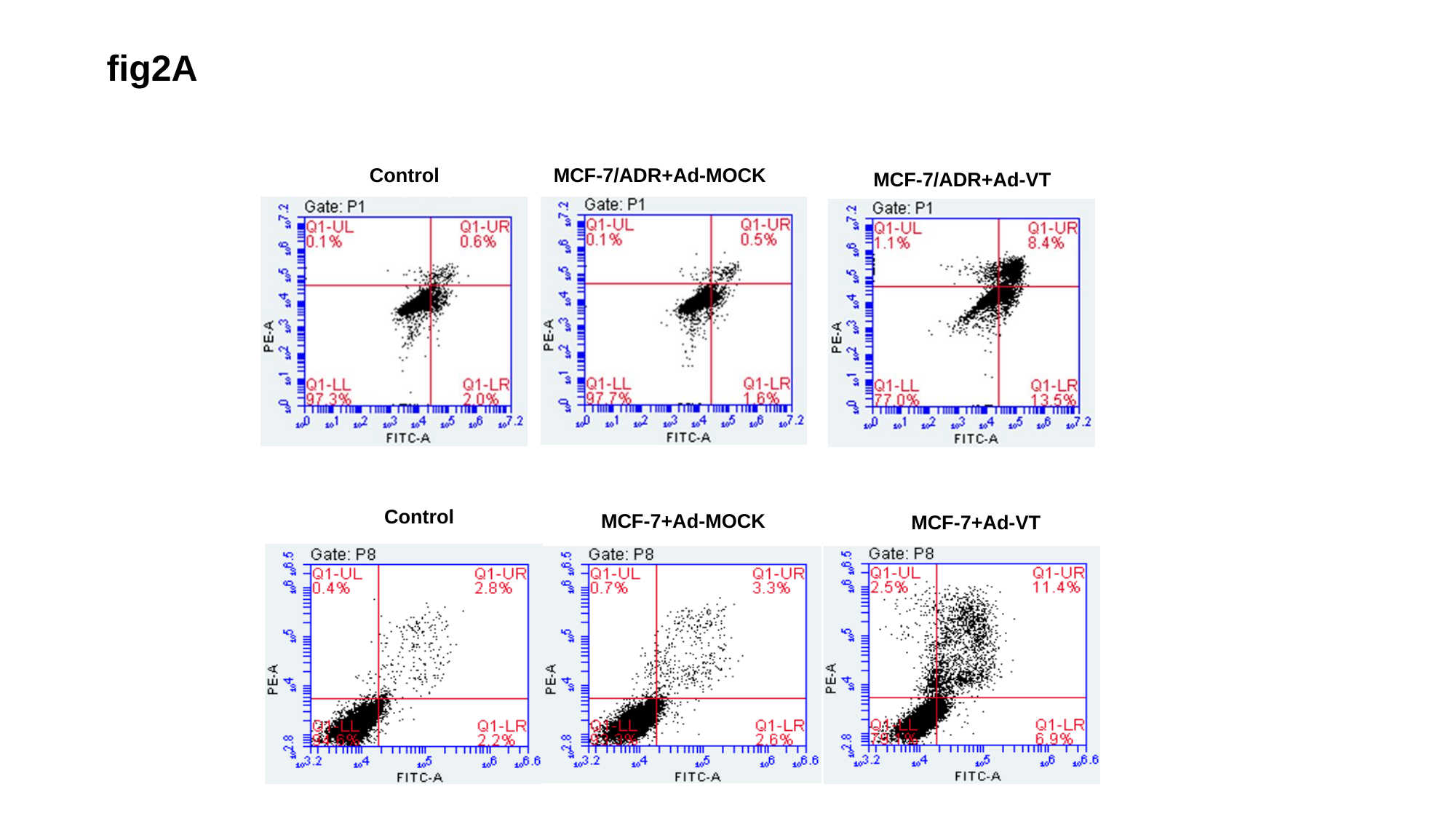

fig2A
MCF-7/ADR+Ad-MOCK
Control
MCF-7/ADR+Ad-VT
Control
MCF-7+Ad-MOCK
MCF-7+Ad-VT

## Slide 2
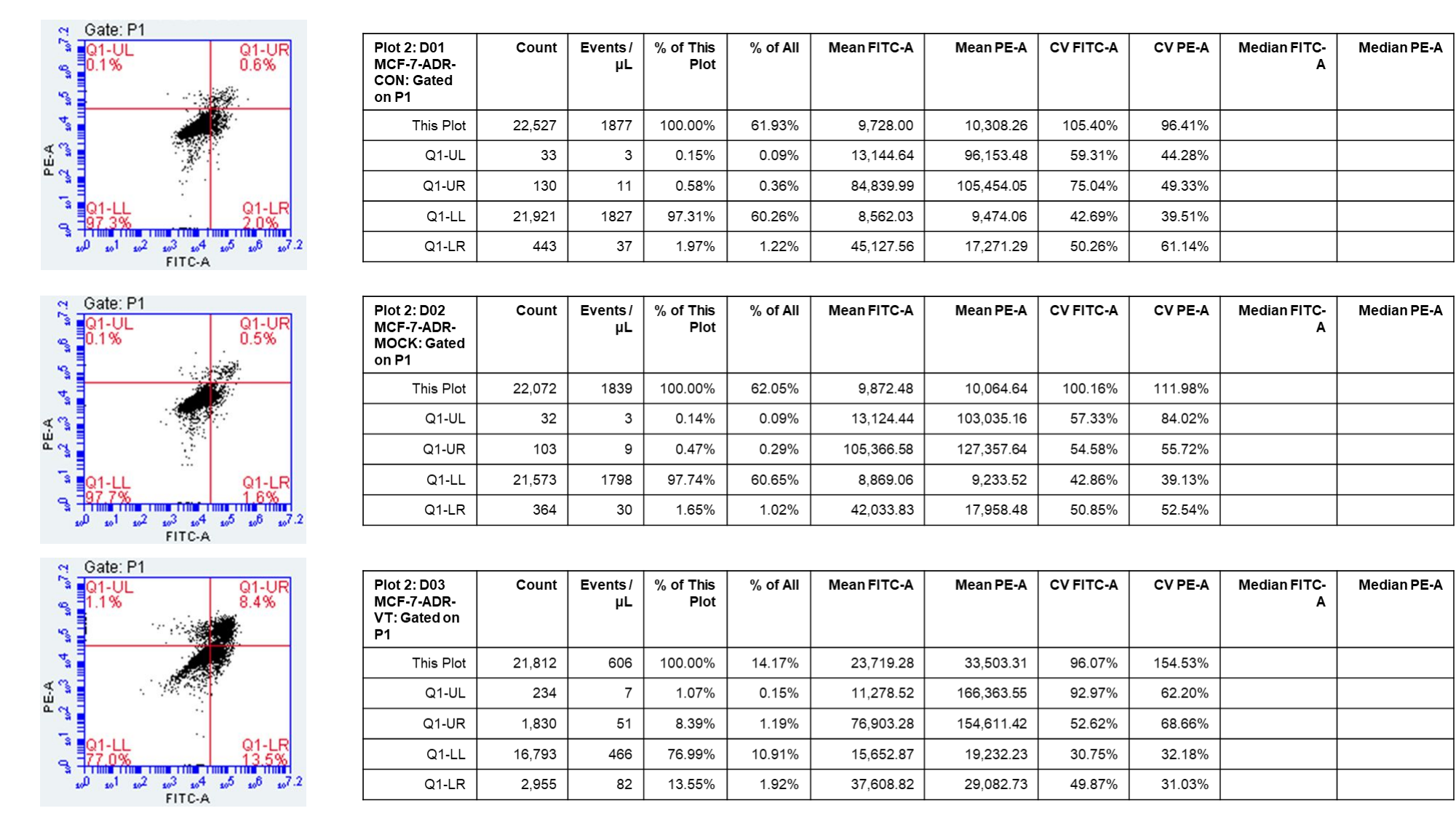

## Slide 3
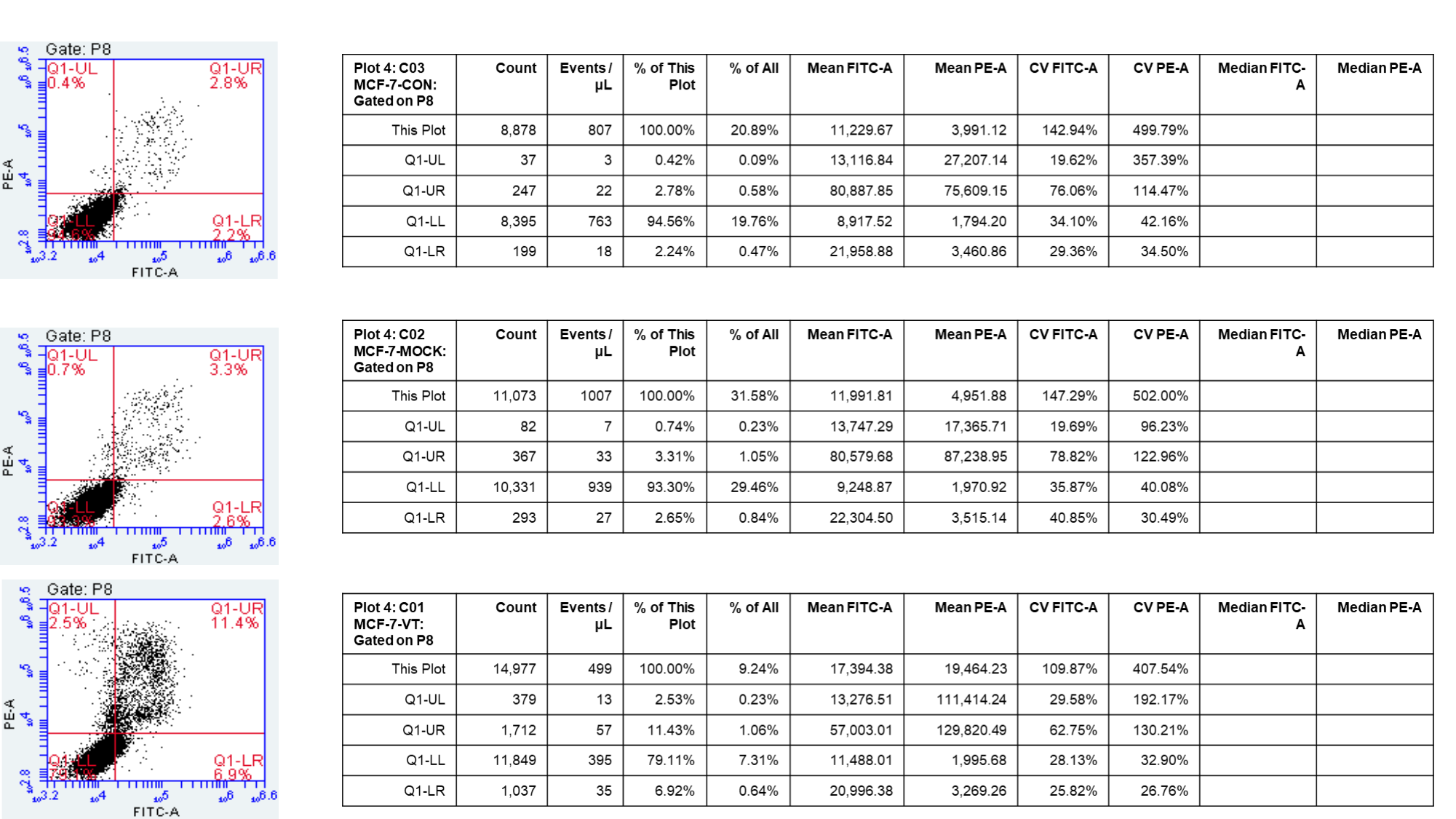

## Slide 4
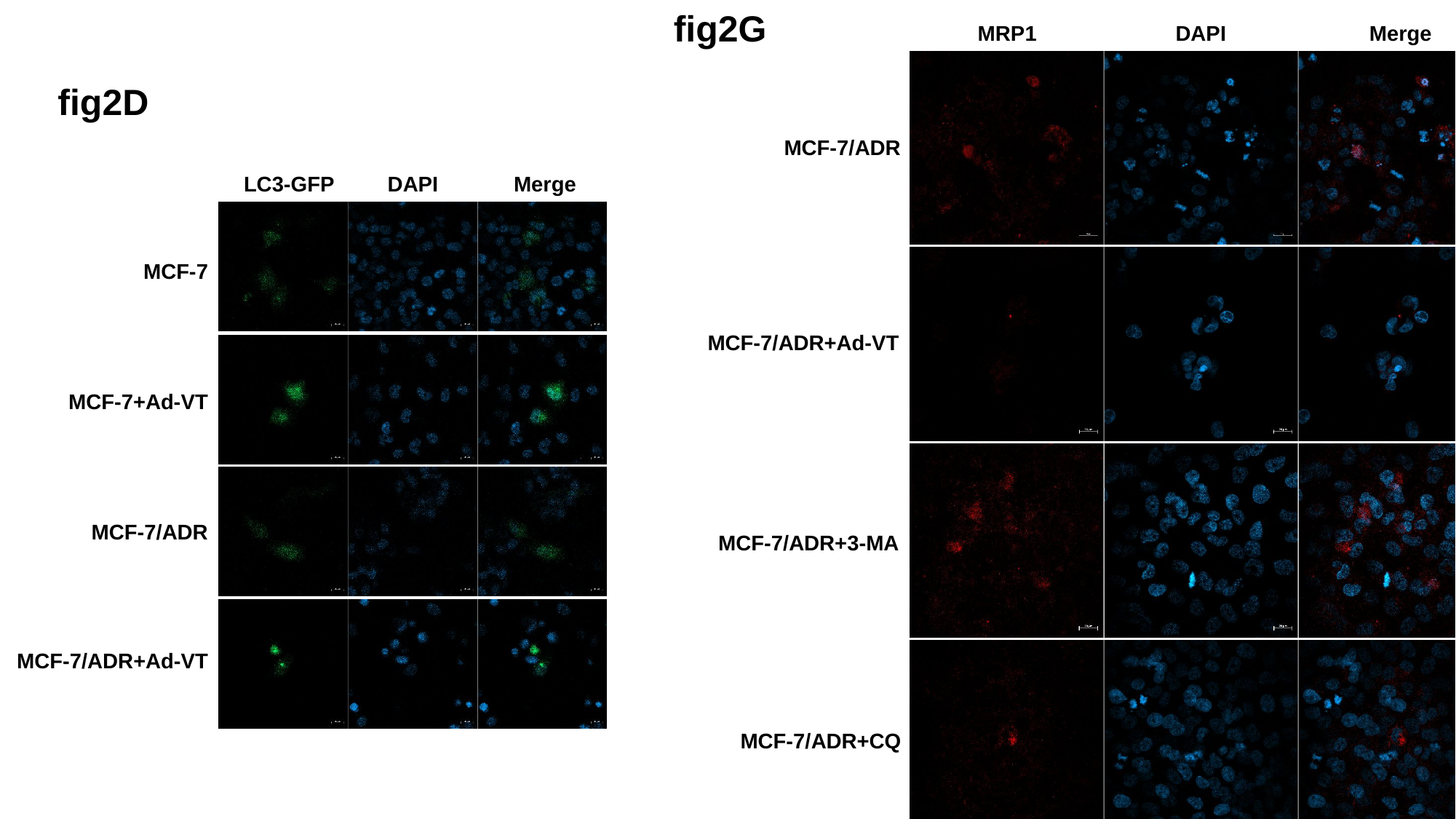

fig2G
DAPI
Merge
MRP1
fig2D
MCF-7/ADR
LC3-GFP
DAPI
Merge
MCF-7
MCF-7/ADR+Ad-VT
MCF-7+Ad-VT
MCF-7/ADR
MCF-7/ADR+3-MA
MCF-7/ADR+Ad-VT
MCF-7/ADR+CQ
